# Supplementary material for: Incomplete lineage sorting of segmental duplications defines the human chromosome 2 fusion site early during African great ape speciation
Source: Cell Genom. 2025 Dec 2;6(1):101079. doi: 10.1016/j.xgen.2025.101079 (PMC12926204; doi:10.1016/j.xgen.2025.101079)
Supplement: Document S1. Figures S1–S30 [file mmc1.pdf]

Cell Genomics, Volume *6*

Supplemental information

Incomplete lineage sorting of segmental duplications defines the human chromosome 2 fusion site early during African great ape speciation

Zikun Yang, Lu Zhang, Xinrui Jiang, Xiangyu Yang, Kaiyue Ma, DongAhn Yoo, Yong Lu, Shilong Zhang, Jieyi Chen, Yanhong Nie, Xinyan Bian, Junmin Han, Lianting Fu, Juan Zhang, Mario Ventura, Guojie Zhang, Qiang Sun, Evan E. Eichler, and Yafei Mao

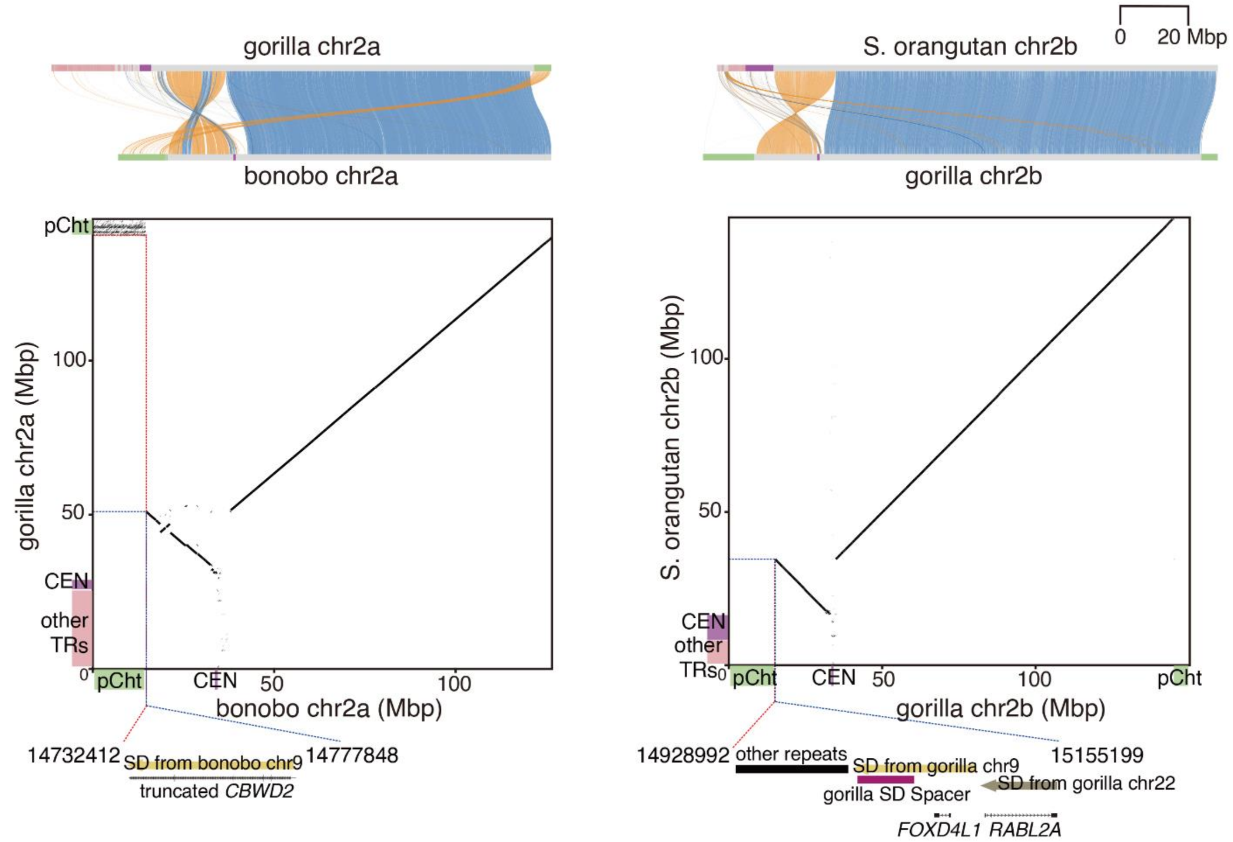

Figure S1. The syntenic comparison of primate chromosome 2 pericentric inversions, related to Figure 1. The red dotted line indicates the start of the pericentric breakpoints, while the blue dotted line marks their end. The segmental duplications (SDs) associated with breakpoints are shown on the bottom. S. orangutan represents Sumatran orangutan.

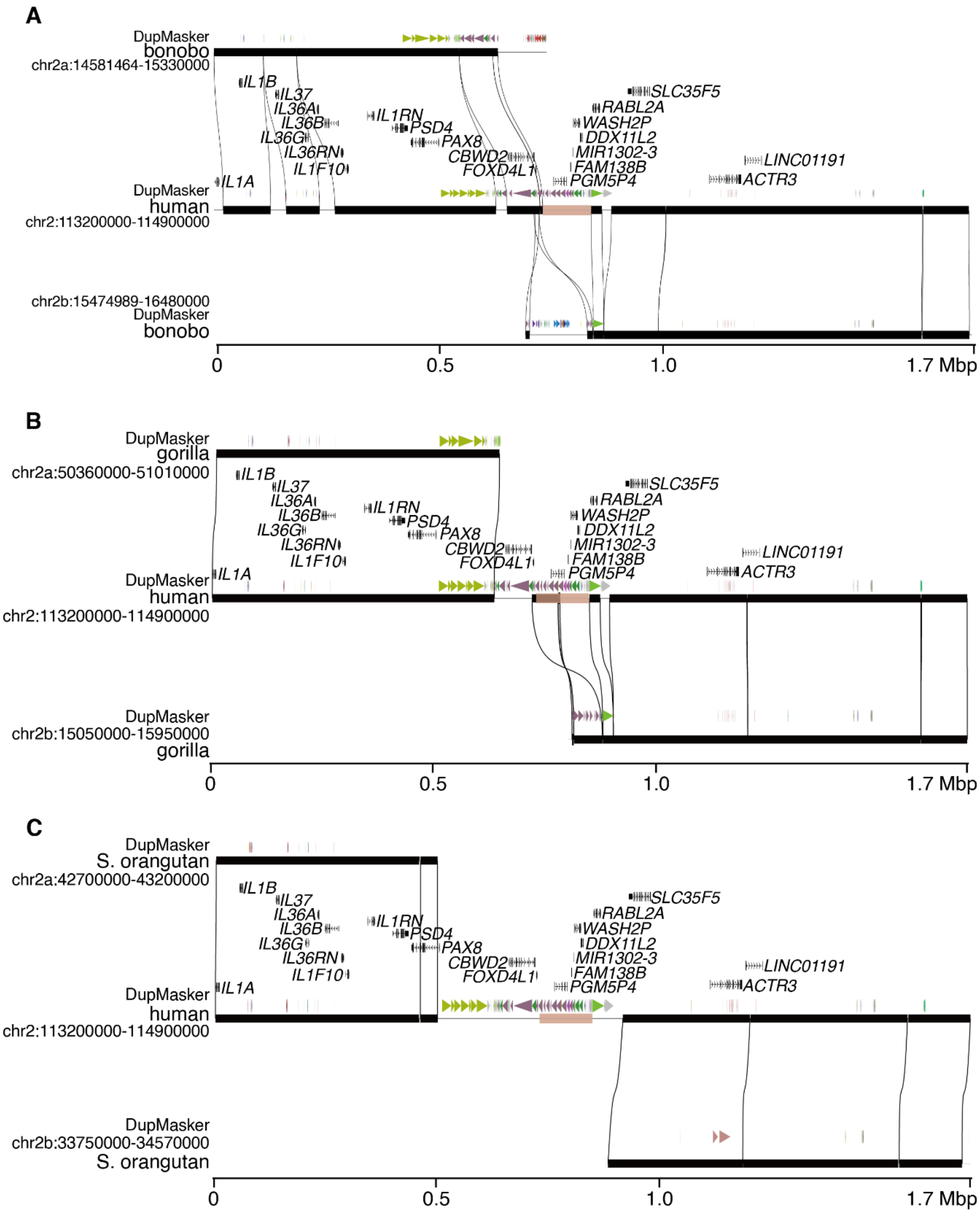

Figure S2. The comparative analysis of primate chromosome 2 and the human fusion site, related to Figure 1. Minimiro comparisons between the human fusion site and those in bonobo (A), gorilla (B), and S. orangutan (C). Gene and SD annotations are displayed on each chromosome.

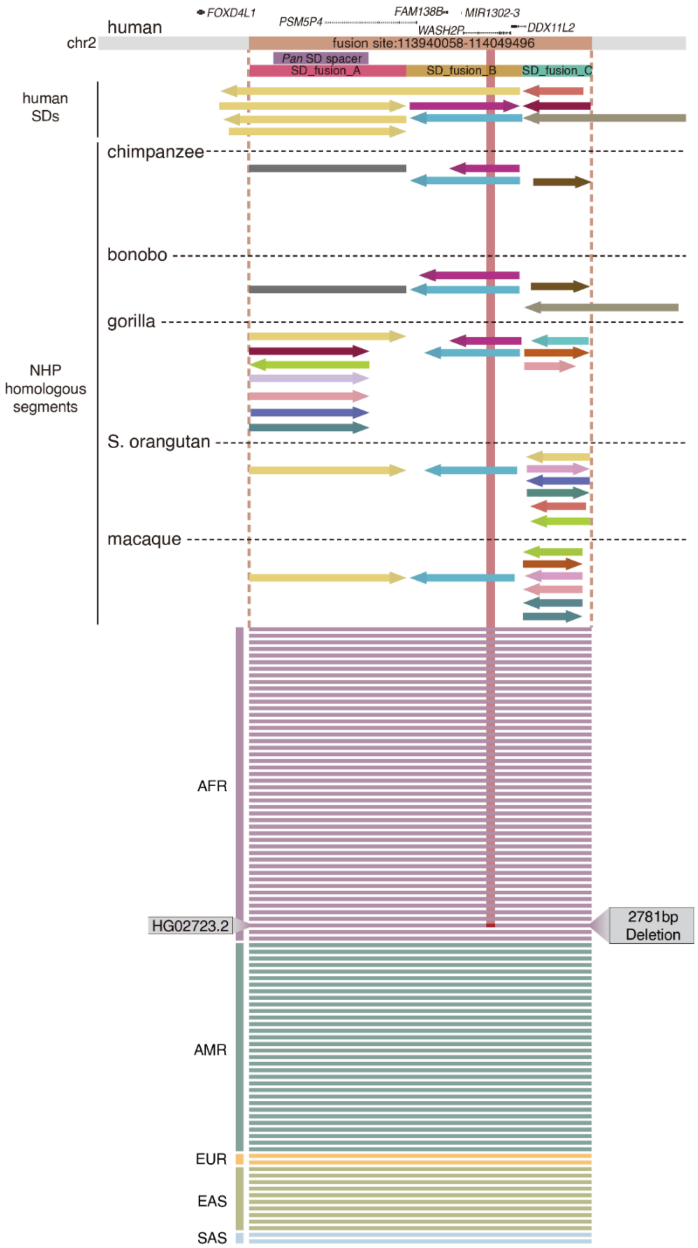

Figure S3. The structure of the fusion site in Human Pangenome Reference Consortium (HPRC) samples, related to Figure 2. The top panel shows the structure of the fusion site (related to Figure S5), while the bottom panel shows the schematic genomic structure of each HPRC sample. Structural variants (SVs) longer than 1 kbp are shown in red boxes. Only a 2,781 bp deletion in HG02723 haplotype 2 is identified, suggesting a highly conserved structure of the fusion sites in human populations.

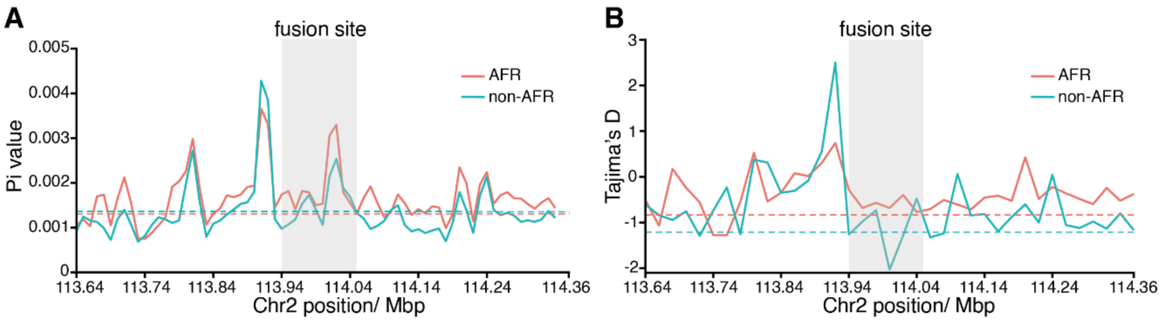

Figure S4. Population genetic statistics of the human fusion site, related to Figure 2. The π diversity (A) and Tajima's D (B) are estimated using long-read human genome assemblies of African (red, n=102) and non-African (blue, n=436) populations. The dashed lines show the average values for human chromosome 2.

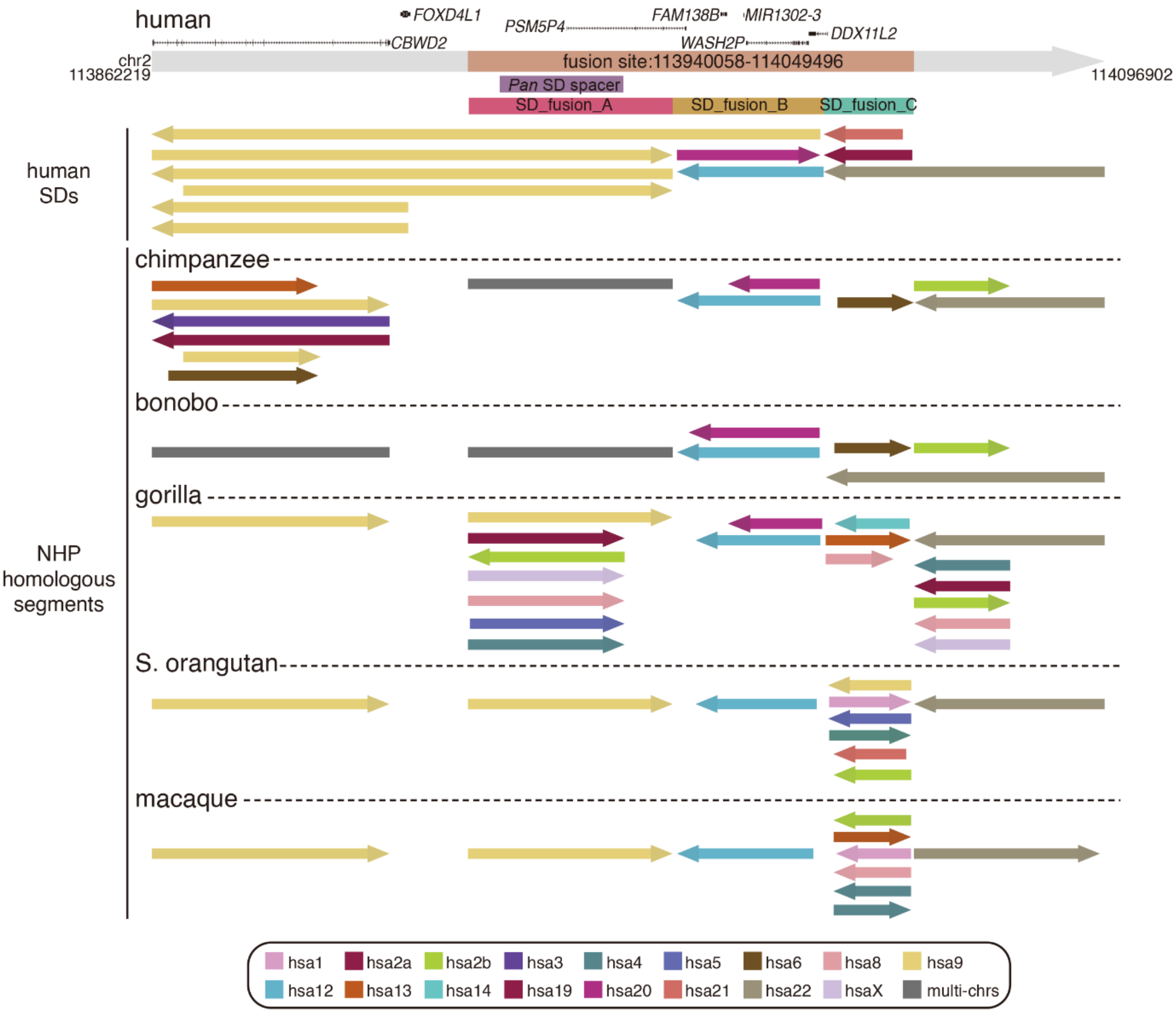

Figure S5. The human fusion site comprises distinct SDs, related to Figure 2. A human genomic segment (chr2:113,862,219-114,096,902) is shown in grey, with the human fusion site (chr2:113,940,058-114,049,496) highlighted in amber. Homologous segments in nonhuman primates (chimpanzee, bonobo, gorilla, orangutan, and macaque) are also depicted in corresponding order. Dark grey blocks represent multi-chromosomes in the same region; see Table S3.

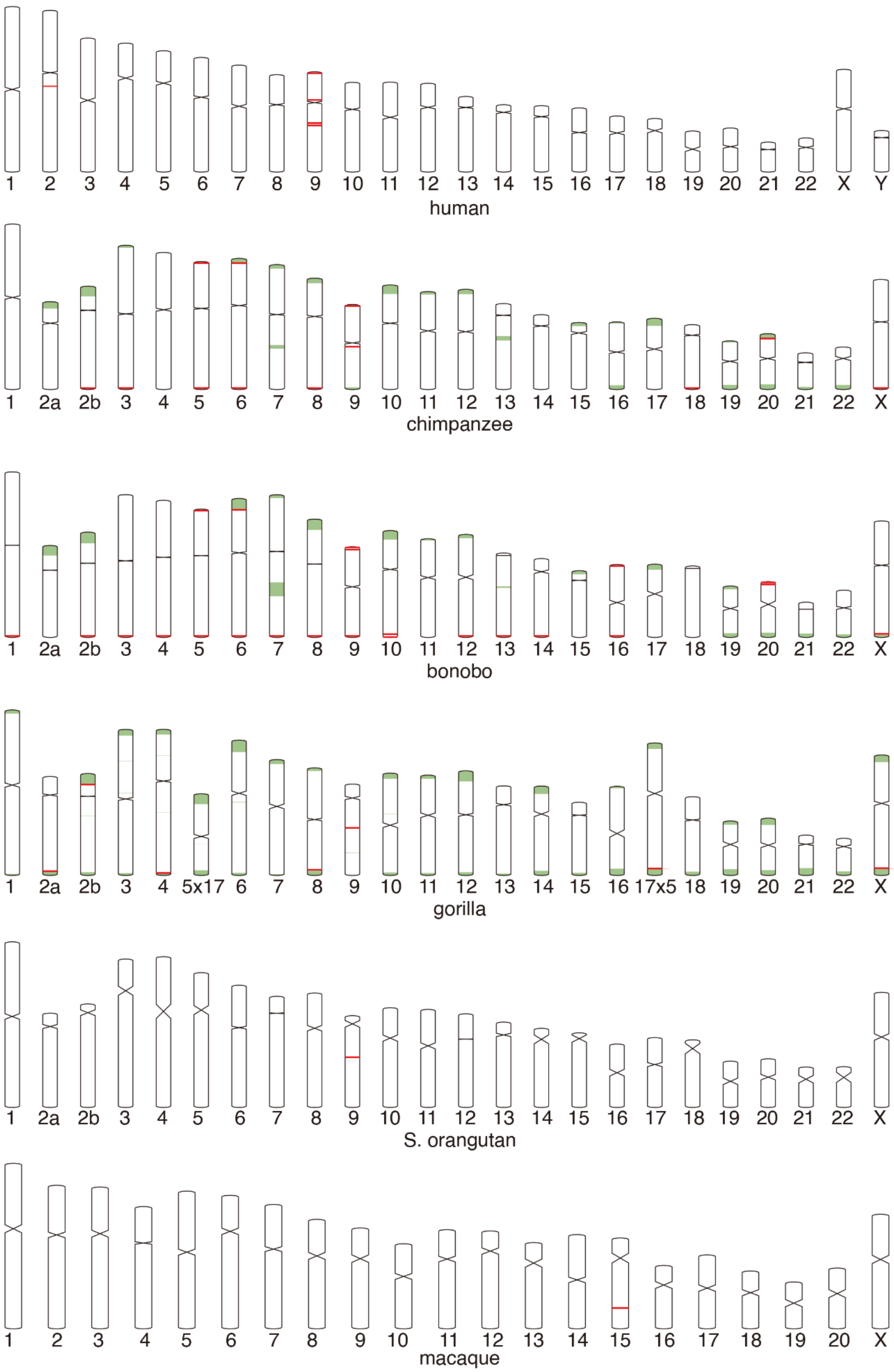

Figure S6. The ideogram of homologous segments in human and NHPs for SD_fusion_A (chr2: 113,940,058-113,990,477), related to Figure 2. Red blocks represent homologous segments and green blocks represent pCht.

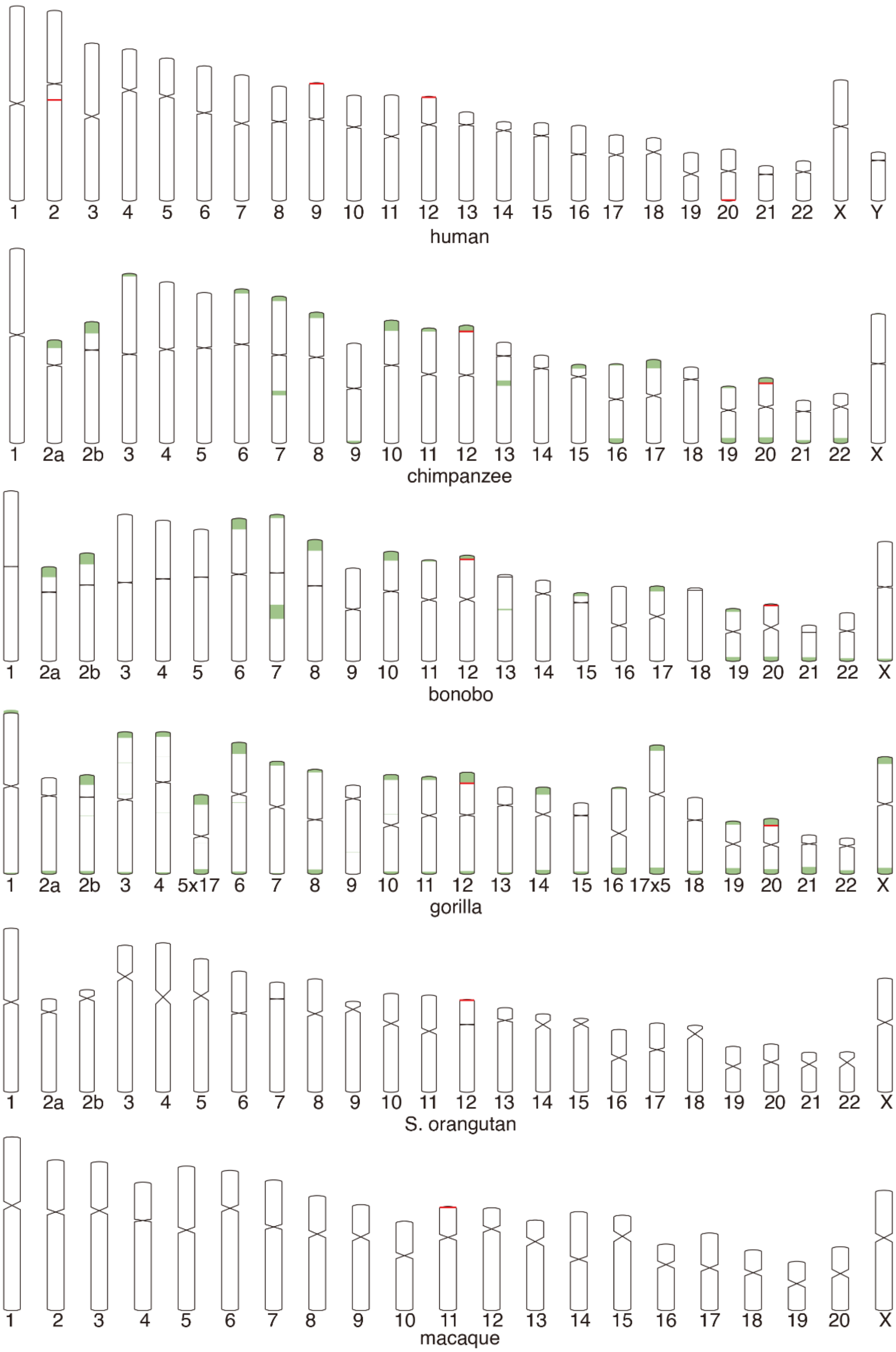

Figure S7. The ideogram of homologous segments in human and NHPs for SD_fusion_B (chr2:113,991,553-114,027,657), related to Figure 2. Red blocks represent homologous segments and green blocks represent pCht.

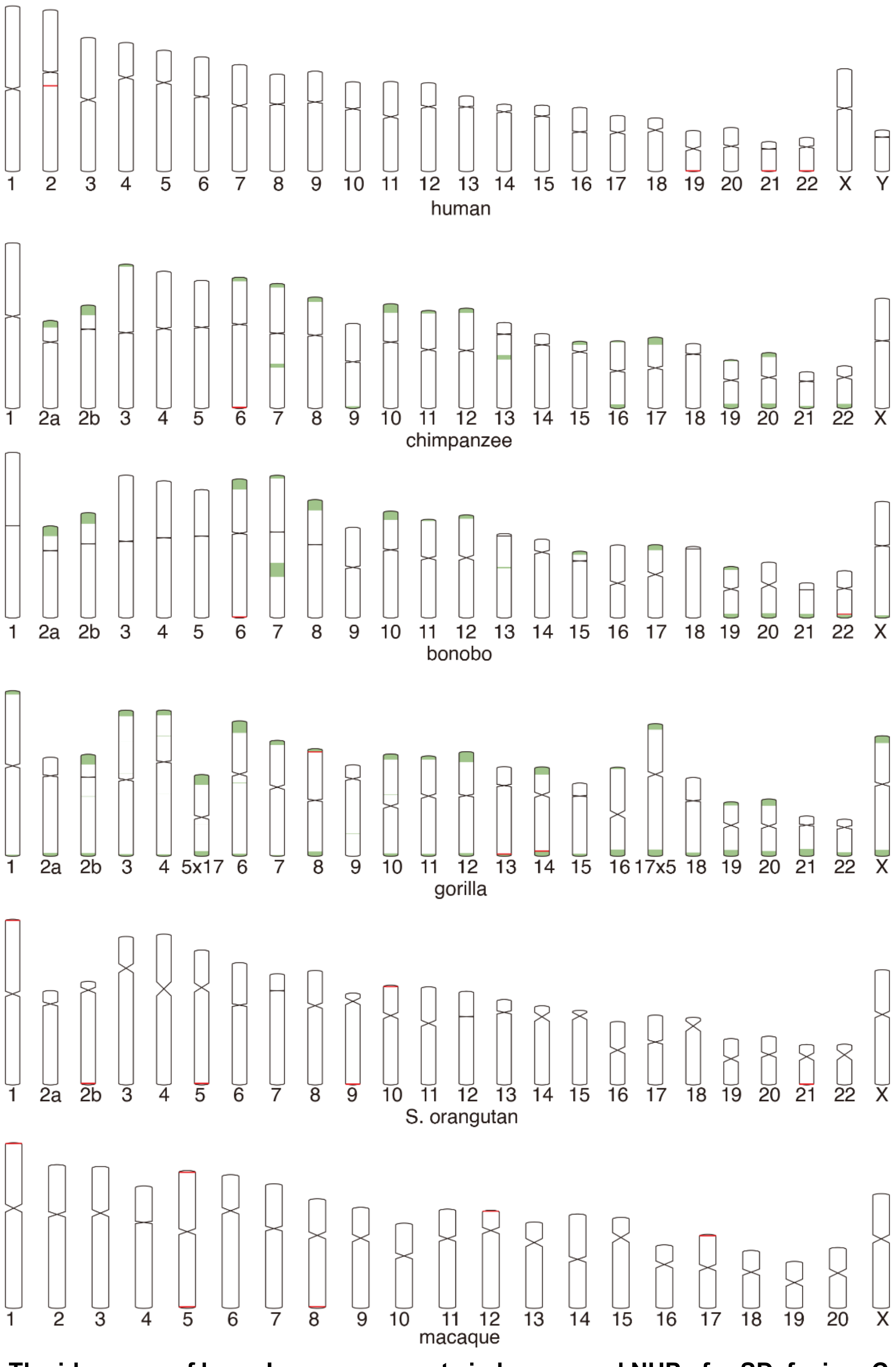

Figure S8. The ideogram of homologous segments in human and NHPs for SD_fusion_C (chr2:114,027,659-114,049,946), related to Figure 2. Red blocks represent homologous segments and green blocks represent pCht.

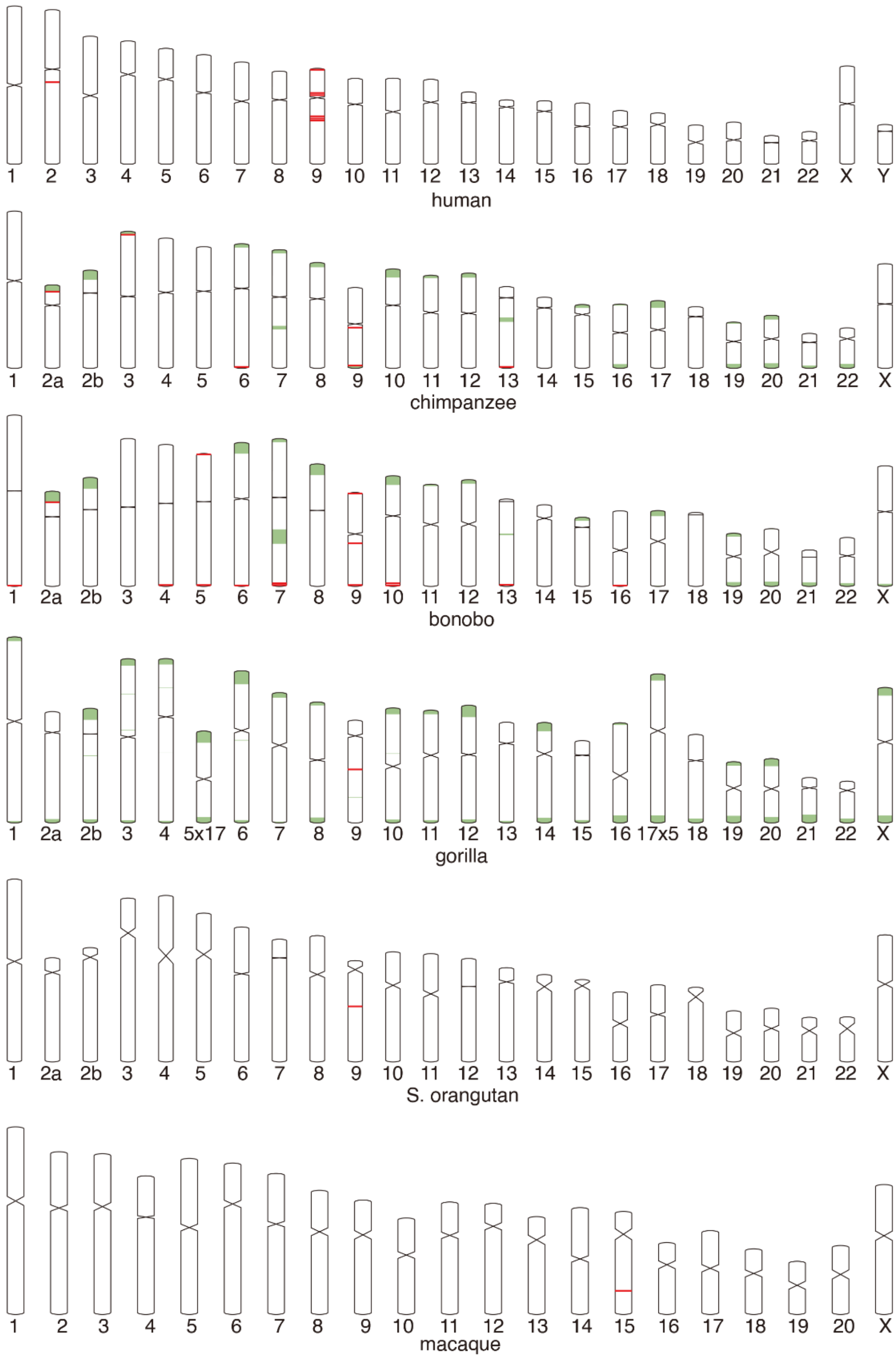

Figure S9. The ideogram of homologous segments in human and NHPs for the left flanking region 1 of the fusion site (chr2: 113,837,958-113,940,058), related to Figure 2. Red blocks represent homologous segments and green blocks represent pCht.

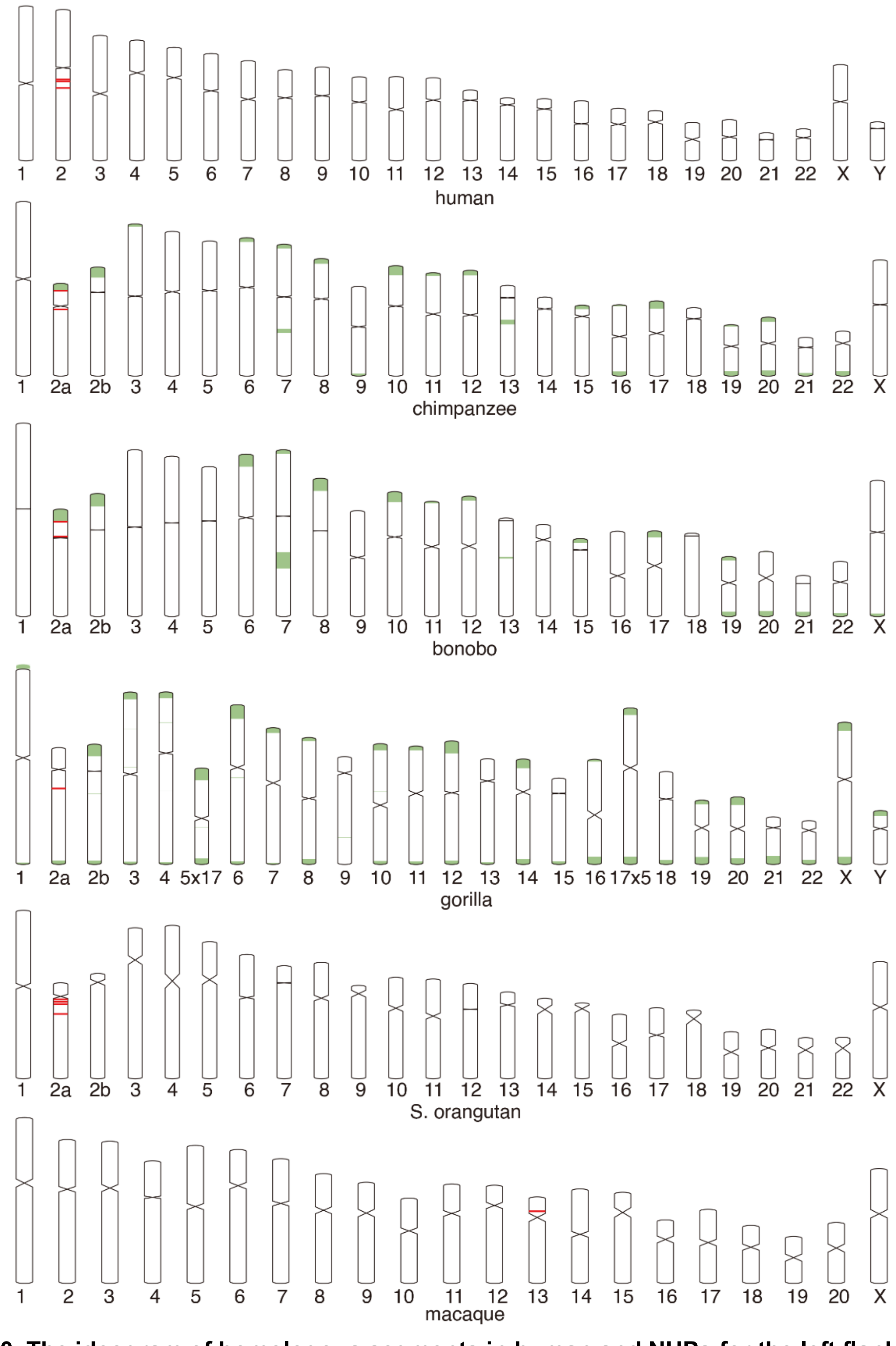

Figure S10. The ideogram of homologous segments in human and NHPs for the left flanking region 2 of the fusion site (chr2: 113,710,520-113,837,957), related to Figure 2. Red blocks represent homologous segments and green blocks represent pCht.

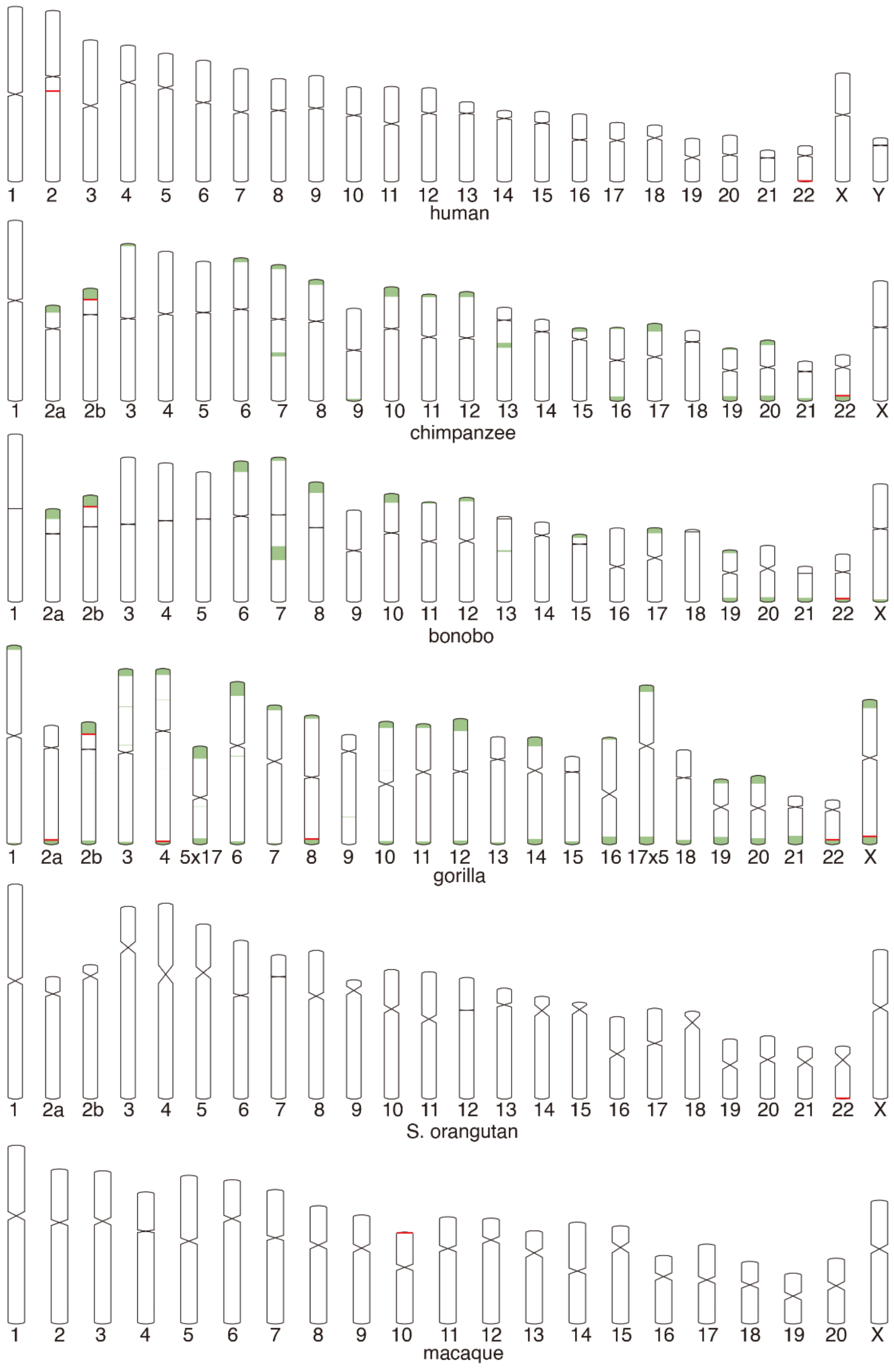

Figure S11. The ideogram of homologous segments in human and NHPs for the right flanking region 1 of the fusion site (chr2: 113,710,520-113,862,218), related to Figure 2. Red blocks represent homologous segments and green blocks represent pCht.

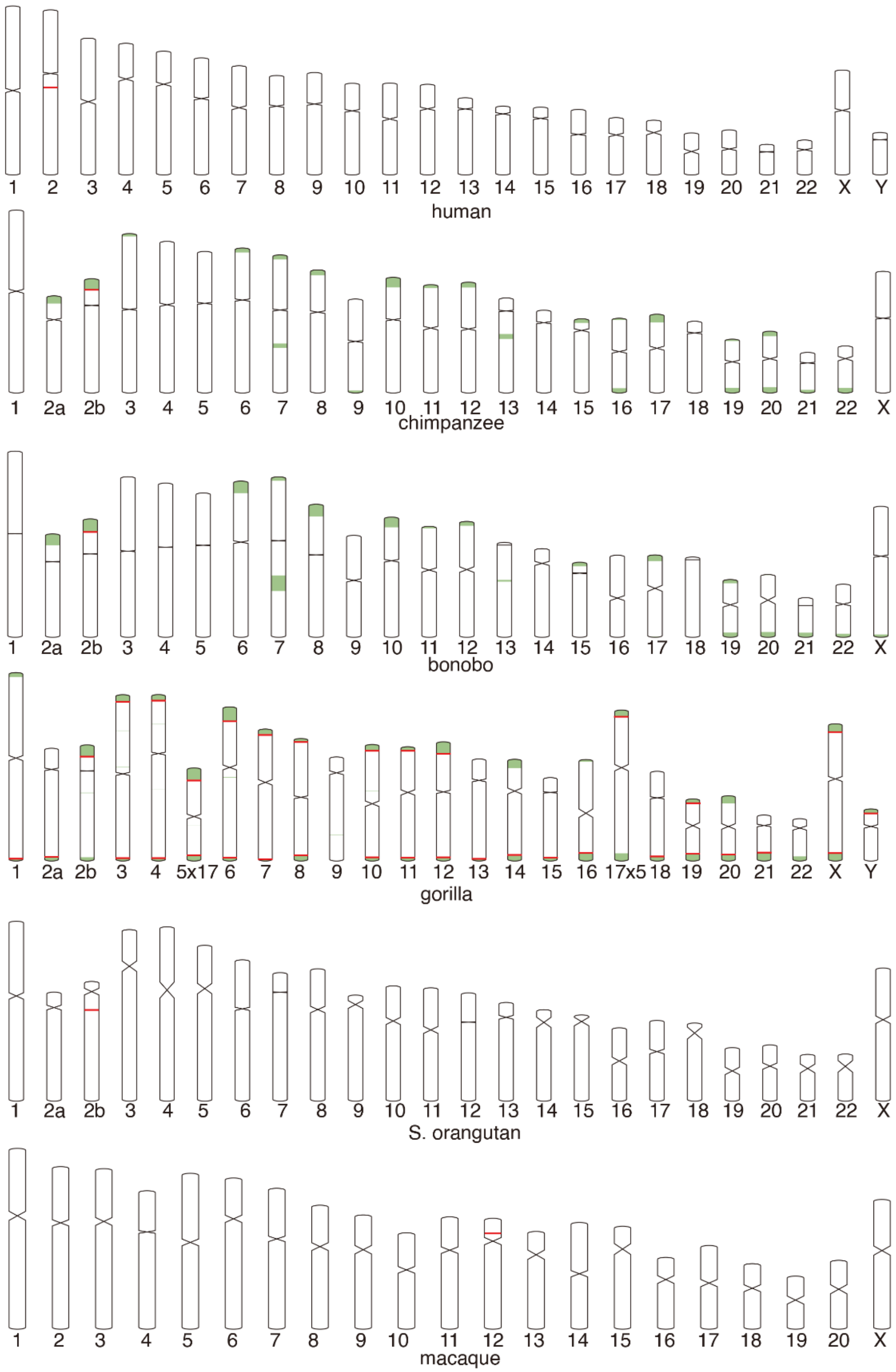

Figure S12. The ideogram of homologous segments in human and NHPs for the right flanking region 2 of the fusion site (chr2: 114,096,902-114,165,116), related to Figure 2. Red blocks represent homologous segments and green blocks represent pCht.

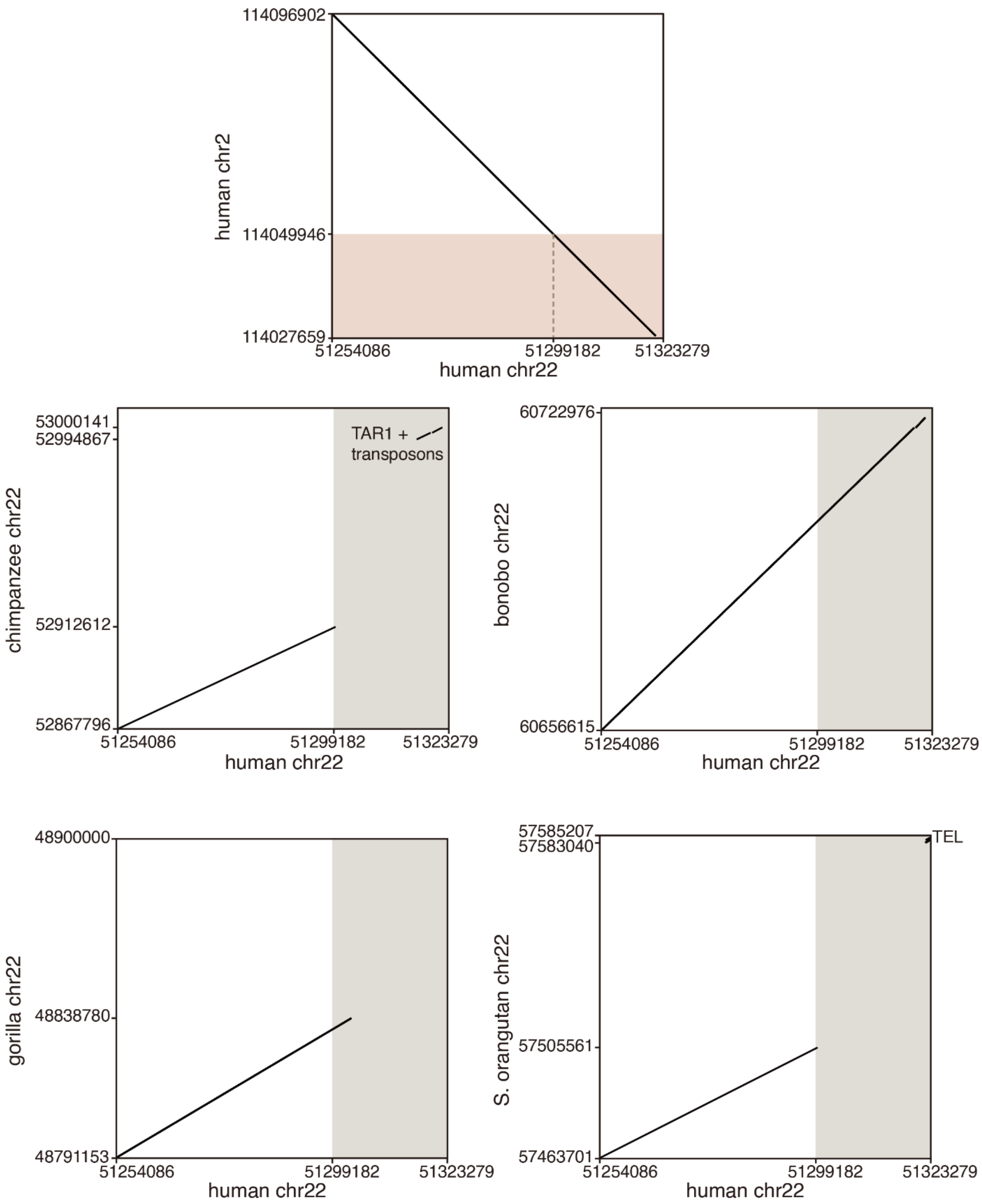

Figure S13. The identity dot plots of SDs among human SD_fusion_C and its flanking region, human chr22 segment, and NHP chr22 segment, related to Figure 2. The dot plot on the top shows the syntenic comparison between human SD_fusion_C (amber block) with its flanking region and human chr22 segment. The other dot plots show the human chr22 segment and its orthologous segments on each NHP.

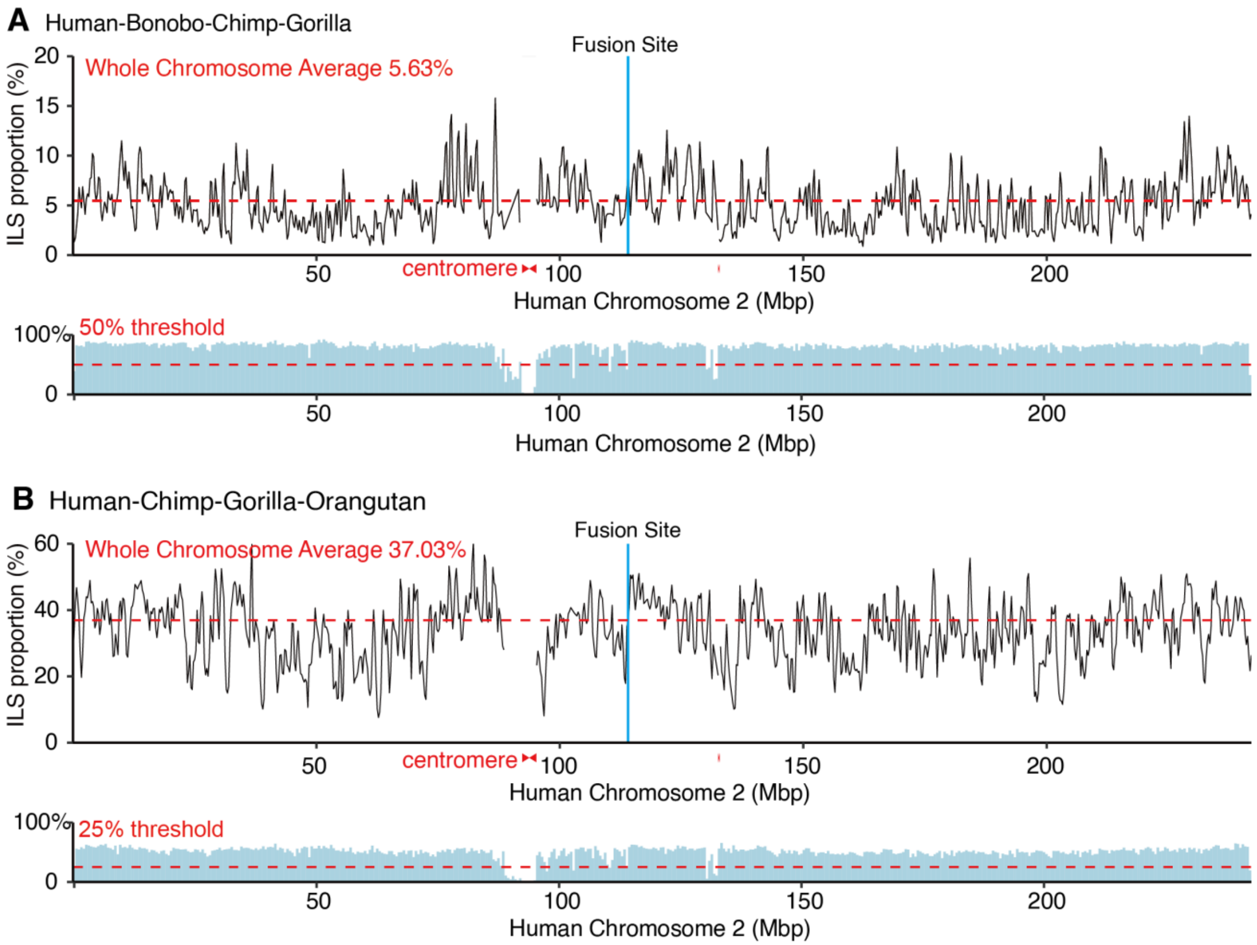

Figure S14. Incomplete lineage sorting (ILS) intensity on the whole chromosome 2, related to Figure 2. (A) ILS signal distribution for the entire chromosome 2 with human, chimpanzee, bonobo, and gorilla genomes. (B) ILS signal distribution for the entire chromosome 2 with human, chimpanzee, gorilla, and orangutan genomes. Phylogeny trees are constructed with 500 bp window sequences and the mean proportion of ILS is calculated with 500 kbp windows. For each panel, the upper plot shows the mean ILS proportion, and the bottom plot shows the effective data coverage in each window, indicating the mapping quality and reliability.

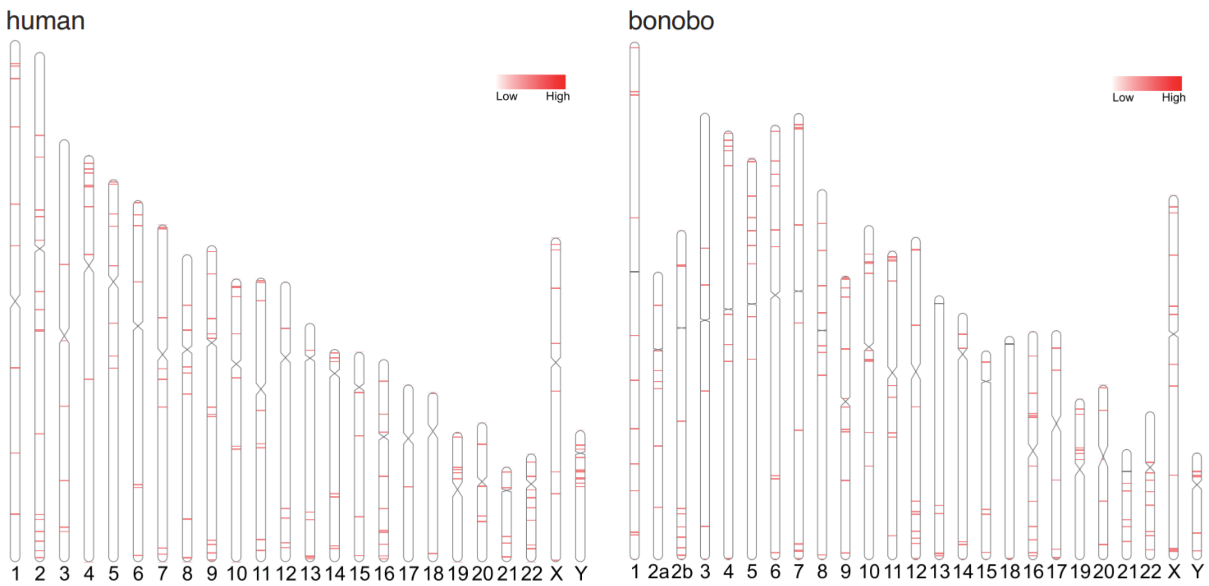

Figure S15. The telomeric sequences at the chromosomal interstitial sites in humans and bonobo, related to Figure 2. The red blocks represent the telomeric repeats.

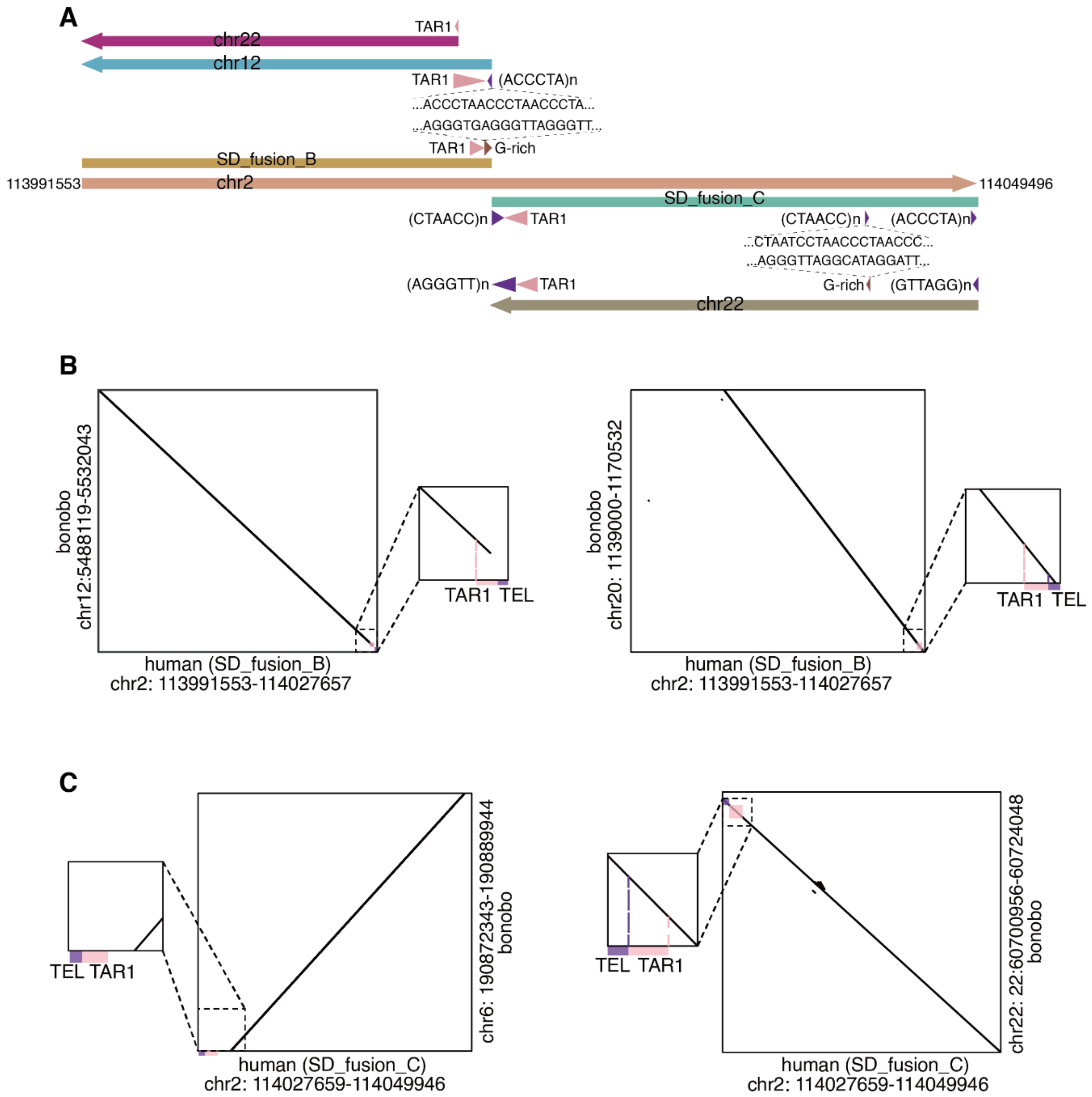

Figure S16. Telomeric sequences at the fusion site and associated SDs, related to Figure 2. (A) The human genomic segment (chr2:113,991,553-114,049,496) at the fusion site is shown in amber. SD from human chr12 is represented in blue and SD from human chr22 is depicted in tan. The tracks of repeat sequences were annotated by RepeatMasker. The G-rich regions show the similar telomeric sequences. Genomic regions in the bonobo that share homology with SD_fusion_B (B) and SD_fusion_C (C) contain similar telomeric sequences.

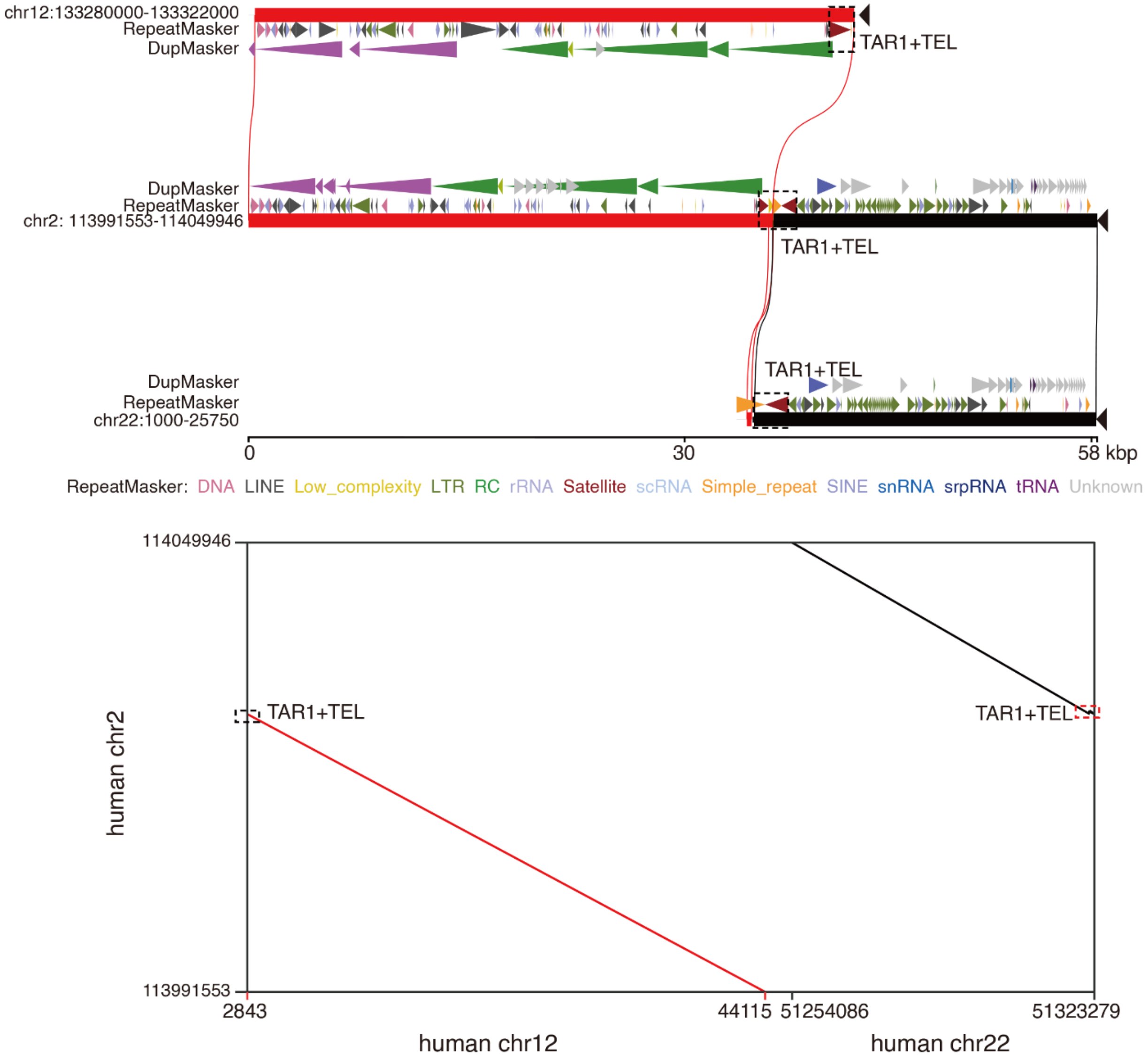

Figure S17. The orientation of telomeric-associated repeat (TAR1) and telomeric sequences at the fusion site in chr2 and SDs from chr12 and chr22, related to Figure 2. The synteny plot shows regions from chromosome 12 (red) and chromosome 22 (black) as reverse complement orientation, while the dot plot reveals that the TAR1 retains the same orientation as other SDs.

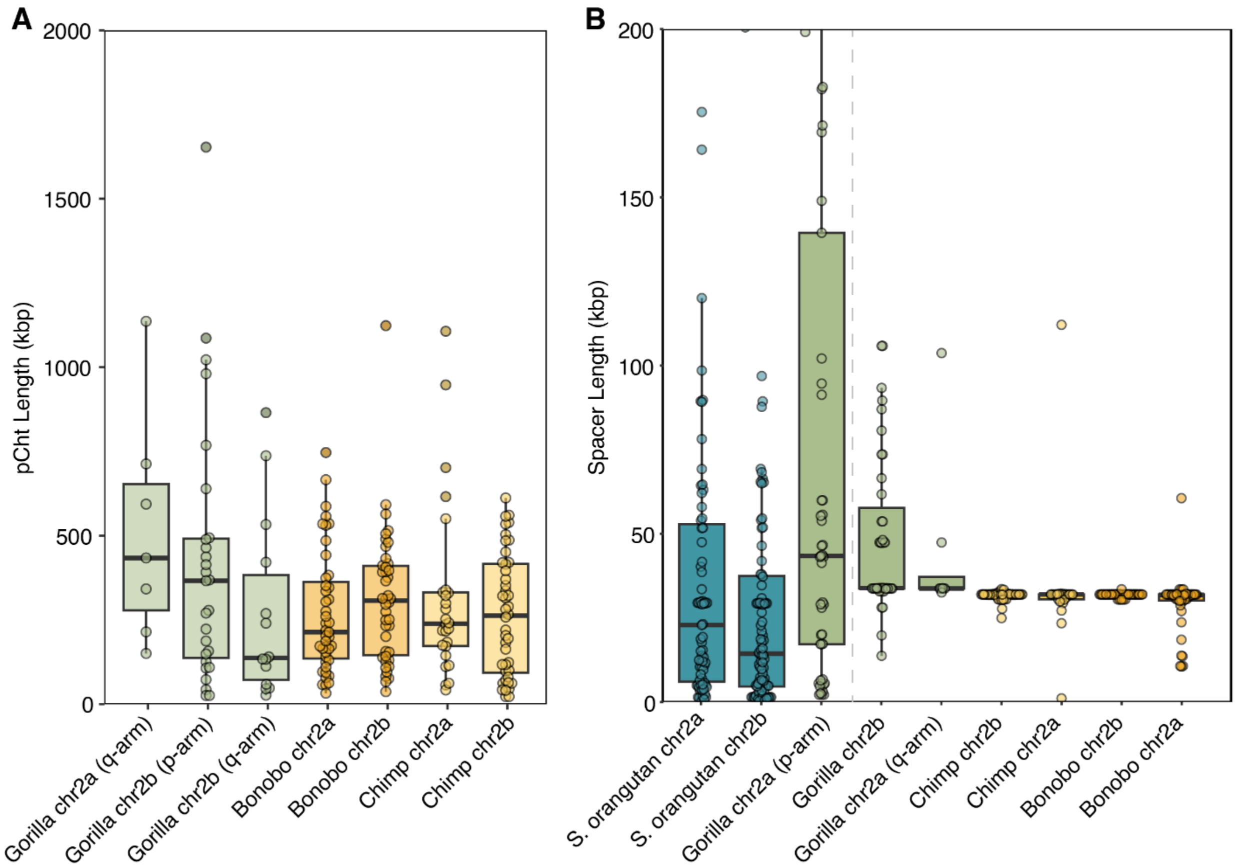

Figure S18. Distribution of pCht length and spacer length, related to Figure 3. (A) The pCht sequence lengths are determined by the distances between adjacent SD spacers. (B) The boxplots of spacer length highlight a normal distribution of lengths for *Pan* and gorilla spacers (excluding gorilla chr2a p-arm).

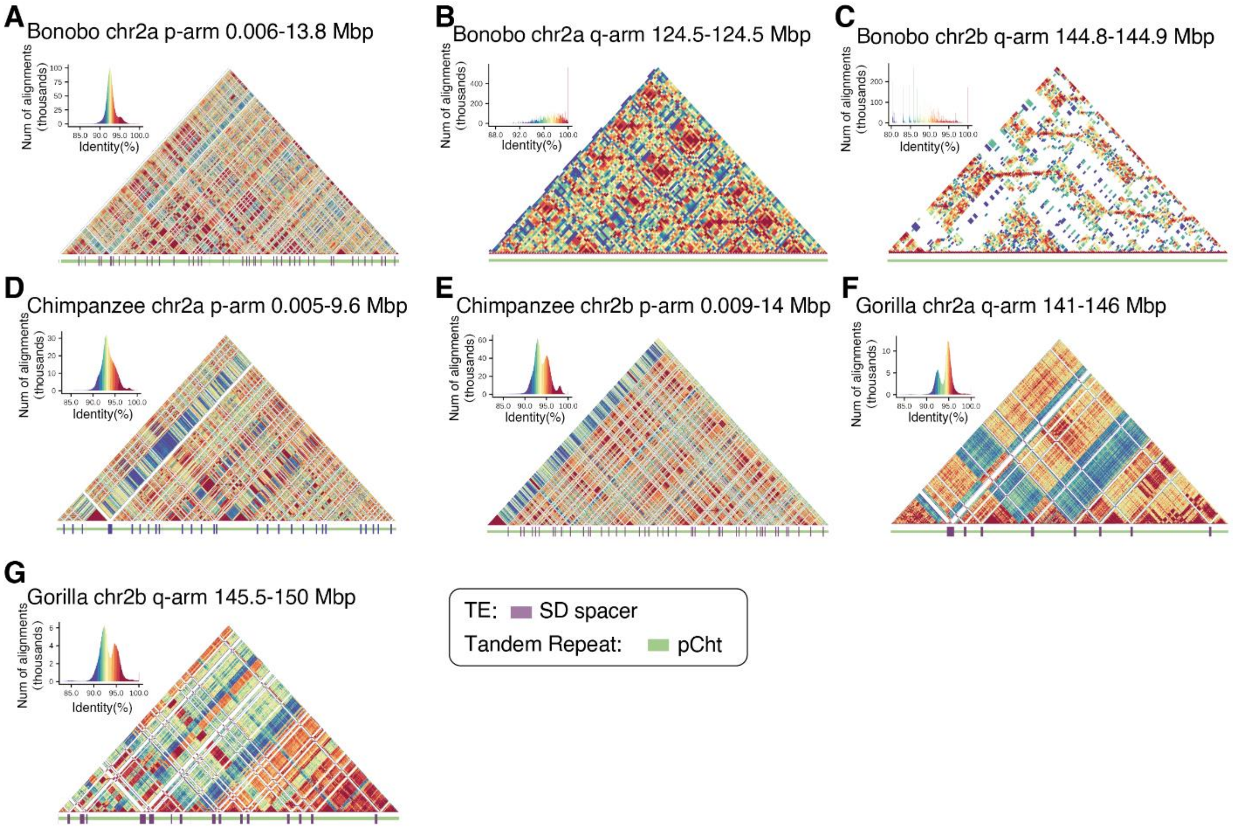

Figure S19. Identity heatmaps and spacer diagrams of subtelomeric repetitive regions in *Pan* and gorilla, related to Figure 3. The thicker and thinner rectangles below heatmaps stand for the transposable elements (TEs) track and tandem repeats (TRs) track, respectively. The species information and approximate coordinates of the other heatmaps and corresponding TE/TR tracks are at the top of the panels.

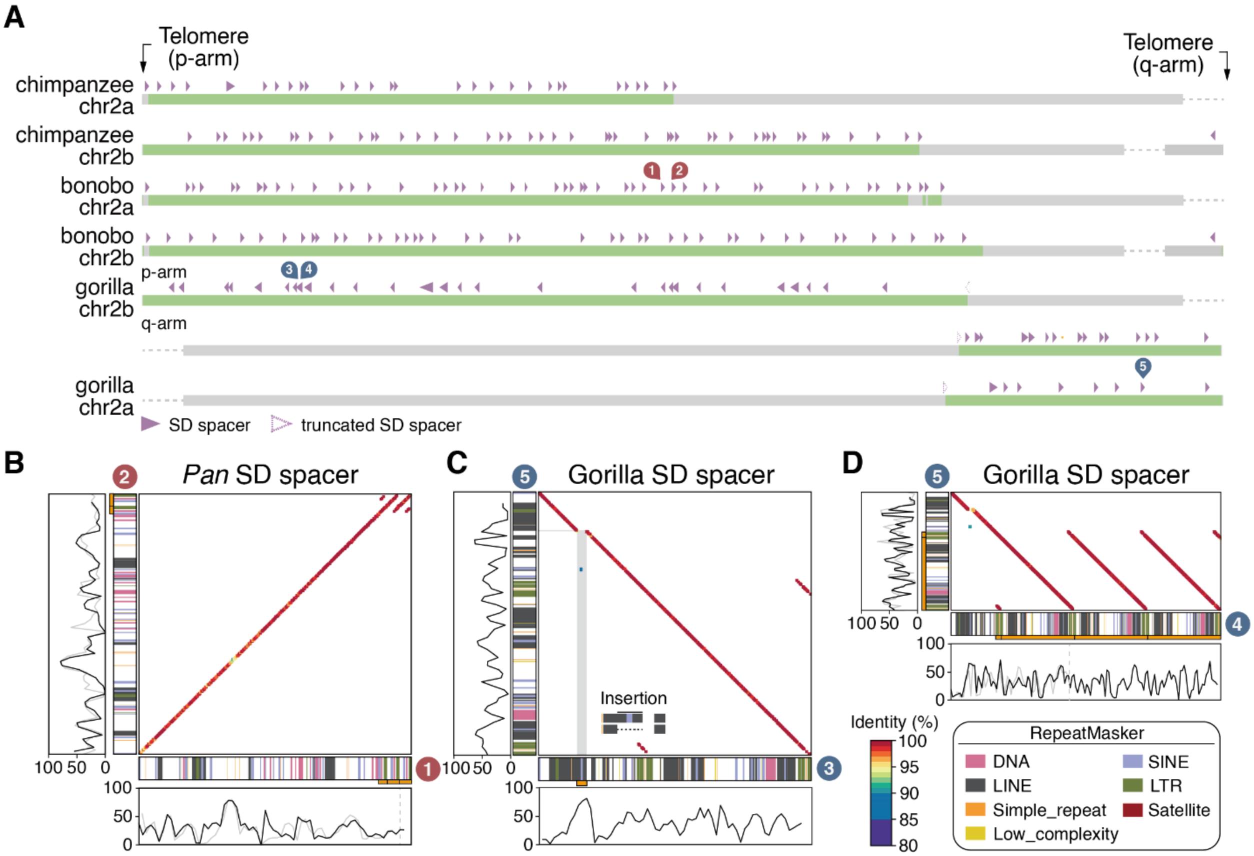

Figure S20. Diagram for spacer distribution and synteny among SD spacers, related to Figure 3. (A) The diagram illustrates the genomic structures of SD spacers (purple) within the pCht regions (light green) of gorilla and *Pan* species. Internal SVs and tandem duplications lead to the variable lengths of SD spacers in *Pan* (B) and gorilla (C-D). In panel (C), a 1.2 kbp LINE-SINE-LINE insertion is identified within an SD spacer in gorilla.

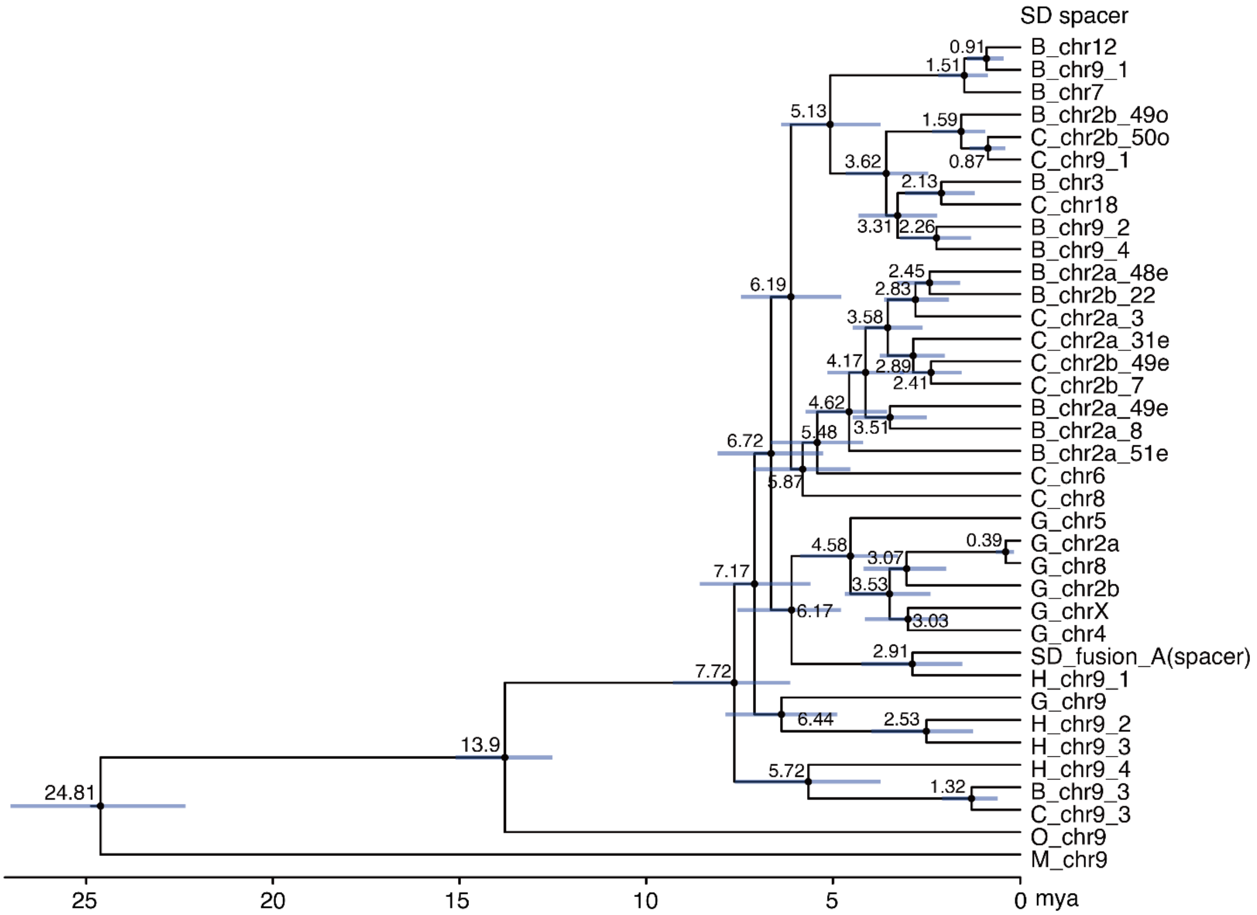

Figure S21. Phylogeny tree of *Pan* SD spacer, related to Figure 3. H, B, C, G, O, and M stand for human, bonobo, chimpanzee, gorilla, Sumatran orangutan, and macaque, respectively. Spacers on chr2 with ‘o’ and ‘e’ markers are out and end of subtelomeric repetitive regions, respectively. The other SD spacers on chr2 are in the subtelomeric repetitive regions.

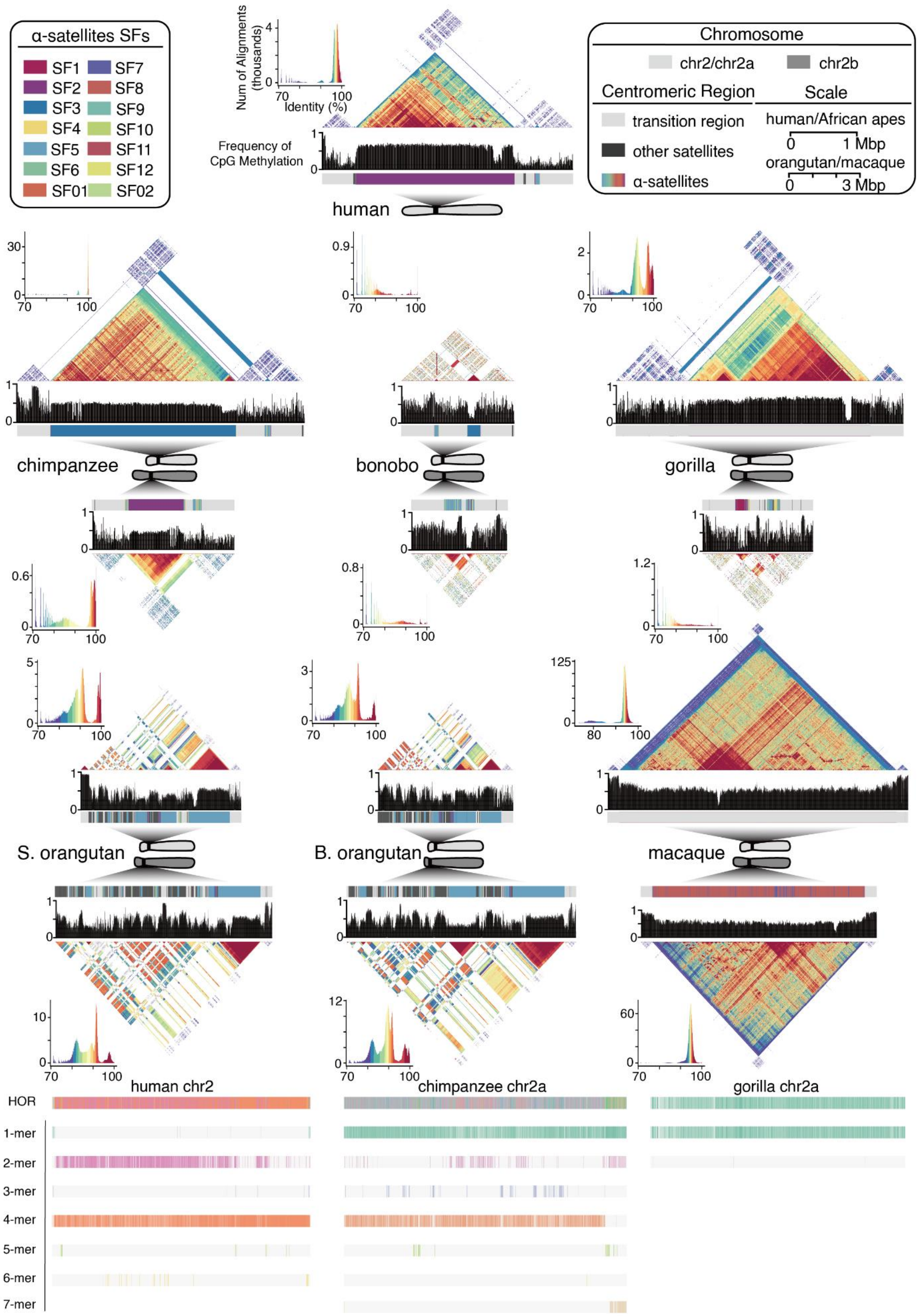

α-satellites SFs
SF1 SF7
SF2 SF8
SF3 SF9
SF4 SF10
SF5 SF11
SF6 SF12
SF01 SF02
Num of Alignments (thousands)
Identity (%)
Frequency of CpG Methylation
human
Chromosome
chr2/chr2a
chr2b
Centromeric Region
transition region
other satellites
α-satellites
Scale
human/African apes
0 1 Mbp
orangutan/macaque
0 3 Mbp
chimpanzee
bonobo
gorilla
S. orangutan
B. orangutan
macaque
human chr2
chimpanzee chr2a
gorilla chr2a
HOR
1-mer
2-mer
3-mer
4-mer
5-mer
6-mer
7-mer

Figure S22. Sequences and structures of centromeres in NHP chr2, related to Figure 4. Comparison of genomic structure, SF organization, and methylation landscape of α-satellite region flanking 500 kbp in human chr2, NHP chr2a, and NHP chr2b. Higher-order repeats (HOR) annotation of human chr2, chimpanzee chr2a and gorilla chr2a. Though the compositions of SFs in human and gorilla chr2a are similar (SF2, Figure 4A) but the organizations of monomers in HORs are different. Human chr2 centromeric array corresponds to a cluster of 2- and 4-monomers α-satellite HORs while gorilla chr2a centromeric array is rich of 1-monomer α-satellite HORs.

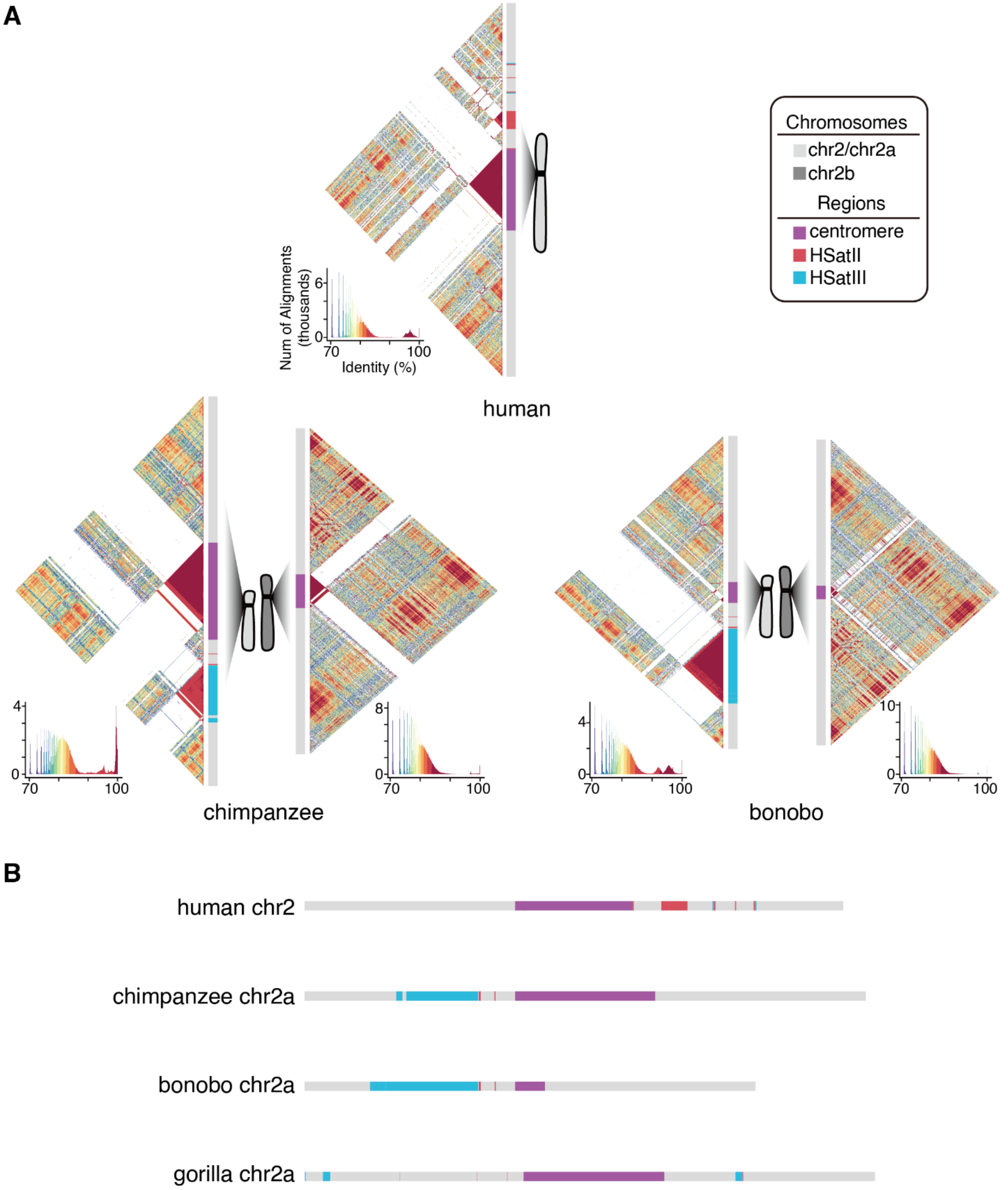

Figure S23. Structures of pericentromeric regions of human and *Pan*, related to Figure 4. (A) Genomic structure and specific satellite repeats in pericentromeric regions, including the α-satellite-rich regions and 5 Mbp on the p-arm and q-arm. Pink blocks represent the HSatII arrays and blue blocks represent the HSatIII arrays. (B) The satellite annotation of chromosome 2 in humans and chromosome 2a in chimpanzee, bonobo and gorilla.

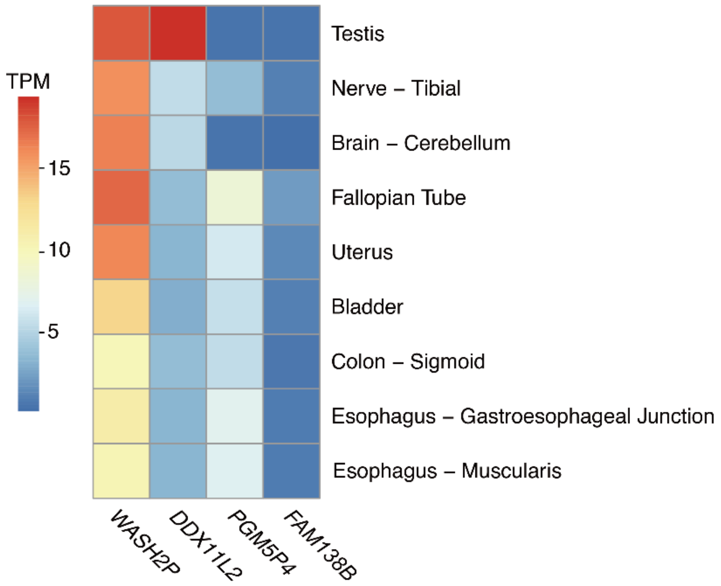

Figure S24. Expression levels of four noncoding genes in top expressed tissues, related to Figure 5. The median transcripts per million (TPM) from the GTEx database is used as the gene's expression level.

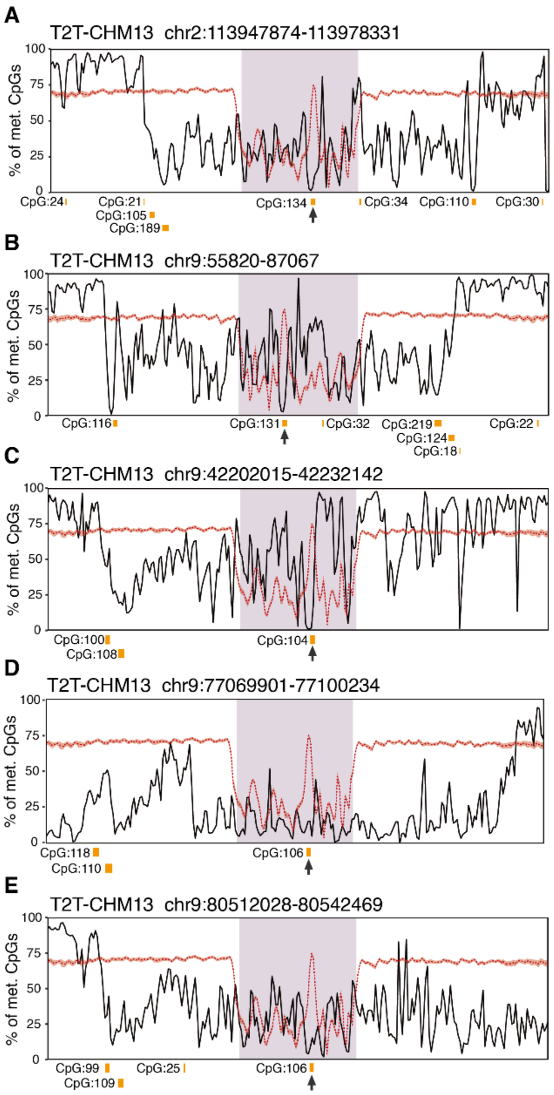

Figure S25. DNA methylation of CpG island in T2T-CHM13 *Pan* SD spacer, related to Figure 5. (A-E) The panels show the methylation status of the SD spacers in the human T2T-CHM13 genome as well as 50 kbp upstream/downstream regions. The violet shadow marks the SD spacer. The orange dashed line and black line show the average methylation level of the SD spacers in chimpanzee chr2b subtelomeric repetitive region (n=45) and methylation of this region, respectively. The lower panels under the methylation track show the CpG track from the UCSC Genome Browser and the CpG islands pointed by the arrows are significantly hypomethylated in human genome (excluding (D) chr9:77,069,901-77,100,234) but hypermethylated in *Pan* subtelomeric repetitive regions.

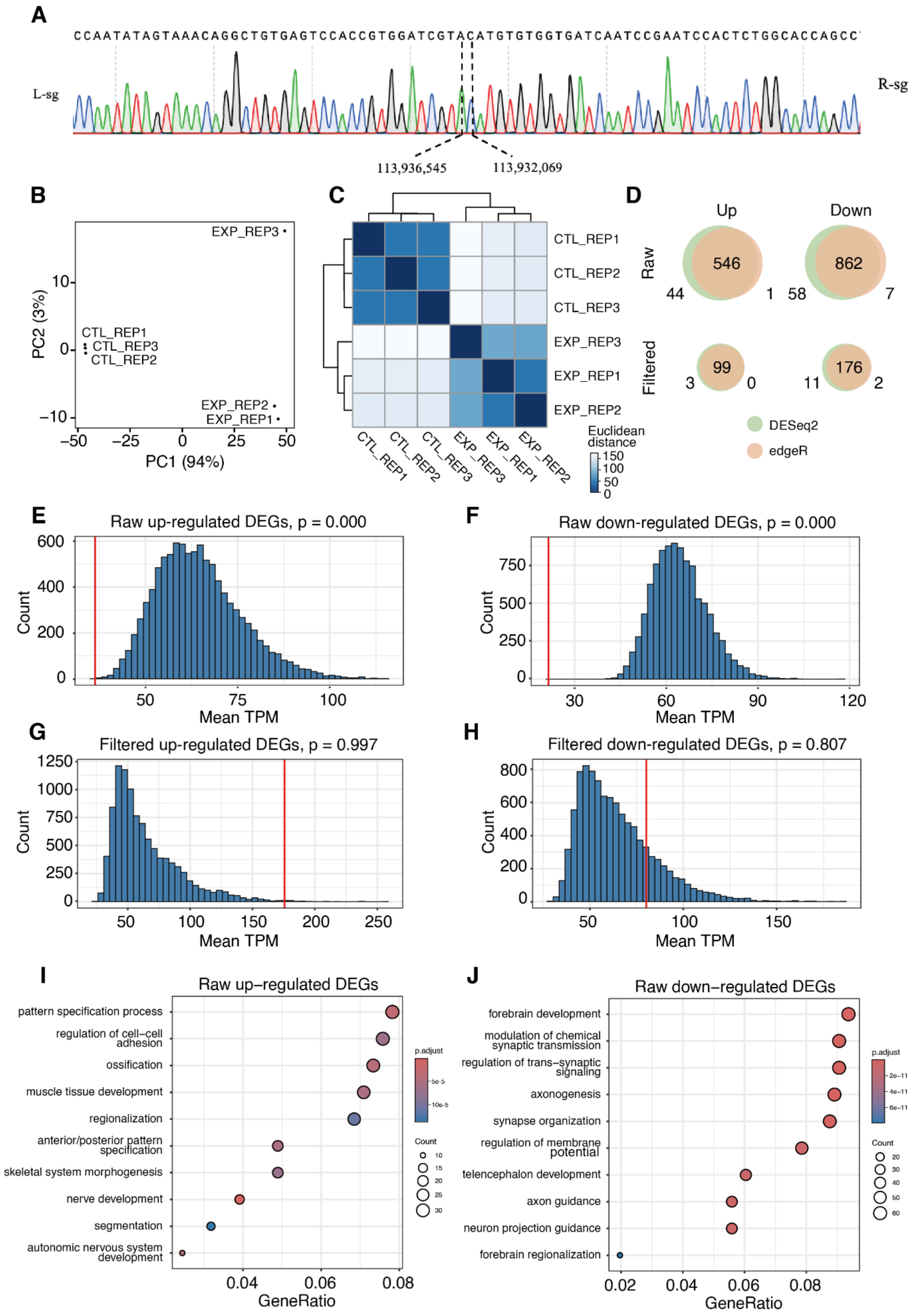
A
CCAATATAGTAAACAGGCTGTGAGTCCACCGTGGATCGTACATGTGTGGTGATCAATCCGAATCCACTCTGGCACCAGCC
L-sg
R-sg
113,936,545
113,932,069
B
PC1 (94%)
PC2 (3%)
EXP_REP3
CTL_REP1
CTL_REP3
CTL_REP2
EXP_REP2
EXP_REP1
C
CTL_REP1
CTL_REP2
CTL_REP3
EXP_REP3
EXP_REP1
EXP_REP2
Euclidean distance
D
Up
Down
Raw
Filtered
546
862
99
176
DESeq2
edgeR
E
Raw up-regulated DEGs, p = 0.000
F
Raw down-regulated DEGs, p = 0.000
G
Filtered up-regulated DEGs, p = 0.997
H
Filtered down-regulated DEGs, p = 0.807
Count
Mean TPM
I
Raw up−regulated DEGs
pattern specification process
regulation of cell−cell adhesion
ossification
muscle tissue development
regionalization
anterior/posterior pattern specification
skeletal system morphogenesis
nerve development
segmentation
autonomic nervous system development
GeneRatio
p.adjust
Count
J
Raw down−regulated DEGs
forebrain development
modulation of chemical synaptic transmission
regulation of trans−synaptic signaling
axonogenesis
synapse organization
regulation of membrane potential
telencephalon development
axon guidance
neuron projection guidance
forebrain regionalization
GeneRatio

Figure S26. RNA-seq analysis of CN1-derived neural progenitor cells, related to Figure 5. (A) Sanger sequencing result of the depleted fusion site. (B) Principal component analysis (PCA) of RNA-seq replicates using the top 500 most expressed genes shows strong replication within each condition. (C) The Euclidean distance matrix illustrates the consistency within groups and large differences between groups. (D) Comparison of raw and filtered differentially expressed genes (DEGs) identified by DESeq2 and edgeR. Venn diagrams show that while DESeq2 identified more DEGs than edgeR in both upregulated and downregulated categories, most DEGs were identified by both tools, demonstrating high consistency between two results. Permutation test on the mean transcript per million (TPM) of the raw upregulated (E) and downregulated DEGs (F) shows the low expression level of identified DEGs. Permutation test on the mean TPM of the filtered upregulated (G) and downregulated DEGs (H) indicates that there is no clear evidence of low expression for the filtered DEGs. GO enrichment analysis of raw upregulated genes (I) and downregulated genes (J) reveals that upregulated genes are associated with the pattern specification process, while downregulated genes are linked to neuronal development and organization process. The results before filtering and after filtering are highly consistent.

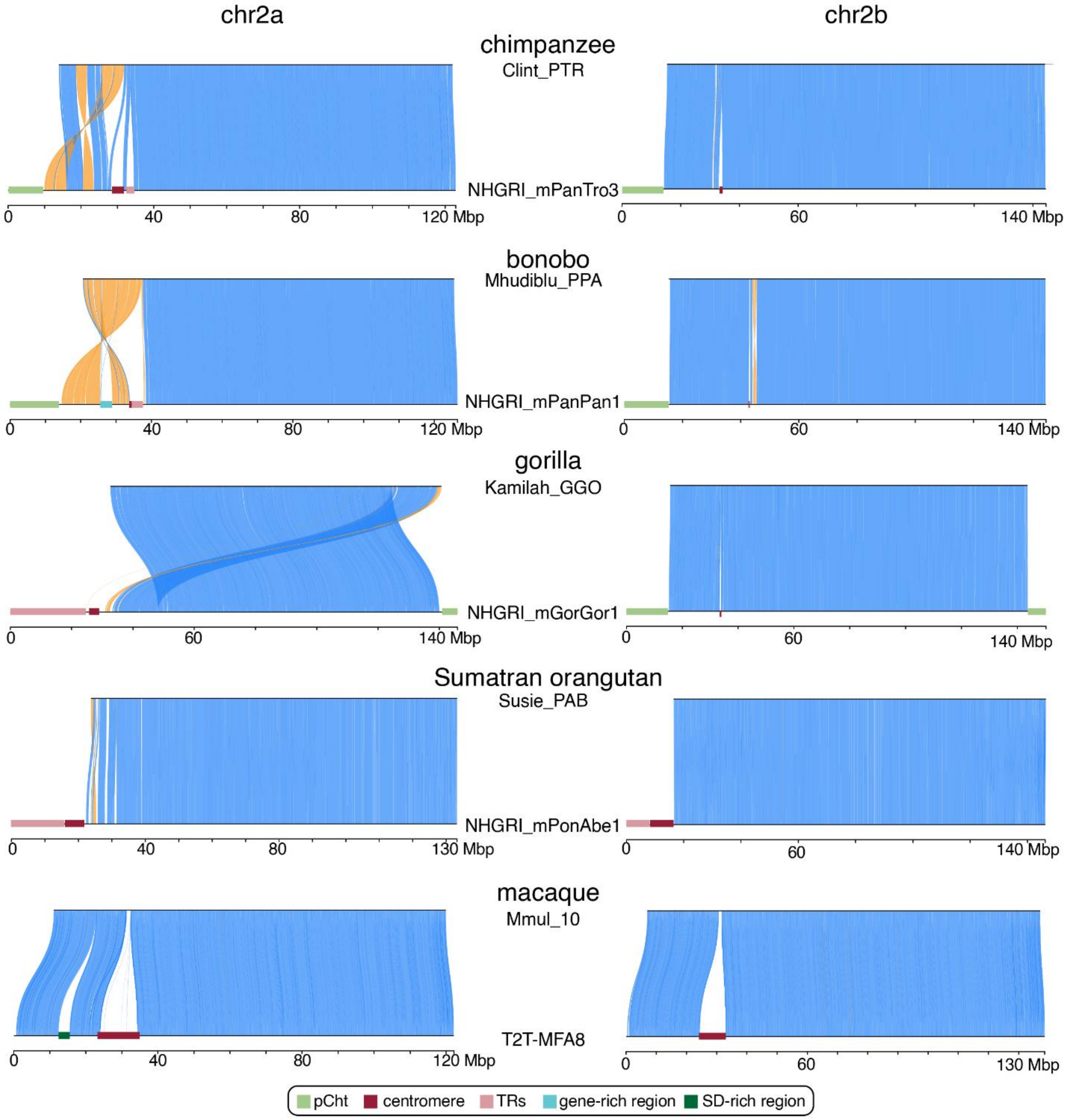

Figure S27. The chr2a/chr2b syntenic comparisons between previous genomes and T2T genomes in NHPs, related to Figure 6. The blue blocks show the syntenic alignments while the yellow blocks represent the inverted alignments. The red and pink blocks represent the previous unresolved regions. In addition, we provided more detailed analysis of the subtelomeric regions, including the SD_fusion_A/B/C on NHP genomes. We found 63 out of 74 SDs homologous to the human fusion site in T2T NHP genomes were not resolved in previous NHP genome assemblies (Table S14).

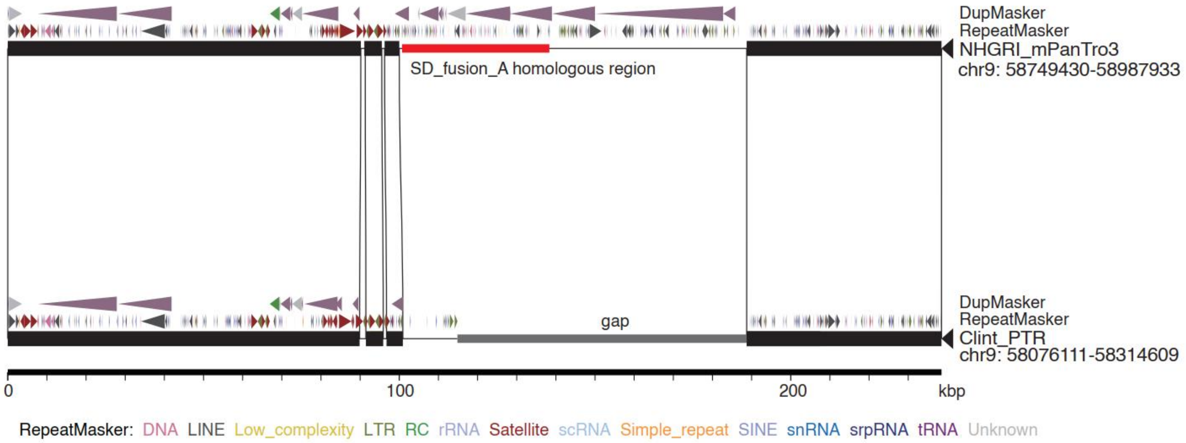

Figure S28. The comparison of a region containing SD_fusion_A in chromosome 9 between chimpanzee T2T genome and previous genome, related to Figure 6. The red block represents the SD_fusion_A homologous region in the chimpanzee T2T genome, while it wasn't assembled in previous genome. The gray block represents the gap in this region (scaffolded by N).

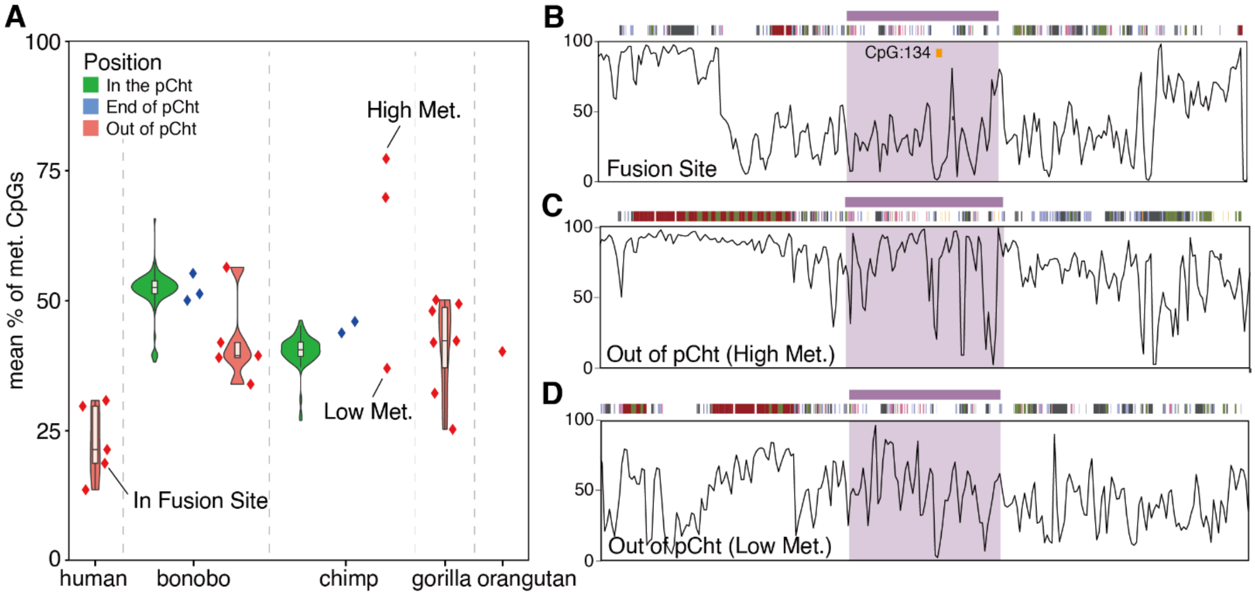

Figure S29. DNA methylation levels of SD spacers in different positions in human and nonhuman primates, related to Figure 6. (A) DNA methylation (Met.) levels of SD spacers in different positions in human and great apes. The methylation profiles of SD spacers in the fusion site (B), hypermethylated SD spacer out of subtelomeric repetitive region (C), and hypomethylated SD spacer out of subtelomeric repetitive region (D) are depicted.

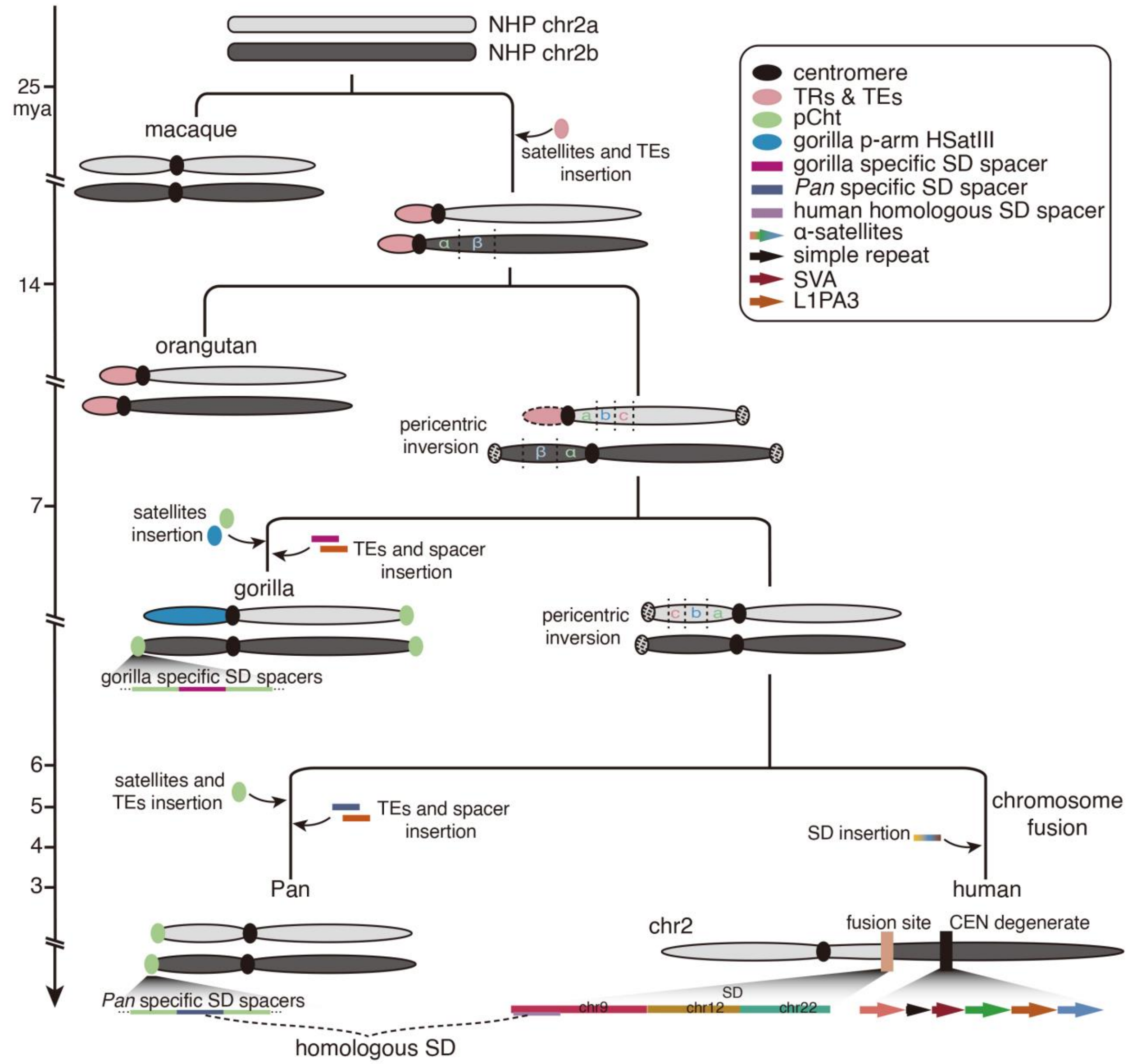

Figure S30. Model for chromosome 2 evolution, related to Figure 6. The NHP chr2a (light grey) and chr2b (dark grey) represent the karyotypes of each primate lineage and their reconstructed common ancestors. In orangutans, lineage-specific tandem repeats (TRs) and transposable elements (TEs) (pink) formed acrocentric chr2a and chr2b. A pericentric inversion on chr2b of the human–*Pan*–gorilla common ancestor (α, β vs. β, α) and specific TRs (blue for HSatIII, green for pCht) and gorilla-specific SD spacers turnover lead to the gorilla-specific karyotype. A subsequent inversion (a,b,c vs. c,b,a) in the human–*Pan* ancestor created an "unstable" chromosome structure. In the *Pan* lineage, specific TRs (green for pCht) and *Pan*-specific SD spacers result in *Pan*-specific subtelomeric repetitive structures of chr2a and chr2b. While human-specific SD insertions (pink for chr9 SD pairs, yellow for chr12 SD pairs, and green for chr22 SD pairs) were involved in the human chr2 fusion event. The SD spacers in *Pan* and the SD_fusion_A at the human fusion site have highly similar sequences, originating from a genomic region in the human–*Pan*–gorilla common ancestor. The three retained α-satellites are also shown with colorful arrows. The uncertain subtelomeric repetitive caps are shown with grey ovals with dashed lines.
