# Supplementary material for: Incomplete lineage sorting of segmental duplications defines the human chromosome 2 fusion site early during African great ape speciation
Source: Cell Genom. 2025 Dec 2;6(1):101079. doi: 10.1016/j.xgen.2025.101079 (PMC12926204; doi:10.1016/j.xgen.2025.101079)
Supplement: Document S3. Article plus supplemental information [file mmc9.pdf]

Cell Genomics

Article

Incomplete lineage sorting of segmental duplications defines the human chromosome 2 fusion site early during African great ape speciation

Graphical abstract

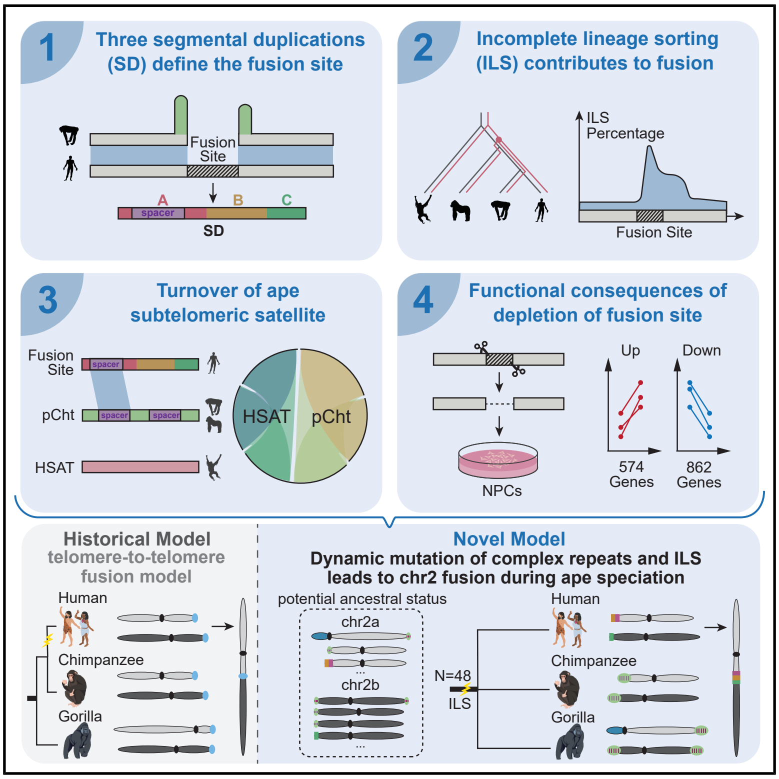

Authors

Zikun Yang, Lu Zhang, Xinrui Jiang, ..., Qiang Sun, Evan E. Eichler, Yafei Mao

Correspondence

qsun@ion.ac.cn (Q.S.),
ee3@uw.edu (E.E.E.),
yafmao@sjtu.edu.cn (Y.M.)

In brief

Yang et al. present the structure and evolution and investigate the functional consequences of human chromosome 2 fusion, suggesting incomplete lineage sorting of complex regions during great ape speciation.

Highlights

- Human chr2 fusion involved segmental duplications, inversions, and repeat turnover
- The fusion event is driven by ILS and occurred ~5–7 million years ago
- The fusion was formed by SDs and subtelomeric repeats rather than telomeric sequences
- Fusion site depletion alters gene expression in human neural progenitors

Yang et al., 2026, Cell Genomics 6, 101079
January 14, 2026 © 2025 The Author(s). Published by Elsevier Inc.
https://doi.org/10.1016/j.xgen.2025.101079

CellPress

Cell Genomics

CellPress
OPEN ACCESS

Article

Incomplete lineage sorting of segmental duplications defines the human chromosome 2 fusion site early during African great ape speciation

Zikun Yang,[1,2,3,18] Lu Zhang,[4,5,6,7,18] Xinrui Jiang,[1,18] Xiangyu Yang,[1,18] Kaiyue Ma,[1] DongAhn Yoo,[8] Yong Lu,[4] Shilong Zhang,[1,3] Jieyi Chen,[1] Yanhong Nie,[4] Xinyan Bian,[4] Junmin Han,[1] Lianting Fu,[1,3] Juan Zhang,[1] Mario Ventura,[9] Guojie Zhang,[3,10,11] Qiang Sun,[4,12,13,*] Evan E. Eichler,[8,14,*] and Yafei Mao[1,3,15,16,17,19,*]

[1]Bio-X Institutes, Key Laboratory for the Genetics of Developmental and Neuropsychiatric Disorders, Ministry of Education, Shanghai Jiao Tong University, Shanghai, China
[2]Zhiyuan College, Shanghai Jiao Tong University, Shanghai, China
[3]Center for Genomic Research, International Institutes of Medicine, Fourth Affiliated Hospital, Zhejiang University, Yiwu, Zhejiang, China
[4]Institute of Neuroscience, Center for Excellence in Brain Science & Intelligence Technology, Chinese Academy of Sciences, Shanghai, China
[5]School of Life Science and Technology, ShanghaiTech University, Shanghai, China
[6]Lingang Laboratory, Shanghai 200031, China
[7]Shanghai Center for Brain Science and Brain Inspired Intelligence Technology, Shanghai, China
[8]Department of Genome Sciences, University of Washington School of Medicine, Seattle, WA, USA
[9]Department of Biosciences, Biotechnology and Environment, University of Bari Aldo Moro, Bari, Italy
[10]Center of Evolutionary & Organismal Biology, and Women's Hospital at Zhejiang University School of Medicine, Zhejiang University, Hangzhou, Zhejiang, China
[11]University School of Medicine, Zhejiang University, Hangzhou, Zhejiang, China
[12]Key Laboratory of Genetic Evolution & Animal Models, Chinese Academy of Sciences, Kunming, China
[13]University of Chinese Academy of Sciences, Beijing, China
[14]Howard Hughes Medical Institute, University of Washington, Seattle, WA, USA
[15]Shanghai Jiao Tong University Chongqing Research Institute, Chongqing, China
[16]Shanghai Key Laboratory of Embryo Original Diseases, International Peace Maternity and Child Health Hospital, School of Medicine, Shanghai Jiao Tong University, Shanghai 200030, China
[17]Center for Comparative Biomedicine, Ministry of Education Key Laboratory of Systems Biomedicine, State Key Laboratory of Medical Genomics, Institute of Systems Biomedicine, Shanghai Jiao Tong University, Shanghai, China
[18]These authors contributed equally
[19]Lead contact
*Correspondence: qsun@ion.ac.cn (Q.S.), ee3@uw.edu (E.E.E.), yafmao@sjtu.edu.cn (Y.M.)
https://doi.org/10.1016/j.xgen.2025.101079

SUMMARY

All great apes differ karyotypically from humans due to the fusion of chromosomes 2a and 2b, resulting in human chromosome 2. Here, we show that the fusion was associated with multiple pericentric inversions, segmental duplications (SDs), and the turnover of subterminal repetitive DNA. We characterized the fusion site at the single-base-pair resolution and identified three distinct SDs that originated more than 5 million years ago. These three distinct SDs were differentially distributed among African great apes as a result of incomplete lineage sorting (ILS) and lineage-specific duplication. One of these SDs shares homology to a hypomethylated SD spacer sequence present in the subterminal heterochromatin of *Pan* but is completely absent subtelomerically in both humans and orangutans. CRISPR-Cas9-mediated depletion of the fusion site in human neural progenitor cells alters the expression of genes, indicating a potential regulatory consequence to this human-specific karyotypic change. Overall, this study offers insights into how complex regions subject to ILS may contribute to speciation.

INTRODUCTION

Karyotype evolution is a critical aspect of evolutionary biology because it has been associated with speciation, adaptation, and disease.[1–7] In 1935, Painter and Stone first observed that chromosome fusion could lead to speciation in flies.[5] As the field advanced, numerous instances of chromosome fusion have been documented in plant and animal speciation.[1,3,4,6,8–10]

Several molecular mechanisms have been proposed as key drivers of chromosome fusion in speciation and oncogenesis, including telomere-telomere, telomere-centromere, tandem repeat, segmental duplication (SD), and retrotransposon expansions and fusions.[11–15]

Recent advances in genome editing and synthetic biology have provided deeper insights into the role of chromosome fusion in evolution.[16–18] Boeke and colleagues, for example,

© 2025 The Author(s). Published by Elsevier Inc.
This is an open access article under the CC BY license (http://creativecommons.org/licenses/by/4.0/).

demonstrated that chromosome fusion could induce reproductive isolation in yeast, highlighting the potential of these genomic changes to influence speciation.[19,20] Additionally, studies on chromosome engineering in mice reveal significant chromatin conformation alterations in the chromosome fusion regions, further emphasizing the impact of karyotype evolution on genetic and phenotypic diversity.[21,22]

Human chromosome 2 (chr2) was formed from the fusion of nonhuman primate (NHP) chr2a and chr2b,[23–25] representing arguably the most significant karyotypic difference between humans and NHPs. Ijdo et al. were the first to identify the fusion site at human chromosome 2q13-2q14 using cytogenetic banding approaches.[24] Subsequent studies uncovered dispersed SDs associated with this fusion site.[26,27] These analyses relied on partial genome assemblies generated using short-read technologies or Sanger sequencing of bacterial artificial chromosome (BAC) clones, often resulting in incomplete genetic information at the fusion site.[26–29] Consequently, the structure, evolutionary history, and function of the fusion site have remained only partially understood.

Available genomic assemblies and fluorescence *in situ* hybridization (FISH) experiments have shown an abundance of satellite sequences (pCht sequences) in the subtelomeric repetitive regions of NHP chr2a and chr2b, whereas such sequences are absent in humans.[27,30,31] During the fusion of human chr2, one of the centromeres in the fused chromosome becomes inactive and degraded, with independent transposable element (TE) retrotransposition events occurring at the degenerate site.[32] In addition, there has been considerable debate regarding the timing of the chr2 fusion event. Based on SD and SVA (SINE-VNTR-Alu; SINE, short interspersed nuclear element; VNTR, variable number tandem repeat) divergence, the fusion event was estimated to have occurred early in human evolution, 5–7 and 2.5–4.5 million years ago (mya), respectively.[27,33] However, a recent study, utilizing clustered substitution statistics, proposed a much more recent origin of approximately 0.9 mya.[34]

The complex repetitive structure at the fusion site, subtelomeric repetitive regions, and the inactive centromere have made it challenging to reconstruct the evolutionary history and potential functional consequences of the fusion event. In the absence of complete genome assemblies, most SDs and satellite arrays remain unresolved,[35,36] limiting our ability to examine their evolutionary dynamics. Previous studies using long-read sequencing have shown that SDs can undergo rapid lineage-specific expansion,[37–39] yet no comprehensive analysis has been performed at the human chr2 fusion site. Meanwhile, pCht satellite sequences have been fully characterized in a recent ape genome study, but their detailed relationship with the fusion event remains unclear. Here, we leverage the complete sequences of great apes[38,40] and a macaque[39] (as an outgroup of the great apes) to revisit the structure and evolutionary history of human chr2. We aim to (1) characterize the fusion site at single-base-pair resolution, (2) study this in the context of epigenetic and structural changes occurring at the subtelomeric repetitive regions and degenerate centromere site, (3) use these data to create a model for human chr2 evolution, and (4) examine the potential functional consequences by creating fusion-site-depletion cell lines.

RESULTS

Comparative sequence analysis of the human chr2 fusion site

To characterize the syntenic regions and structural changes associated with the fusion site in primates, we performed a comparative analysis of 10 finished nonhuman great ape chromosomes[38] (including chimpanzee, bonobo, gorilla, Sumatran orangutan, and Bornean orangutan) and the finished macaque genome[39] to human chr2 (Figure 1A; STAR Methods). We identified numerous non-syntenic segments (76–154 regions ≥10 kbp in length) between humans and NHPs, accounting for 9.86 Mbp (macaque) and up to 57.5 Mbp (gorilla) of unalignable chromosomal sequence per species. Most of this sequence corresponded to various classes of repetitive DNA (Figure 1A; Table S1), including satellite repeats and tandem and interspersed SDs. In particular, among the nonhuman African great apes, both chr2a and chr2b are acrocentric, with the presence of subterminal heterochromatic caps (5–19.6 Mbp) demarcating the ends of the chromosomes, and are composed of 32 bp AT-rich satellite DNA (pCht) and SD spacer regions.[27,31,38,41] In addition, there are multiple pericentric and paracentric evolutionary inversions distinguishing the ape lineages from each other,[42] several of which share homology with the SD flanking regions of the fusion site (Figures 1B and 2A). One pericentric inversion occurred in the common ancestor of African great apes after divergence from orangutans (chr2b), while another occurred after the *Pan*-gorilla split (chr2a)[42,43] (Figure 1A). Furthermore, we identified the truncated gene *CBWD2*, caused by the truncated SD at the flanking of the chr2a pericentric inversion breakpoint, while a truncated SD but complete gene *FOXD4L1* was found near the chr2b pericentric inversion breakpoint (Figure S1).[28,29] Furthermore, comparative analysis reveals that the pericentric inversions in nonhuman great apes have shaped the gene architecture near the ancestral telomeres. Specifically, the chr2a pericentric inversion disrupted *CBWD2*, resulting in a truncated gene in the *Pan* lineage, whereas the chr2b inversion left *FOXD4L1* intact (Figure S1). These ancestral gene configurations are preserved in humans and flank the chr2 fusion site.

Evolutionary reconstruction of the ancestral fusion site

To precisely identify the fusion site at single-base-pair resolution and decompose its substructure, we further compared human chr2 with *Pan* chr2a and chr2b to characterize the ~109 kbp fusion site in the human telomere-to-telomere (T2T)-CHM13v2.0 genome assembly (2q14.1, chr2:113940058–114049496) (Figures 1B and S2). Leveraging data from 47 human genomes from the Human Pangenome Reference Consortium (HPRC),[44] we confirmed that there is a complete absence of large structural variants within this fusion site in 94 sequence-resolved human haplotypes, suggesting a fixed structural haplotype for the entire fusion site (Figure S3). Neither nucleotide diversity (π) nor Tajima's D values show significant reductions at the fusion site in African or non-African populations, relative to the chr2 average (Figure S4), consistent with the locus evolving neutrally.

To gain deeper insight into the evolutionary history of the ~109 kbp fusion site, we separated this region into three distinct,

CellPress
OPEN ACCESS

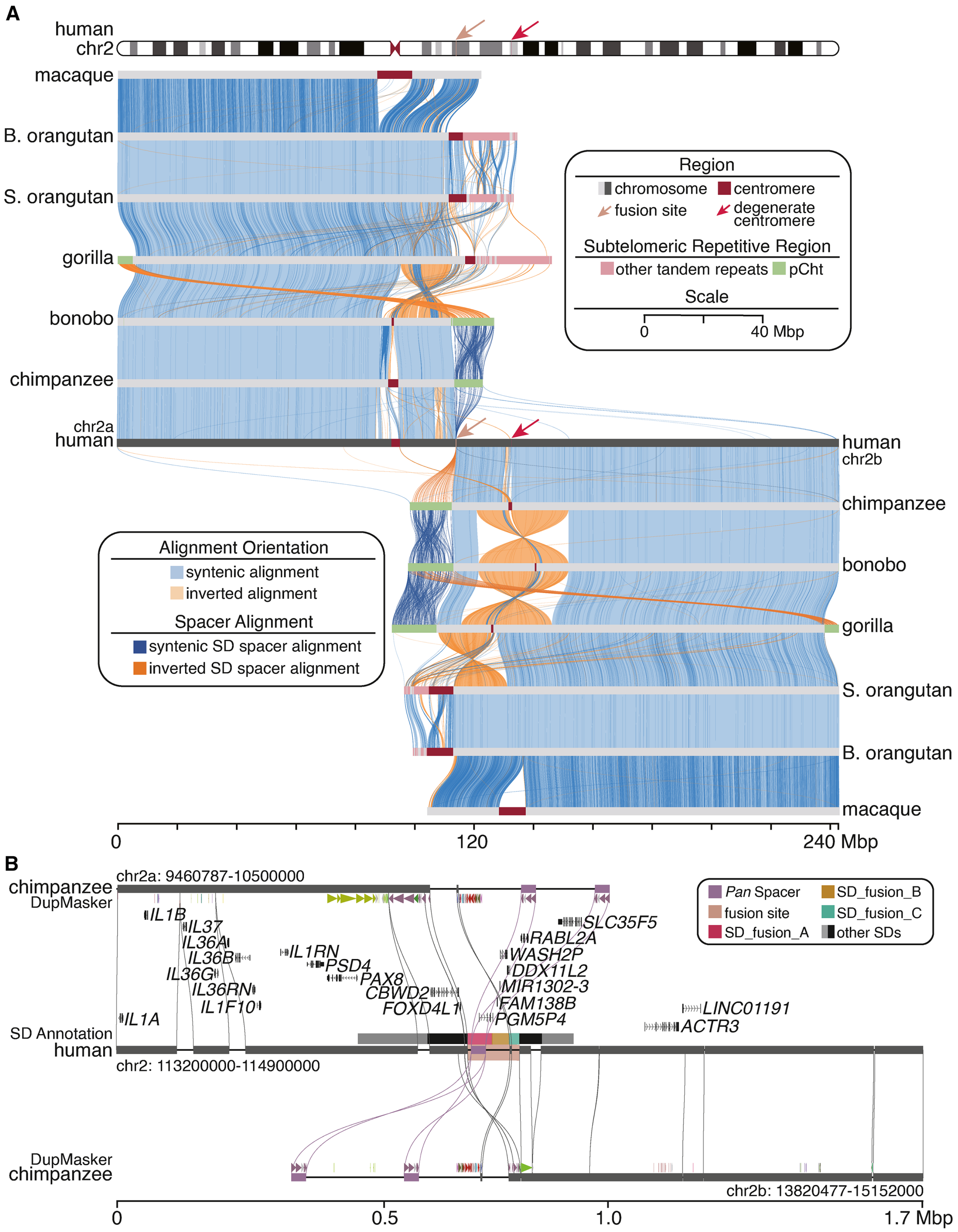

Figure 1. The comparative sequencing analysis of primate chromosome 2

(A) The syntenic comparison of chromosome 2 (chr2) highlights the extent of primate chr2 evolutionary rearrangements. Syntenic regions conserved in order (blue) are contrasted with evolutionary inversions (orange) and non-syntenic regions (breaks) corresponding to α-satellite (dark brown), pCht subterminal satellite (green), and other satellite (pink) DNA. B. orangutan and S. orangutan represent the Bornean orangutan and the Sumatran orangutan, respectively.

(B) High-resolution analysis of the human fusion site (chr2:113940058–114049496, colored in amber) shows the non-syntenic breakpoint region in the context of annotated human protein-coding genes and segmental duplications (SDs). *Pan* SD spacers (purple blocks) in chimpanzee pCht show the homology of the partial region of the human fusion site.

See also Figures S1 and S2 and Table S1.

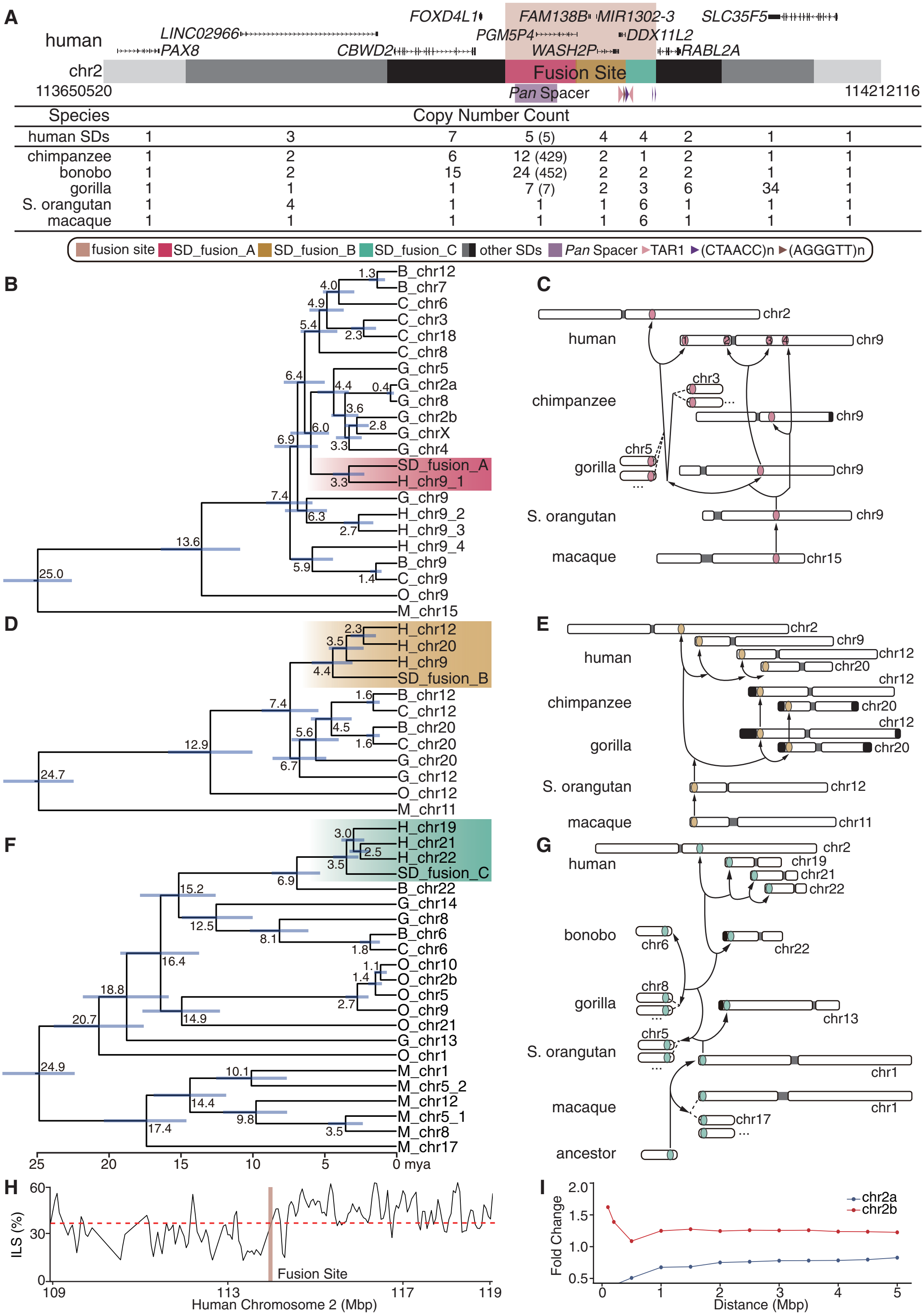

Figure 2. The genomic structure and evolutionary history of SDs at the human fusion site

(A) Human chromosome 2 (chr2) fusion site and SD organization. A human genomic segment (chr2:113650520–114212116) with gene annotations shows the region where chr2a and chr2b fused (chr2:113940058–114049496, amber). The region consists of a large (∼455 kbp) duplication block made up of seven SDs

(legend continued on next page)

high-identity SDs (≥98% identity and ≥20 kbp in length) within the larger ~455 kbp duplication block (seven independent SD blocks) (Figures 1B, 2A, and S5–12; Tables S2A and S2B). In humans, these SDs share homology with sequences corresponding to chr9 (SD_fusion_A, 50 kbp), chr12/20 (SD_fusion_B, 36 kbp), and chr22 (SD_fusion_C, 22 kbp) (Figure 2A; Table S2A). Among African apes, each SD is highly variable in copy number, showing evidence of shared ancestral locations as well as lineage-specific duplications (Table S2A). Using macaque and orangutan as outgroups, we reconstructed the evolutionary history of each SD, estimating the divergence time point from each SD to its nearest nonhuman great ape neighbor (Figures 2B–2G; STAR Methods).

SD_fusion_A (50 kbp) is present as a single copy in macaques and orangutans and corresponds to a partial duplication of *PGM5*[29] (14 exons of an ancestral gene), a gene involved in carbohydrate metabolism, originating from African great ape ancestral chr9 (phylogenetic group IX) (Figures 2B and 2C). This SD began to duplicate in an interspersed configuration in the common ancestral lineage of African great apes (~7.4 mya), with the locus expanding to the highest copy number in chimpanzees and bonobos, where it defines the SD spacer region demarcating large blocks of pCht on chr2a and chr2b and other chromosomes (chr3, chr5–chr13, chr15–chr22, and chrX in chimpanzee; each chromosome in the bonobo genome) (Figures 2B, 2C, and S6). Of note, the SD_fusion_A (chr2) and other human copies share a monophyletic origin (~3.3 mya), most closely related to gorilla copies ~6 mya (95% confidence interval [CI]: 4.8–7.4 mya) instead of chimpanzee or bonobo—a pattern consistent with incomplete lineage sorting (ILS).

Similarly, SD_fusion_B is a 36 kbp segment corresponding to the partially truncated *WASH2*[45] (11 exons of an ancestral gene that potentially regulates actin cytoskeleton dynamics), *FAM138B* (3 exons of an ancestral gene of unknown function), and *DDX11* (3 exons of an ancestral gene implicated in DNA metabolism). The segment exists as a single copy in both macaques and orangutans and originated from a locus mapping to ancestral African great ape chr12 (phylogenetic group XII) (Figures 2D, 2E, and S7). All human copies mapping to human chr2, chr20, chr12, and chr9 show a monophyletic origin (~4.4 mya, 95% CI: 3.1–5.8 mya), suggesting human-specific duplications or interlocus gene conversion events.[46] With respect to nonhuman African great apes, however, this clade diverges from a distinct monophyletic clade that includes chimpanzee, gorilla, and bonobo (~7.4 mya, 95% CI: 5.4–9.3 mya) (Figure 2D). This topology is once again consistent with ILS.

In contrast to SD_fusion_A and SD_fusion_B, SD_fusion_C (the most distal, 22 kbp) shows evidence of independent duplication in all primates, including macaques and orangutans, with the exception of chimpanzees, where it exists as a single copy on chimpanzee chr6 (Figure S8). All human SDs show a monophyletic origin (~3.5 mya, 95% CI: 2.7–4.3 mya) but show a deep coalescence with another genomic segment present only on bonobo chr22 (phylogenetic group XXII), dating back to ~6.9 mya (95% CI: 5.3–8.6 mya) (Figures 2F and 2G). Notably, SD_fusion_C and its flanking region correspond to a larger SD that aligns exclusively between the human chr2 SD_fusion_C site and bonobo chr22 but not with any other NHPs (Figure S13). Thus, SD_fusion_C and its flanking region likely represent an ancestral genomic structure in the latest common ancestor (LCA) of African great apes, which was subsequently sorted in the human and bonobo lineages.

ILS refers to the random segregation of ancestral alleles into descendant lineages, a process that typically arises when the ancestral population size is large.[47–49] ILS results in gene tree discordance and deep coalescence. Previous studies have speculated that ILS may be involved in evolutionary divergence or speciation, although direct evidence and the underlying mechanisms remain to be fully explored.[50,51] Next, we investigated whether ILS also occurs in the regions flanking the fusion site. To this end, we extended the ILS analysis to the 5 Mbp mapping proximally (chr2a) and distally (chr2b) to the fusion site in humans (STAR Methods) using a 500 bp windowed approach[37] (Table S2C). We observe a sharp transition in the proportion of ILS windows at the site of the chr2 fusion. Specifically, we find that the proportion of ILS rises to 50.69% (a 1.39-fold excess compared to the chr2 average) as we approach the fusion site distally (chr2b side). In contrast, the proximal portion appears depleted for ILS segments (Figures 2H, 2I, and S14; Table S2D). There is, thus, a polarized pattern of ILS with maxima and minima occurring on either side of the fusion site.

The refined analysis of telomeric sequences at the fusion site

Previous investigations identified inverted telomeric sequences (TTAGGG/CCCTAA) at the fusion site, suggesting a T2T fusion.[24] However, whether these sequences are remnants of the ancestral telomeres from chr2 fusion or simply telomeric-like repeats interspersed within ancestral non-telomeric regions remains unresolved. First, we confirmed the presence of interstitial telomeric sequences (CCCTAA, chr2:114027659–114028207) at SD_fusion_C and its corresponding SD on human chr22 (TTAGGG, chr22:51254086–51323279) and similar

with variable copy numbers in each ape genome. The three SDs at the fusion site are designated as SD_fusion_A, SD_fusion_B, and SD_fusion_C. The table indicates the copy number of the full-length homologous segments for each primate genome (with brackets indicating the SD_fusion_A copy number of the derived sequence in the subterminal heterochromatic caps of chimpanzees and bonobo; see Table S2A for the detailed alignments at the fusion site).

(B, D, and F) Phylogenetic trees based on a (B) 50 kbp (SD_fusion_A), (D) 36 kbp (SD_fusion_B), and (F) 22 kbp (SD_fusion_C) multiple sequence alignment show that these regions have been subject to incomplete lineage sorting (ILS).

(C, E, and G) The chromosome schematic depicts the genomic locations of SD_fusion_A (C), SD_fusion_B (E), and SD_fusion_C (G) in each primate.

(H) Proportion of human-gorilla and *Pan*-gorilla tree topology in a 500 bp window of 5 Mbp flanking region near the fusion site. The red dotted line represents the mean value of the whole chr2.

(I) Fold change of the mean proportion of discordant topologies in the flanking region compared with the whole genome average. (Note that chromosome names in the great apes refer to the human homologous chromosome, also known as the phylogenetic group designation.)

See also Figures S3–S17 and Table S2.

sequences in bonobo chr22 (TGAGGG, chr22:60722979–60724048). The sequence GGGTTA was identified at SD_fusion_B (chr2: 114027333–114027658) and its corresponding SD on human chr12 (TAACCC, chr12:2843–3030) and bonobo chr20 (TAACCC, chr20:1139021–1139266) but not at its corresponding SD on human chr20 (Figures S15 and S16). These observations argue that these telomeric sequences were present on SD_fusion_B and SD_fusion_C prior to the fusion event. In addition, two telomeric-associated repeats (TAR1) flank the telomeric sequences of SD_fusion_B and SD_fusion_C.[26] Given the genomic structure of TAR1 and the telomeric sequences on these SDs (Figures S16 and S17), we propose that, in the ancestral configuration, each SD contained a TAR1 element and telomeric sequences, arranged in an inverted orientation on ancestral chr2a and chr2b. The end-to-end fusion of these two distinct SDs may have facilitated the fusion of ancestral chr2a and chr2b, leading to the vestigial presence of two TAR1 elements and the CTAACC and GGGTTA repeat motifs at the human fusion site.

African great ape subterminal satellite repeat expansion and human chr2 fusion

Reconstructing the evolutionary history of the fusion event has been challenging due to extensive SD and lineage-specific turnover of the subterminal heterochromatic caps in *Pan* and gorilla[38] (Table S3A). With the exception of the short arm of gorilla chr2a, the corresponding regions in both *Pan* and gorilla are composed of nearly continuous megabase-pair tracts of satellite DNA interspersed with SD spacers.[38] The satellite sequence is made of a tandem 32 bp repeat motif (pCht)[31] punctuated on average every 287 kbp in *Pan* and every 389 kbp in gorilla by an SD spacer (Figures 3A and S18–S20). The interrupting SD spacers are variable with a modal length of 32 kbp in the *Pan* lineage and 33.7 kbp in gorilla (Figure S18) and correspond to hypomethylated pockets flanked by the hypermethylated satellite DNA[38] (Figures 3A, S19, and S20). Although both the gorilla and chimpanzee SD spacers differ in sequence composition and the subterminal heterochromatic cap is largely thought to have evolved independently in both lineages,[27,38] the net effect is that the subterminal portions of both chimpanzee chr2a and chr2b share 23.6 Mbp of high-identity sequence homology involving both the SD spacers and pCht satellite DNA. In the case of the gorilla, the homology is restricted to the pCht satellite DNA for both arms of gorilla chr2b and the q-arm of gorilla chr2a. The organization of the p-arm of gorilla chr2a differs considerably and is much more similar to the organization found in orangutan, which is enriched in HSatIII-like repeat sequences (Figure 3B), which is consistent with a previous report.[52] It is classified as an acrocentric short arm lacking a nuclear organizing region.[38]

Importantly, the SD spacer that expanded in the subterminal heterochromatic caps in chimpanzee and bonobo shares 98.05% identity with the SD_fusion_A segment mapping at the fusion site on human chr2 (Figure 3C). All 429–452 SD spacers within the *Pan* pCht regions, including those at the ends of these chromosomal regions, are monophyletic in origin, estimated to have expanded approximately ~5.5 mya (95% CI: 4.2–6.8 mya). This subterminal SD spacer (32 kbp) in *Pan* is 18 kbp smaller than SD_fusion_A (50 kbp), where the shared LCA predates the human-*Pan*-gorilla divergence (Figures 2B and 2C). Comparing the phylogenies of the sequence unique to SD_fusion_A and the sequence shared with the subterminal heterochromatic cap SD spacers shows nearly coincident ILS topology (generalized Robinson-Foulds distance = 0.28, $p = 1.6 \times 10^{-4}$). This indicates that the SD spacers within *Pan* heterochromatic caps are derived from the duplicated sequence that gave rise to ancestral SD_fusion_A. Thus, the hyperexpansion of subterminal satellite DNA associated with subterminal heterochromatic caps in *Pan* and the chr2 fusion are linked genetically with two different evolutionary trajectories and karyotypic consequences in human and *Pan* (Figures 3C, 3D, and S21; Table S3B).

Centromere retention and degeneration in human chr2

An important consequence of the human chr2 fusion is the inactivation of the ancestral chr2b centromere from NHPs in the human lineage. Here, we refer to this site as Cen_decay (short for centromere degeneration; Figure 4A). Chr2a differs from chr2b by the presence of large tracts of HSatII arrays in humans and HSatIII arrays in *Pan* (Figures S22 and S23). Overall, the active human centromere in humans is more similar to that of the gorilla with respect to suprachromosomal family (SF) organization. Both humans and gorillas possess SF2, whereas chimpanzees and bonobos have SF3.

We compared the active chromosome centromere of chimpanzee chr2b with the structure of the vestigial centromere in humans. We identified three distinct α-satellite arrays (chr2:132644386–132685996) in humans[52] with homology to chimpanzee that had been interrupted by various simple repeats (CCTCTC) and retrotransposon elements (SVA and L1PA3) in the human lineage (Figure 4B). All three satellite arrays were derived from divergent monomeric α-satellite regions ancestral to human and chimpanzee rather than from higher-order repeats (HORs). Our analysis of 94 human genome assemblies[42] identifies five distinct structural haplotypes due primarily to length variation of the first two α-satellite arrays (Figures 4C and 4D; Table S4). A sequence comparison using unique *k*-mers from this region shows that ~97.5% (8,246/8,460) and ~96.7% (8,181/8,460) are also identified in Neanderthal and Denisovan genomes, respectively (STAR Methods), confirming that the centromeric degeneration occurred long before the divergence of modern and archaic humans.[32]

We also compared methylation patterns of the NHP chr2a and chr2b centromeres (Figure 4E). The human α-satellite arrays mapping to the degenerate site show significantly lower methylation levels when compared to typical NHP HORs (excluding centromere dip regions [CDRs], $p = 0.02$), with the exception of bonobo chr2b and gorilla chr2b. HORs in bonobo chr2b and gorilla chr2b are significantly hypomethylated when compared to other NHP HORs ($p = 1.92 \times 10^{-6}$), possibly due to the smaller size of these HORs (lengths: 71 kbp for bonobo chr2b and 106 kbp for gorilla chr2b).

Functional assessment of the fusion site by depletion

As the fusion site is nearly fixed in the human genome, we speculated that it may confer a functional advantage. To explore this possibility, we examined gene models and expression patterns

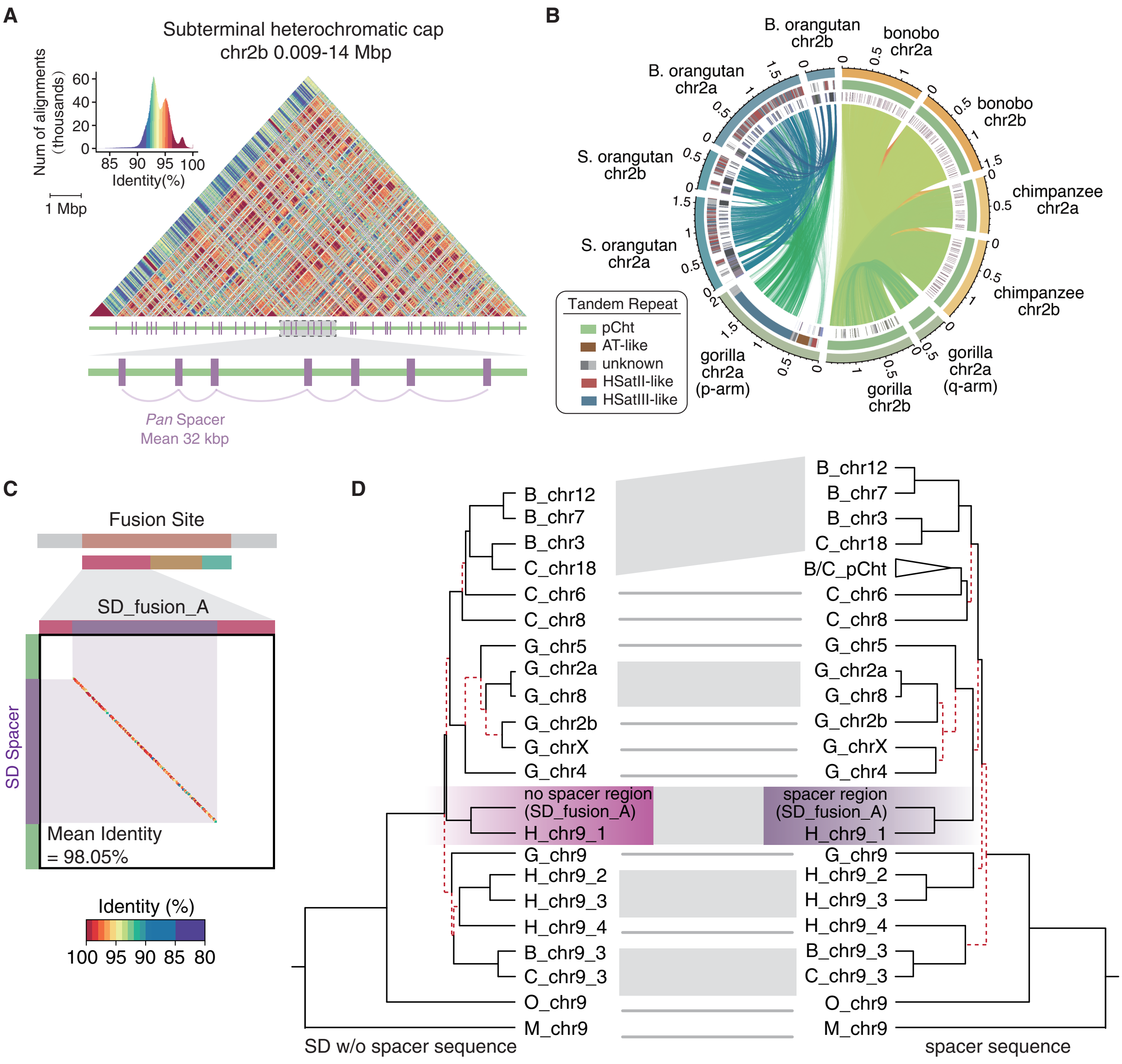

Figure 3. Turnover of subtelomeric repetitive regions in primates and evolutionary connection between SD spacers in *Pan* lineage and SD_fusion_A at the fusion site

(A) The identity heatmap of the subterminal heterochromatic cap for the p-arm of chimpanzee chr2b. SD spacer elements (purple) are annotated below and are flanked by large tracts of pCht satellite (green) (zoomed-in image shows the structure at higher resolution).

(B) Circos plot shows sequence identity of chr2a and chr2b among the apes, highlighting the turnover of satellite DNA. The three layers (outer to inner) represent the subtelomeric region, tandem repeat satellites, and transposon element annotations.

(C) Dot plot shows the synteny between a bonobo SD spacer within the heterochromatic cap vs. human SD_fusion_A segment. The sequence identity was calculated at a 100 bp resolution, with the dot color representing sequence identity ranging from 80% (purple) to 100% (red).

(D) Phylogenetic topology comparison shows nearly consistent ILS evolutionary pattern of SD_fusion_A (excluding spacer sequence) and SD spacer. The red lines indicate discordant tree topologies within each major clade between the two trees.

See also Figures S18–S21 and Table S3.

in this region and generated knockout (KO) cell lines to assess potential gene expression changes associated with the fusion site. Four putative noncoding genes/pseudogenes (*PGM5P4*, *FAM138B*, *WASH2P*, and *DDX11L2*) have been annotated at the site of the human chr2 fusion. According to GTEx short-read RNA sequencing (RNA-seq), these four genes/pseudogenes are expressed in testis, esophagus, fallopian tube, and cerebellum tissues[53] (Figure S24). Further, both *PGM5P4* and *WASH2P*

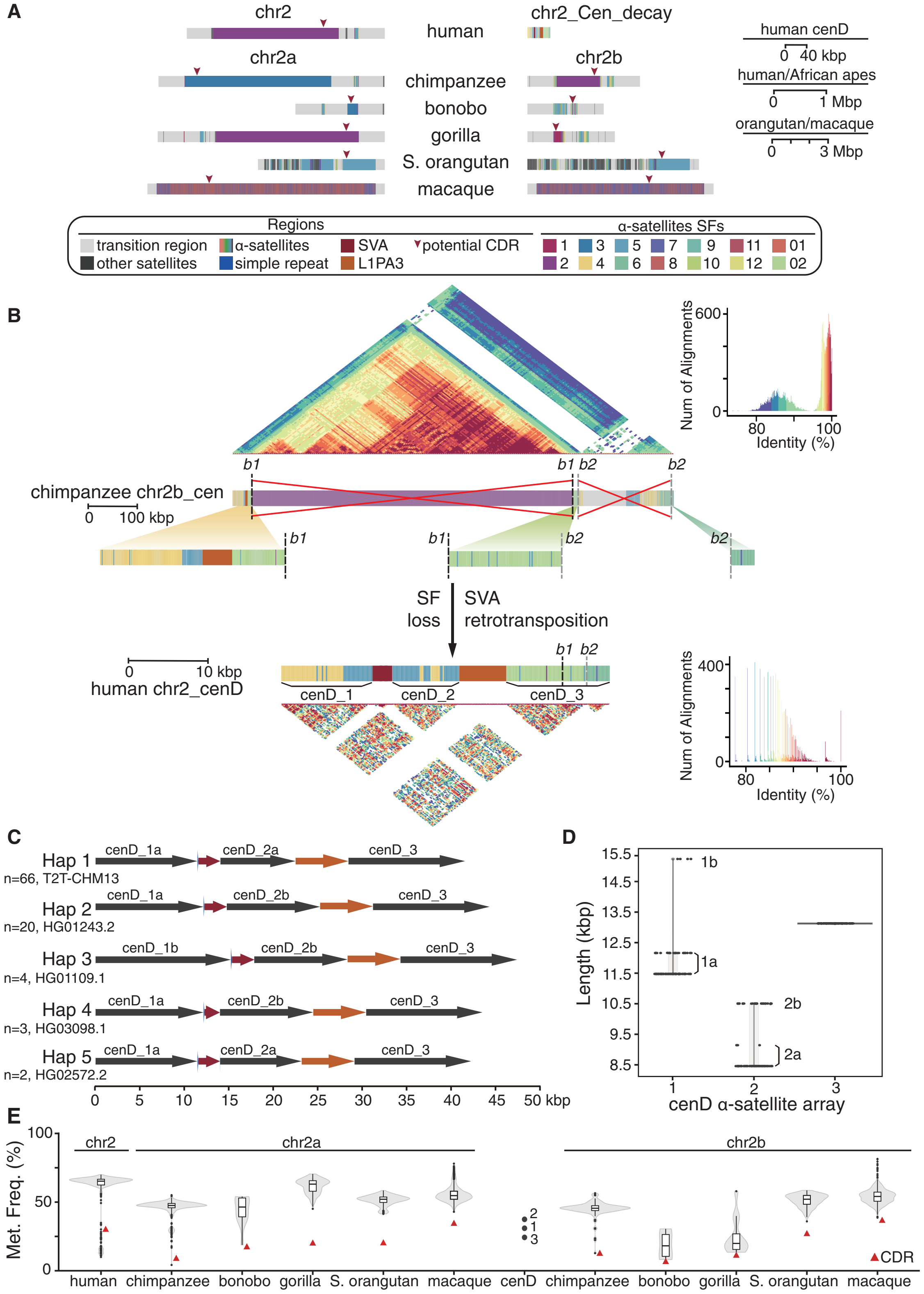

Figure 4. Comparative analysis of active and inactive centromeric regions of human chromosome 2

(A) Genomic structure and suprachromosomal family (SF) annotation of centromeres in human chr2, degenerate site (chr2:132644386–132685996), and NHP chr2a/chr2b. Different SFs are shown by various colors, with centromere dip regions (CDRs) marked by arrows.

(legend continued on next page)

are supported by long-read isoform sequencing (Iso-Seq) transcript data from CHM13hTERT[54] and kidney tissue from ENCODE.[55] In addition, methylation analysis demarcates a prominent CpG island showing the promoters/enhancers of *PGM5P4*, as identified using ONT reads from the T2T-CHM13 cell line[54] (Figure S25).

To explore the potential function of the fusion site, we used CRISPR-Cas9 to delete this region in CN1[56] induced pluripotent stem cells (iPSCs), followed by directed differentiation into neural progenitor cells (NPCs) for transcriptomic analysis (Figure 5A). One pair of single guide RNAs (sgRNAs; L-sg and R-sg) was designed for the depletion (Figure 5B), and the heterozygous depletion of the fusion site was confirmed by PCR and Sanger sequencing (Figure 5C; Table S5A). For each condition (wild type and depletion), we selected three independent monoclonal cells as biological repeats. We then conducted RNA-seq, generating 100.3, 106.2, and 104.6 million reads for the control cell lines and 137.8, 130.3, and 133.1 million reads for the fusion site deletion cell lines (Figures S26A–S26C; Table S5B).

Differential gene expression analysis identified 547 upregulated and 869 downregulated genes using default settings in the differentially expressed gene (DEG) pipeline (Figures 5D and S26D; Table S5C; STAR Methods). To reduce potential confounding from lowly expressed genes, we excluded DEGs with transcript per million (TPM) values in the lowest 50% of the transcriptome (TPM > 1), resulting in a refined set of 99 upregulated and 178 downregulated genes (Figures S26E and S26H). Functional enrichment analysis revealed that the upregulated genes were significantly associated with pattern specification processes (adjusted $p = 8.61 \times 10^{-5}$) (Figures 5E and S26I), whereas the downregulated genes were enriched for pathways related to neural development and organization, including forebrain development (adjusted $p = 8.02 \times 10^{-15}$) and axonogenesis (adjusted $p = 4.92 \times 10^{-14}$) (Figures 5F and S26J). These findings suggest that deletion of the fusion site may influence transcriptional programs involved in neural development, potentially contributing to phenotypic divergence between humans and NHPs.

DISCUSSION

In previous NHP assemblies, SD homologous to SD_fusion_A/B/C were largely collapsed (63 out of 74 cases), and subtelomeric repetitive sequences remained unresolved (Figures S27 and S28; Tables S6A–S6C). Complete T2T sequence for chr2, chr2a, and chr2b among the great apes[38–40] (average quality value = 72.1) allowed us to systematically examine the complex genomic architecture and further refine the evolutionary history of the human-specific chr2 fusion event. There are three important conclusions. First, the fusion event was intimately associated with SDs that have been restructuring genomes and chromosomes throughout great ape evolution.[27,28] The 109 kbp fusion site in humans consists of three independent SDs, each with distinct trajectories in different ape lineages, and these are further embedded in a larger duplication block of ~455 kbp. All SDs are highly variable in copy number among the great apes, and many have been reused as breakpoint sequences during great ape evolution. Although cytogenetic studies previously reported two pericentric inversions on ancestral chr2a and chr2b,[27] we observe that the flanking region at the human fusion site (chr2a, proximal side) shares 98.4% identity with an SD flanking the pericentric inversion that distinguishes Sumatran orangutan chr2b from gorilla chr2b. Moreover, SD_fusion_A shares 98.05% identity with the SD spacer sequence of the *Pan* subterminal heterochromatic caps, where it expanded to hundreds of copies in the chimpanzee and bonobo lineages but not in gorillas.

Second, we show that the fusion site has been strongly subjected to ILS. All three SDs, for example, show phylogenetic signatures consistent with ILS with a maximum occurring at the fusion site, suggesting that the fusion event occurred >5 mya. Thus, the fusion event is not a recent evolutionary event but potentially occurred during African great ape speciation when the effective population size was predicted to be much larger than contemporary ape populations.[37,38] Given fossil evidence that *Australopithecus* existed around 2–4 mya, *Paranthropus* around 1–3 mya, and the earliest *Homo* fossils around 2–3 mya,[57–59] we speculate that this fusion event probably did not arise in the genus *Homo* but rather occurred in ancestral great ape populations.

Third, the fusion site was associated with extensive subtelomeric satellite turnover and epigenetic differences among great apes. The two satellite motifs present in the subtelomeric regions of gorilla chr2a and chr2b are distinct from each other: one resembles the subtelomeric repetitive regions of orangutans, while the other resembles the genomic architecture of *Pan* species. SD_fusion_A, in part, defines the chr2 fusion site but also associates with large tracts of hypermethylated pCht satellite repeats that define subterminal heterochromatin in chimpanzees and bonobos[38] (Figure S29). The juxtaposition of novel SDs at the fusion site and the shift from heterochromatic or acrocentric DNA at the termini of chr2a and chr2b led to methylation differences among the great apes at this locus. Indeed, our depletion experiments in humans show consistent gene expression changes, suggesting that the euchromatization of the fusion site may have had more global genome-wide regulatory effects.

It is well established that fusion events can be mediated by SDs and other repetitive sequences.[52,60,61] However, to our knowledge, this study presents the first evidence of SDs subject

(B) Comparison of the centromere in chimpanzee chr2b with the human degenerate centromeric region. The middle image shows the SF annotations for the chimpanzee centromere and human centromere degenerate region. Potential α-satellite deletion breakpoints (*b1* and *b2*) are indicated with dotted lines. The heatmaps of each region are shown on the top and bottom, respectively.
(C) Five distinct structural haplotypes of the degenerate centromere site in humans.
(D) Lengths of three α-satellite arrays in humans.
(E) The methylation degree of HOR across NHPs and of three α-satellite arrays at the degenerate centromere site. The red triangle represents the methylation frequency of CDRs in each chromosome (Met. Freq., methylation frequency).
See also Figures S22 and S23 and Table S4.

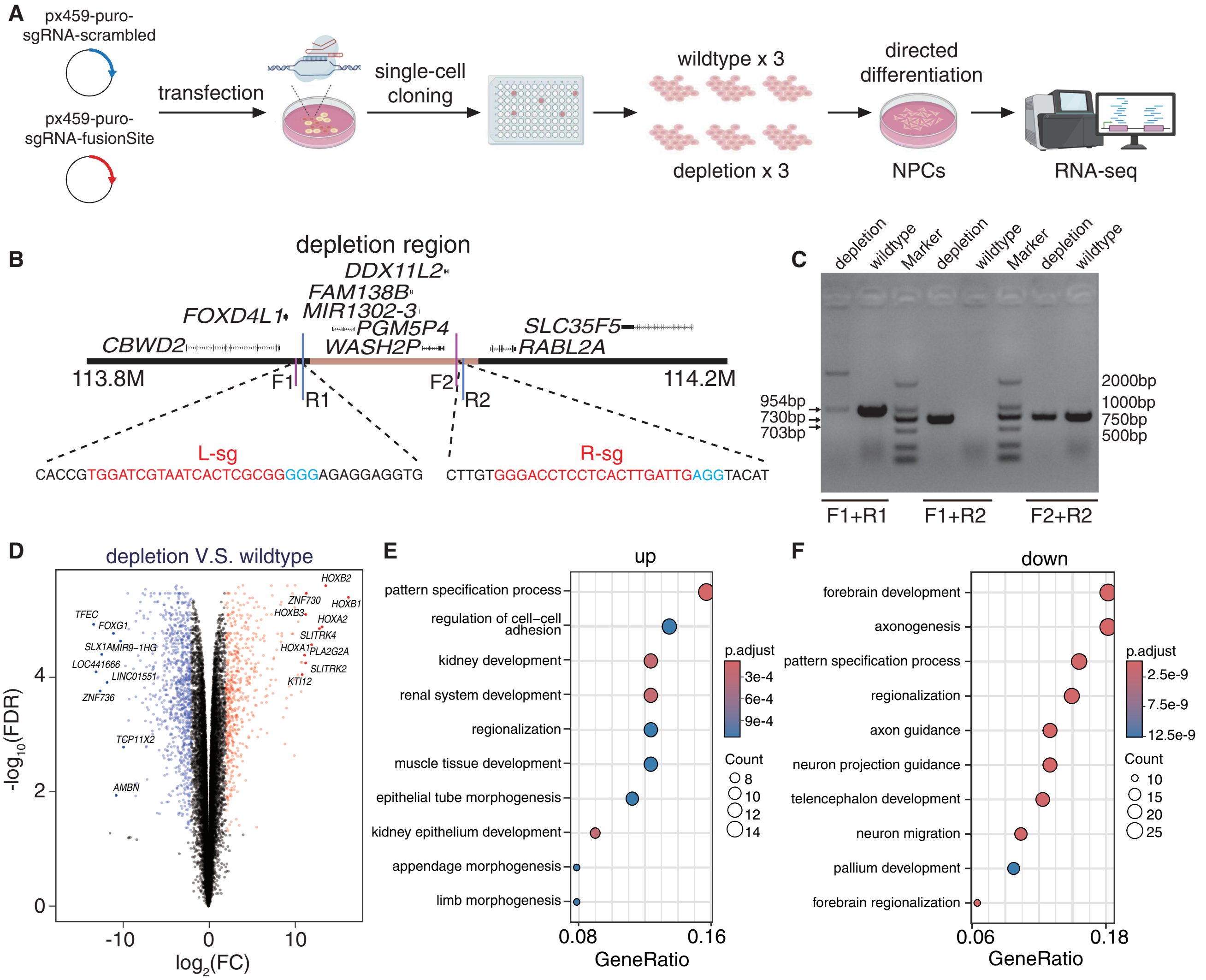

Figure 5. Fusion site knockout and gene expression alteration

(A) Schematic representation of the depletion experiments performed in CN1 cell lines. NPCs, neural progenitor cells.

(B) One sgRNA pair (L-sg and R-sg) was designed for the depletion experiments. Red sequences indicate sgRNA, while blue sequences indicate PAM sites.

(C) The PCR analysis confirms heterozygous depletion in cell lines. WT, wild-type cell lines.

(D) Differentially expressed genes (DEGs) are shown in volcano plot. Genes with $\log_2$ fold change ($\log_2$FC) values > 2 and false discovery rate (FDR) < 0.05 are highlighted in red (upregulated) and blue (downregulated).

(E and F) The GO enrichment results of (E) upregulated and (F) downregulated DEGs. Adjusted *p* value is computed and visualized by color from red to blue. See also Figures S24–S26 and Table S5.

to ILS being involved in a speciation or evolutionary divergence event. Based on our observations, we propose two evolutionary scenarios for the formation of human chr2. In the first, the large effective population size of the human-*Pan*-gorilla ancestral population facilitated the coexistence of several NHP chr2a and chr2b subtelomeric structural haplotypes 5–7 mya. In one of these structural configurations, SD_fusion_A and SD_fusion_B became juxtaposed and duplicated to the subtelomeric region of human-*Pan*-gorilla ancestral chr2a prior to the fusion event (Figure 6A). Similarly, SD_fusion_C was duplicated to the subtelomeric region of ancestral chr2b (where the full-length structure is still retained subtelomerically in bonobo); yet, it is no longer located on other nonhuman great ape chr2b due to the exchange of subtelomeric SDs and evolutionary turnover of satellite DNA.[27,62] Subsequently, the telomeric and TAR1 sequences in SD_fusion_B and SD_fusion_C mediated the fusion (Figure 6A). In the *Pan* and gorilla lineages, chr2a and chr2b (only in chimpanzee and bonobo) experienced a different evolutionary trajectory associated with the formation of subterminal heterochromatic caps. Previous studies[27,38] have shown that the heterochromatic caps likely evolved independently in both gorilla and chimpanzee, albeit convergently with a similar architecture where hundreds of kilobase pairs of satellite pCht DNA are punctuated by a ~30 kbp SD spacer region that defines a pocket of

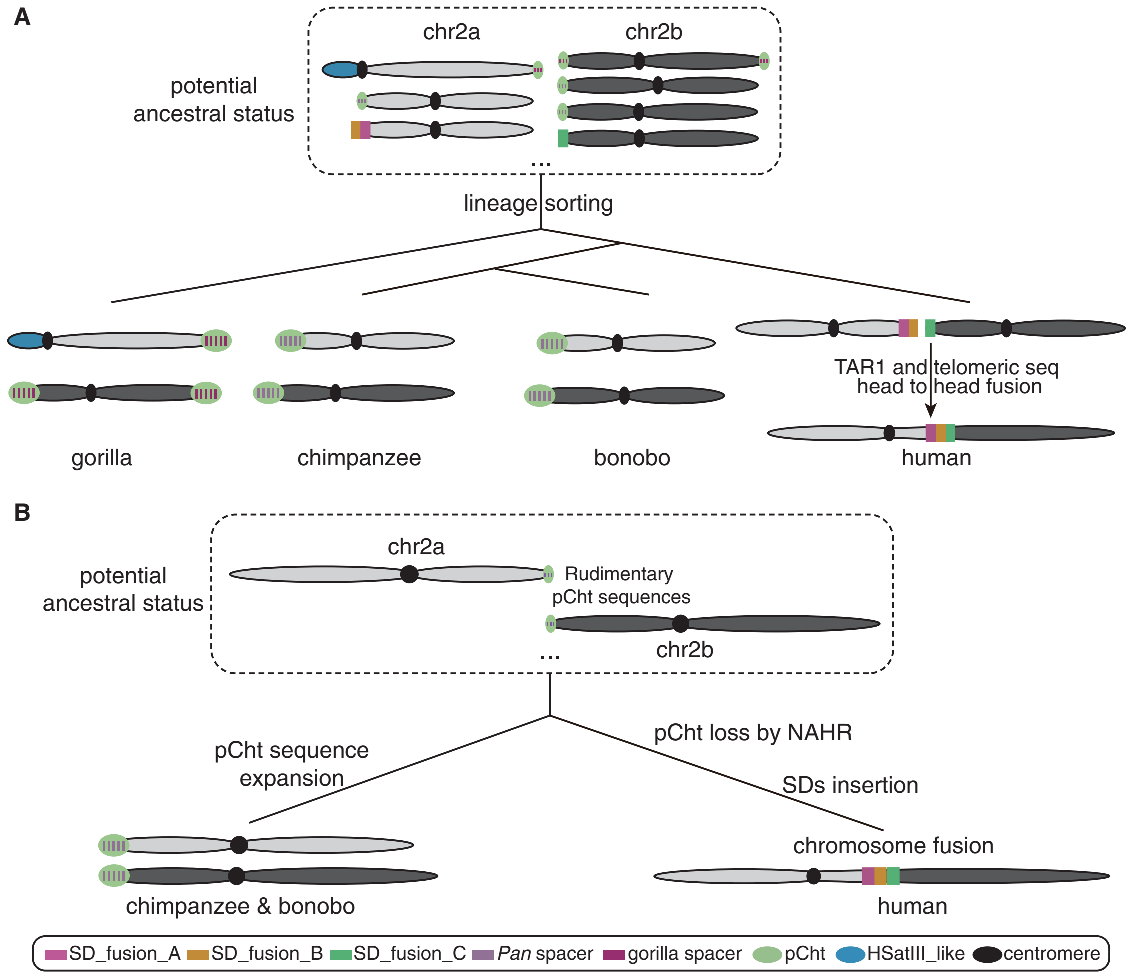

Figure 6. Models for chromosome 2 evolution

Two different models are depicted for the origin of the human chromosome 2 (chr2) fusion.

(A) In the last common ancestor of humans, bonobos, chimpanzees, and gorillas, diverse ancestral genomic structures emerged at the ends of chr2a and chr2b. In the human ancestral lineage, chr2a and chr2b consisted of complex SD blocks that emerged as a result of ILS and then became juxtaposed by a telomere-to-telomere fusion.

(B) Rudimentary pCht sequences were present in the ancestral lineage of humans and chimpanzees in association with SD_fusion_A. Nonallelic homologous recombination (NAHR) occurred between these in the human lineage, eliminating pCht in humans but retaining SD_fusion_A, with other SDs subsequently duplicating to the location. In chimpanzees, an independent expansion of the SD spacers and pCht sequences occurred, leading to the formation of the subterminal heterochromatic caps.

See also Figures S27–S30 and Tables S6A–S6C.

hypomethylation. The SD spacers in gorilla and chimpanzee subterminal heterochromatin caps are distinct, but in both chimpanzee and bonobo, the SD spacers represent a derivative of the SD_fusion_A, confirming that this sequence was present subtelomerically for chr2a in the human-*Pan* ancestor.

Alternatively, the SDs evolved similarly, but in the common ancestor of human and *Pan*, there was an incipient association with rudimentary pCht satellite sequences with the SD_fusion_A sequence that mediated the exchange between ancestral chr2a and chr2b via nonallelic homologous recombination (NAHR) or ectopic exchange. Subsequently, during NAHR between full-length SD_fusion_A elements, the pCht regions were lost in the human lineage, and two additional SDs were inserted at the fusion breakpoint region (Figure 6B). In the *Pan* lineage, a portion of the SD_fusion_A became hyperexpanded, defining the subterminal SD spacer region of all heterochromatic caps in bonobo and chimpanzee. In support of this model, it is known that in both chimpanzee and gorilla, the subterminal satellite DNA forms unique post-bouquet structures in germ cells and are hotspots of ectopic exchange between nonhomologous chromosomes.[63] If such exchanges occurred at the edge of the incipient subterminal heterochromatic caps before there were many copies of the subterminal satellite DNA, it would help explain the absence of the pCht sequence in the human genome—i.e., the fusion of chr2a and chr2b helped eliminate the potential for the formation of subterminal heterochromatic caps.

These two models differ in terms of which repetitive sequences played the primary role in mediating the fusion event: the first model favors SDs, while the second emphasizes pCht-like sequences. However, both models support the presence of SDs in the LCA and suggest that ILS, potentially facilitated by a large effective population size, played a key role.[37,38] These aspects helped maintain and diversify karyotypic structures of the ancestral chr2a and chr2b for possibly millions of years. The divergent fates of the subtelomeric repetitive regions of these ancestral chromosomes, e.g., pCht expansion and SD spacer insertions in the *Pan* lineage or the SD insertions in the human fusion site, may have contributed to divergence between humans and NHPs, potentially involving ILS (Figure S30). In this light, it is interesting that we document an asymmetric ILS pattern, with an increase in ILS segments on the distal side (chr2b) and a decrease on the proximal side (chr2a) of the fusion site. This suggests diverse evolutionary trajectories for the ancestral subtelomeric regions of NHP chr2a and chr2b, highlighting the potentially important role of both SDs and ILS in understanding chromosomal evolution, evolutionary divergence, speciation, and sequence turnover.

Limitations of the study

Speciation is not an instantaneous process—it typically requires extended periods of population divergence and is often more complex than expected. Our study shows that SDs have undergone lineage-specific sorting in extant apes, including humans, and may have played a key role in facilitating the chr2 fusion in the (ancestral) human lineage. However, the exact timing of when this fusion became nearly fixed in the (ancestral) human population remains unknown, but our results suggest that it is ancient and that it occurred early in African ape speciation >5 mya.

Our study supports the idea that ILS plays a crucial role in fusion site formation based on current genomic data from living apes. However, the large population size of the common ancestor of great apes complicates the evolutionary scenario. For example, recent studies have shown that ghost introgression occurred during great ape speciation.[64,65] Therefore, we have sufficient reason to guess that ILS is not the only evolutionary force involved in this fusion formation, and other forces (e.g., ancestral introgression) could also be associated with the fusion. Importantly, our study is the first to propose that ILS is involved in chr2 fusion and may have facilitated great ape speciation and the origins of humans.

Meanwhile, while our study demonstrates that depletion of the fusion site leads to gene expression changes in NPC cells, the underlying molecular mechanisms—such as potential alterations in 3D genome structure or functional roles of genes within the fusion site—remain to be elucidated.

RESOURCE AVAILABILITY

Lead contact

Further information and requests for data should be directed to and will be fulfilled by the lead contact, Yafei Mao (yafmao@sjtu.edu.cn).

Materials availability

This study generated wild-type and fusion-site-depletion NPCs from CN1 iPSC cell lines. Requests should be directed to Yafei Mao (yafmao@sjtu.edu.cn).

Data and code availability

- The T2T primate genomes used in this study are available from GenBank via the following accessions: GCA_009914755.4, GCA_028858775.2, GCA_028885625.2, GCA_028885655.2, GCA_029281585.2, GCA_029289425.2, and GCA_037993035.1. The T2T primate genome assemblies are also available on GitHub (https://github.com/marbl/Primates and https://github.com/zhang-shilong/T2T-MFA8). The Neanderthal and Denisovan genomes used are available from https://www.eva.mpg.de/genetics/genome-projects. The Iso-Seq data are available on ENCODE (tissues: ENCFF492BYP, ENCFF306ZPP, ENCFF318SKH, and CHM13-T2T hTERT Iso-Seq: SRR12519035 and SRR12519036).
- This study did not generate any unique code.

ACKNOWLEDGMENTS

We thank Tonia Brown for editing this manuscript. We thank the HPRC and Primate T2T Consortium for providing the long-read human and great ape genome assemblies. This work was supported, in part, by grants from the National Natural Science Foundation of China (32370658); the Natural Science Foundation of Chongqing, China (CSTB2024NSCQ-JQX0004); the Shanghai Jiao Tong University 2030 Initiative (WH510363003/016); the Computational Biology Program (24JS2840300) of the Science and Technology Commission of Shanghai Municipality (STCSM); the SJTU Global Initiative Fund (Type B); Yongxin Youth Award Fund; Zhongying Young Scholar Program; and the New Cornerstone Science Foundation through the XPLORER PRIZE to Y.M. This work was supported, in part, by grants from the National Key Research and Development Program of China (2022YFF0710901), a National Natural Science Foundation of China grant (82021001), the Biological Resources Program of the Chinese Academy of Sciences (KFJ-BRP-005), and the National Science and Technology Innovation 2030 Major Program (2021ZD0200900) to Q.S. This work is partially sponsored by the Shanghai Rising-Star Program (24YF2721800) to K.M. Research reported in this publication was supported, in part, by the National Human Genome Research Institute of the US National Institutes of Health (NIH) under Award Number R01HG002385 to E.E.E. The content is solely the responsibility of the authors and does not necessarily represent the official views of the NIH. E.E.E. is an investigator of the Howard Hughes Medical Institute. This article is subject to HHMI's Open Access to Publications policy. HHMI lab heads have previously granted a nonexclusive CC BY 4.0 license to the public and a sublicensable license to HHMI in their research articles. Pursuant to those licenses, the author accepted manuscript of this article can be made freely available under a CC BY 4.0 license immediately upon publication. The computations in this study were run on the Siyuan-1, supported by the Center for High Performance Computing at Shanghai Jiao Tong University.

AUTHOR CONTRIBUTIONS

Y.M., E.E.E., and Q.S. conceived the project; Z.Y., X.J., X.Y., K.M., S.Z., J.C., J.H., L.F., J.Z., M.V., and Y.M. contributed to the syntenic comparison, fusion site characterization, ILS, and subtelomeric repetitive region analyses; Z.Y., L.Z., X.J., Y.L., Y.N., X.B., and Q.S. contributed to the fusion site depletion analysis; D.Y. and E.E.E. generated the genome assemblies of great apes; X.J., G.Z., and Y.M. analyzed the centromeres; Y.M. and E.E.E. wrote the draft manuscript with contributions from the other authors; and all authors read and approved the manuscript.

DECLARATION OF INTERESTS

E.E.E. is a scientific advisory board (SAB) member of Variant Bio, Inc., and a member of the *Cell* advisory board.

STAR★METHODS

Detailed methods are provided in the online version of this paper and include the following:

- KEY RESOURCES TABLE
- METHOD DETAILS
  - Data resources and comparative analysis
  - Fusion site characterization and population analysis
  - SD annotations and phylogenetic analysis
  - Subtelomeric repetitive region characterization, methylation analysis, and TE annotation
  - Centromere analysis
  - Fusion site KO experiments
  - iPSCs differentiation into neural progenitor cells
  - RNA preparation and RNA-seq data analysis

SUPPLEMENTAL INFORMATION

Supplemental information can be found online at https://doi.org/10.1016/j.xgen.2025.101079.

Received: January 14, 2025
Revised: June 15, 2025
Accepted: November 3, 2025
Published: December 2, 2025

REFERENCES

1. Bush, G.L., Case, S.M., Wilson, A.C., and Patton, J.L. (1977). Rapid speciation and chromosomal evolution in mammals. Proc. Natl. Acad. Sci. USA *74*, 3942–3946. https://doi.org/10.1073/pnas.74.9.3942.
2. Damas, J., Corbo, M., Kim, J., Turner-Maier, J., Farré, M., Larkin, D.M., Ryder, O.A., Steiner, C., Houck, M.L., Hall, S., et al. (2022). Evolution of the ancestral mammalian karyotype and syntenic regions. Proc. Natl. Acad. Sci. USA *119*, e2209139119. https://doi.org/10.1073/pnas.2209139119.
3. Ferguson-Smith, M.A., and Trifonov, V. (2007). Mammalian karyotype evolution. Nat. Rev. Genet. *8*, 950–962. https://doi.org/10.1038/nrg2199.
4. Leibowitz, M.L., Zhang, C.-Z., and Pellman, D. (2015). Chromothripsis: A New Mechanism for Rapid Karyotype Evolution. Annu. Rev. Genet. *49*, 183–211. https://doi.org/10.1146/annurev-genet-120213-092228.
5. Painter, T.S., and Stone, W. (1935). CHROMOSOME FUSION AND SPECIATION IN DROSOPHILAE. Genetics *20*, 327–341. https://doi.org/10.1093/genetics/20.4.327.
6. Rieseberg, L.H. (2001). Chromosomal rearrangements and speciation. Trends Ecol. Evol. *16*, 351–358. https://doi.org/10.1016/S0169-5347(01)02187-5.
7. Watkins, T.B.K., Lim, E.L., Petkovic, M., Elizalde, S., Birkbak, N.J., Wilson, G.A., Moore, D.A., Grönroos, E., Rowan, A., Dewhurst, S.M., et al. (2020). Pervasive chromosomal instability and karyotype order in tumour evolution. Nature *587*, 126–132. https://doi.org/10.1038/s41586-020-2698-6.
8. Baker, R.J., and Bickham, J.W. (1986). Speciation by monobrachial centric fusions. Proc. Natl. Acad. Sci. USA *83*, 8245–8248. https://doi.org/10.1073/pnas.83.21.8245.
9. Kirkpatrick, M., and Barton, N. (2006). Chromosome Inversions, Local Adaptation and Speciation. Genetics *173*, 419–434. https://doi.org/10.1534/genetics.105.047985.
10. Schubert, I. (2007). Chromosome evolution. Curr. Opin. Plant Biol. *10*, 109–115. https://doi.org/10.1016/j.pbi.2007.01.001.
11. Bailey, J.A., and Eichler, E.E. (2006). Primate segmental duplications: crucibles of evolution, diversity and disease. Nat. Rev. Genet. *7*, 552–564. https://doi.org/10.1038/nrg1895.
12. Baird, D.M. (2018). Telomeres and genomic evolution. Philos. Trans. R. Soc. Lond. B Biol. Sci. *373*, 20160437. https://doi.org/10.1098/rstb.2016.0437.
13. Chikashige, Y., Ding, D.Q., Imai, Y., Yamamoto, M., Haraguchi, T., and Hiraoka, Y. (1997). Meiotic nuclear reorganization: switching the position of centromeres and telomeres in the fission yeast Schizosaccharomyces pombe. EMBO J. *16*, 193–202. https://doi.org/10.1093/emboj/16.1.193.
14. Lee, C., Sasi, R., and Lin, C.C. (1993). Interstitial localization of telomeric DNA sequences in the Indian muntjac chromosomes: further evidence for tandem chromosome fusions in the karyotypic evolution of the Asian muntjacs. Cytogenet. Cell Genet. *63*, 156–159. https://doi.org/10.1159/000133525.
15. Zhao, N., Yin, G., Liu, C., Zhang, W., Shen, Y., Wang, D., Lin, Z., Yang, J., Mao, J., Guo, R., et al. (2023). Critically short telomeres derepress retrotransposons to promote genome instability in embryonic stem cells. Cell Discov. *9*, 45. https://doi.org/10.1038/s41421-023-00538-y.
16. Augustijnen, H., Bätscher, L., Cesanek, M., Chkhartishvili, T., Dincă, V., Iankoshvili, G., Ogawa, K., Vila, R., Klopfstein, S., De Vos, J.M., and Lucek, K. (2024). A macroevolutionary role for chromosomal fusion and fission in Erebia butterflies. Sci. Adv. *10*, eadl0989. https://doi.org/10.1126/sciadv.adl0989.
17. Mudd, A.B., Bredeson, J.V., Baum, R., Hockemeyer, D., and Rokhsar, D.S. (2020). Analysis of muntjac deer genome and chromatin architecture reveals rapid karyotype evolution. Commun. Biol. *3*, 480. https://doi.org/10.1038/s42003-020-1096-9.
18. Wright, C.J., Stevens, L., Mackintosh, A., Lawniczak, M., and Blaxter, M. (2024). Comparative genomics reveals the dynamics of chromosome evolution in Lepidoptera. Nat. Ecol. Evol. *8*, 777–790. https://doi.org/10.1038/s41559-024-02329-4.
19. Dymond, J.S., Richardson, S.M., Coombes, C.E., Babatz, T., Muller, H., Annaluru, N., Blake, W.J., Schwerzmann, J.W., Dai, J., Lindstrom, D.L., et al. (2011). Synthetic chromosome arms function in yeast and generate phenotypic diversity by design. Nature *477*, 471–476. https://doi.org/10.1038/nature10403.
20. Luo, J., Sun, X., Cormack, B.P., and Boeke, J.D. (2018). Karyotype engineering by chromosome fusion leads to reproductive isolation in yeast. Nature *560*, 392–396. https://doi.org/10.1038/s41586-018-0374-x.
21. Wang, L.-B., Li, Z.-K., Wang, L.-Y., Xu, K., Ji, T.-T., Mao, Y.-H., Ma, S.-N., Liu, T., Tu, C.-F., Zhao, Q., et al. (2022). A sustainable mouse karyotype created by programmed chromosome fusion. Science *377*, 967–975. https://doi.org/10.1126/science.abm1964.
22. Zhang, X.M., Yan, M., Yang, Z., Xiang, H., Tang, W., Cai, X., Wu, Q., Liu, X., Pei, G., and Li, J. (2022). Creation of artificial karyotypes in mice reveals robustness of genome organization. Cell Res. *32*, 1026–1029. https://doi.org/10.1038/s41422-022-00722-x.
23. Dutrillaux, B., Rethoré, M.O., and Lejeune, J. (1975). [Comparison of the karyotype of the orangutan (Pongo pygmaeus) to those of man, chimpazee, and gorilla]. Ann. Genet. *18*, 153–161.
24. IJdo, J.W., Baldini, A., Ward, D.C., Reeders, S.T., and Wells, R.A. (1991). Origin of human chromosome 2: an ancestral telomere-telomere fusion. Proc. Natl. Acad. Sci. USA *88*, 9051–9055. https://doi.org/10.1073/pnas.88.20.9051.
25. Watson, J.D. (1990). The Human Genome Project: Past, Present, and Future. Science *248*, 44–49. https://doi.org/10.1126/science.2181665.
26. Poszewiecka, B., Gogolewski, K., Karolak, J.A., Stankiewicz, P., and Gambin, A. (2023). PhaseDancer: a novel targeted assembler of segmental duplications unravels the complexity of the human chromosome 2 fusion going from 48 to 46 chromosomes in hominin evolution. Genome Biol. *24*, 205. https://doi.org/10.1186/s13059-023-03022-8.
27. Ventura, M., Catacchio, C.R., Sajjadian, S., Vives, L., Sudmant, P.H., Marques-Bonet, T., Graves, T.A., Wilson, R.K., and Eichler, E.E. (2012). The evolution of African great ape subtelomeric heterochromatin and the fusion of human chromosome 2. Genome Res. *22*, 1036–1049. https://doi.org/10.1101/gr.136556.111.
28. Fan, Y., Linardopoulou, E., Friedman, C., Williams, E., and Trask, B.J. (2002). Genomic Structure and Evolution of the Ancestral Chromosome Fusion Site in 2q13–2q14.1 and Paralogous Regions on Other Human Chromosomes. Genome Res. *12*, 1651–1662. https://doi.org/10.1101/gr.337602.
29. Fan, Y., Newman, T., Linardopoulou, E., and Trask, B.J. (2002). Gene Content and Function of the Ancestral Chromosome Fusion Site in

Human Chromosome 2q13–2q14.1 and Paralogous Regions. Genome Res. *12*, 1663–1672. https://doi.org/10.1101/gr.338402.

30. Martin, C.L., Wong, A., Gross, A., Chung, J., Fantes, J.A., and Ledbetter, D.H. (2002). The Evolutionary Origin of Human Subtelomeric Homologies—or Where the Ends Begin. Am. J. Hum. Genet. *70*, 972–984. https://doi.org/10.1086/339768.
31. Royle, N.J., Baird, D.M., and Jeffreys, A.J. (1994). A subterminal satellite located adjacent to telomeres in chimpanzees is absent from the human genome. Nat. Genet. *6*, 52–56. https://doi.org/10.1038/ng0194-52.
32. Miga, K.H. (2017). Chromosome-Specific Centromere Sequences Provide an Estimate of the Ancestral Chromosome 2 Fusion Event in Hominin Genomes. J. Hered. *108*, 45–52. https://doi.org/10.1093/jhered/esw039.
33. Wang, H., Xing, J., Grover, D., Hedges, D.J., Han, K., Walker, J.A., and Batzer, M.A. (2005). SVA Elements: A Hominid-specific Retroposon Family. J. Mol. Biol. *354*, 994–1007. https://doi.org/10.1016/j.jmb.2005.09.085.
34. Poszewiecka, B., Gogolewski, K., Stankiewicz, P., and Gambin, A. (2022). Revised time estimation of the ancestral human chromosome 2 fusion. BMC Genom. *23*, 616. https://doi.org/10.1186/s12864-022-08828-7.
35. Aganezov, S., Yan, S.M., Soto, D.C., Kirsche, M., Zarate, S., Avdeyev, P., Taylor, D.J., Shafin, K., Shumate, A., Xiao, C., et al. (2022). A complete reference genome improves analysis of human genetic variation. Science *376*, eabl3533. https://doi.org/10.1126/science.abl3533.
36. Vollger, M.R., Guitart, X., Dishuck, P.C., Mercuri, L., Harvey, W.T., Gershman, A., Diekhans, M., Sulovari, A., Munson, K.M., Lewis, A.P., et al. (2022). Segmental duplications and their variation in a complete human genome. Science *376*, eabj6965. https://doi.org/10.1126/science.abj6965.
37. Mao, Y., Catacchio, C.R., Hillier, L.W., Porubsky, D., Li, R., Sulovari, A., Fernandes, J.D., Montinaro, F., Gordon, D.S., Storer, J.M., et al. (2021). A high-quality bonobo genome refines the analysis of hominid evolution. Nature *594*, 77–81. https://doi.org/10.1038/s41586-021-03519-x.
38. Yoo, D., Rhie, A., Hebbar, P., Antonacci, F., Logsdon, G.A., Solar, S.J., Antipov, D., Pickett, B.D., Safonova, Y., Montinaro, F., et al. (2025). Complete sequencing of ape genomes. Nature *641*, 401–418. https://doi.org/10.1038/s41586-025-08816-3.
39. Zhang, S., Xu, N., Fu, L., Yang, X., Ma, K., Li, Y., Yang, Z., Li, Z., Feng, Y., Jiang, X., et al. (2025). Integrated analysis of the complete sequence of a macaque genome. Nature *640*, 714–721. https://doi.org/10.1038/s41586-025-08596-w.
40. Makova, K.D., Pickett, B.D., Harris, R.S., Hartley, G.A., Cechova, M., Pal, K., Nurk, S., Yoo, D., Li, Q., Hebbar, P., et al. (2024). The complete sequence and comparative analysis of ape sex chromosomes. Nature *630*, 401–411. https://doi.org/10.1038/s41586-024-07473-2.
41. Koga, A., Hirai, Y., Hara, T., and Hirai, H. (2012). Repetitive sequences originating from the centromere constitute large-scale heterochromatin in the telomere region in the siamang, a small ape. Heredity *109*, 180–187. https://doi.org/10.1038/hdy.2012.28.
42. Dutrillaux, B. (1979). Chromosomal evolution in Primates: Tentative phylogeny from Microcebus murinus (Prosimian) to man. Hum. Genet. *48*, 251–314. https://doi.org/10.1007/BF00272830.
43. Yunis, J.J., and Prakash, O. (1982). The Origin of Man: A Chromosomal Pictorial Legacy. Science *215*, 1525–1530. https://doi.org/10.1126/science.7063861.
44. Liao, W.-W., Asri, M., Ebler, J., Doerr, D., Haukness, M., Hickey, G., Lu, S., Lucas, J.K., Monlong, J., Abel, H.J., et al. (2023). A draft human pangenome reference. Nature *617*, 312–324. https://doi.org/10.1038/s41586-023-05896-x.
45. Linardopoulou, E.V., Parghi, S.S., Friedman, C., Osborn, G.E., Parkhurst, S.M., and Trask, B.J. (2007). Human Subtelomeric WASH Genes Encode a New Subclass of the WASP Family. PLoS Genet. *3*, e237. https://doi.org/10.1371/journal.pgen.0030237.
46. Vollger, M.R., Dishuck, P.C., Harvey, W.T., DeWitt, W.S., Guitart, X., Goldberg, M.E., Rozanski, A.N., Lucas, J., Asri, M., and Human Pangenome Reference Consortium; and et al.. (2023). Increased mutation and gene conversion within human segmental duplications. Nature *617*, 325–334. https://doi.org/10.1038/s41586-023-05895-y.
47. Chen, F.C., and Li, W.H. (2001). Genomic divergences between humans and other hominoids and the effective population size of the common ancestor of humans and chimpanzees. Am. J. Hum. Genet. *68*, 444–456. https://doi.org/10.1086/318206.
48. Hobolth, A., Dutheil, J.Y., Hawks, J., Schierup, M.H., and Mailund, T. (2011). Incomplete lineage sorting patterns among human, chimpanzee, and orangutan suggest recent orangutan speciation and widespread selection. Genome Res. *21*, 349–356. https://doi.org/10.1101/gr.114751.110.
49. Yang, Z. (2002). Likelihood and Bayes estimation of ancestral population sizes in hominoids using data from multiple loci. Genetics *162*, 1811–1823. https://doi.org/10.1093/genetics/162.4.1811.
50. Rivas-González, I., Rousselle, M., Li, F., Zhou, L., Dutheil, J.Y., Munch, K., Shao, Y., Wu, D., Schierup, M.H., and Zhang, G. (2023). Pervasive incomplete lineage sorting illuminates speciation and selection in primates. Science *380*, eabn4409. https://doi.org/10.1126/science.abn4409.
51. Xu, Y., Wei, Y., Zhou, Z., Cai, X., Boden, S.A., Umer, M.J., Safdar, L.B., Liu, Y., Jin, D., Hou, Y., et al. (2024). Widespread incomplete lineage sorting and introgression shaped adaptive radiation in the Gossypium genus. Plant Commun. *5*, 100728. https://doi.org/10.1016/j.xplc.2023.100728.
52. Chiatante, G., Giannuzzi, G., Calabrese, F.M., Eichler, E.E., and Ventura, M. (2017). Centromere Destiny in Dicentric Chromosomes: New Insights from the Evolution of Human Chromosome 2 Ancestral Centromeric Region. Mol. Biol. Evol. *34*, 1669–1681. https://doi.org/10.1093/molbev/msx108.
53. Lonsdale, J., Thomas, J., Salvatore, M., Phillips, R., Lo, E., Shad, S., Hasz, R., Walters, G., Garcia, F., Young, N., et al. (2013). The Genotype-Tissue Expression (GTEx) project. Nat. Genet. *45*, 580–585. https://doi.org/10.1038/ng.2653.
54. Logsdon, G.A., Vollger, M.R., Hsieh, P., Mao, Y., Liskovykh, M.A., Koren, S., Nurk, S., Mercuri, L., Dishuck, P.C., Rhie, A., et al. (2021). The structure, function and evolution of a complete human chromosome 8. Nature *593*, 101–107. https://doi.org/10.1038/s41586-021-03420-7.
55. Luo, Y., Hitz, B.C., Gabdank, I., Hilton, J.A., Kagda, M.S., Lam, B., Myers, Z., Sud, P., Jou, J., Lin, K., et al. (2020). New developments on the Encyclopedia of DNA Elements (ENCODE) data portal. Nucleic Acids Res. *48*, D882–D889. https://doi.org/10.1093/nar/gkz1062.
56. Yang, C., Zhou, Y., Song, Y., Wu, D., Zeng, Y., Nie, L., Liu, P., Zhang, S., Chen, G., Xu, J., et al. (2023). The complete and fully-phased diploid genome of a male Han Chinese. Cell Res. *33*, 745–761. https://doi.org/10.1038/s41422-023-00849-5.
57. Alemseged, Z. (2023). Reappraising the palaeobiology of Australopithecus. Nature *617*, 45–54. https://doi.org/10.1038/s41586-023-05957-1.
58. Broeils, L.A., Ruiz-Orera, J., Snel, B., Hubner, N., and Van Heesch, S. (2023). Evolution and implications of de novo genes in humans. Nat. Ecol. Evol. *7*, 804–815. https://doi.org/10.1038/s41559-023-02014-y.
59. Lacruz, R.S., Stringer, C.B., Kimbel, W.H., Wood, B., Harvati, K., O'Higgins, P., Bromage, T.G., and Arsuaga, J.-L. (2019). The evolutionary history of the human face. Nat. Ecol. Evol. *3*, 726–736. https://doi.org/10.1038/s41559-019-0865-7.
60. Kopecna, O., Kubickova, S., Cernohorska, H., Cabelova, K., Vahala, J., Martinkova, N., and Rubes, J. (2014). Tribe-specific satellite DNA in non-domestic Bovidae. Chromosome Res. *22*, 277–291. https://doi.org/10.1007/s10577-014-9401-4.
61. Palacios-Gimenez, O.M., Castillo, E.R., Martí, D.A., and Cabral-de-Mello, D.C. (2013). Tracking the evolution of sex chromosome systems in Melanoplinae grasshoppers through chromosomal mapping of repetitive DNA sequences. BMC Evol. Biol. *13*, 167. https://doi.org/10.1186/1471-2148-13-167.
62. Trask, B. (1999). Fluorescence. In Genome analysis: A laboratory manual. Cold Spring Harbor, B. Birren, E.D. Green, P. Hieter, S. Klapholz, R.M. Myers, H. Riethman, J. Roskams, and S. Hybridization, eds. (NY: Cold Spring Harbor Laboratory Press), pp. 303–413.

63. Hirai, H., Hirai, Y., Udono, T., Matsubayashi, K., Tosi, A.J., and Koga, A. (2019). Structural variations of subterminal satellite blocks and their source mechanisms as inferred from the meiotic configurations of chimpanzee chromosome termini. Chromosome Res. *27*, 321–332. https://doi.org/10.1007/s10577-019-09615-z.

64. Kuhlwilm, M., Han, S., Sousa, V.C., Excoffier, L., and Marques-Bonet, T. (2019). Ancient admixture from an extinct ape lineage into bonobos. Nat. Ecol. Evol. *3*, 957–965. https://doi.org/10.1038/s41559-019-0881-7.

65. Pawar, H., Rymbekova, A., Cuadros-Espinoza, S., Huang, X., de Manuel, M., van der Valk, T., Lobon, I., Alvarez-Estape, M., Haber, M., Dolgova, O., et al. (2023). Ghost admixture in eastern gorillas. Nat. Ecol. Evol. *7*, 1503–1514. https://doi.org/10.1038/s41559-023-02145-2.

66. Li, H. (2018). Minimap2: pairwise alignment for nucleotide sequences. Bioinformatics *34*, 3094–3100. https://doi.org/10.1093/bioinformatics/bty191.

67. Danecek, P., Auton, A., Abecasis, G., Albers, C.A., Banks, E., DePristo, M.A., Handsaker, R.E., Lunter, G., Marth, G.T., Sherry, S.T., et al. (2011). The variant call format and VCFtools. Bioinformatics *27*, 2156–2158. https://doi.org/10.1093/bioinformatics/btr330.

68. Katoh, K., and Standley, D.M. (2013). MAFFT Multiple Sequence Alignment Software Version 7: Improvements in Performance and Usability. Mol. Biol. Evol. *30*, 772–780. https://doi.org/10.1093/molbev/mst010.

69. Capella-Gutiérrez, S., Silla-Martínez, J.M., and Gabaldón, T. (2009). trimAl: a tool for automated alignment trimming in large-scale phylogenetic analyses. Bioinformatics *25*, 1972–1973. https://doi.org/10.1093/bioinformatics/btp348.

70. Minh, B.Q., Schmidt, H.A., Chernomor, O., Schrempf, D., Woodhams, M.D., Von Haeseler, A., and Lanfear, R. (2020). IQ-TREE 2: New Models and Efficient Methods for Phylogenetic Inference in the Genomic Era. Mol. Biol. Evol. *37*, 1530–1534. https://doi.org/10.1093/molbev/msaa015.

71. Bouckaert, R., Vaughan, T.G., Barido-Sottani, J., Duchêne, S., Fourment, M., Gavryushkina, A., Heled, J., Jones, G., Kühnert, D., De Maio, N., et al. (2019). BEAST 2.5: An advanced software platform for Bayesian evolutionary analysis. PLoS Comput. Biol. *15*, e1006650. https://doi.org/10.1371/journal.pcbi.1006650.

72. Sweeten, A.P., Schatz, M.C., and Phillippy, A.M. (2024). ModDotPlot—rapid and interactive visualization of tandem repeats. Bioinformatics *40*, btae493. https://doi.org/10.1093/bioinformatics/btae493.

73. Numanagić, I., Gökkaya, A.S., Zhang, L., Berger, B., Alkan, C., and Hach, F. (2018). Fast characterization of segmental duplications in genome assemblies. Bioinformatics *34*, i706–i714. https://doi.org/10.1093/bioinformatics/bty586.

74. Quinlan, A.R., and Hall, I.M. (2010). BEDTools: a flexible suite of utilities for comparing genomic features. Bioinformatics *26*, 841–842. https://doi.org/10.1093/bioinformatics/btq033.

75. Gu, Z., Gu, L., Eils, R., Schlesner, M., and Brors, B. (2014). circlize implements and enhances circular visualization in R. Bioinformatics *30*, 2811–2812. https://doi.org/10.1093/bioinformatics/btu393.

76. Galili, T. (2015). dendextend: an R package for visualizing, adjusting and comparing trees of hierarchical clustering. Bioinformatics *31*, 3718–3720. https://doi.org/10.1093/bioinformatics/btv428.

77. Smith, M.R. (2020). Information theoretic generalized Robinson–Foulds metrics for comparing phylogenetic trees. Bioinformatics *36*, 5007–5013. https://doi.org/10.1093/bioinformatics/btaa614.

78. Altemose, N., Logsdon, G.A., Bzikadze, A.V., Sidhwani, P., Langley, S.A., Caldas, G.V., Hoyt, S.J., Uralsky, L., Ryabov, F.D., Shew, C.J., et al. (2022). Complete genomic and epigenetic maps of human centromeres. Science *376*, eabl4178. https://doi.org/10.1126/science.abl4178.

79. Hach, F., Hormozdiari, F., Alkan, C., Hormozdiari, F., Birol, I., Eichler, E.E., and Sahinalp, S.C. (2010). mrsFAST: a cache-oblivious algorithm for short-read mapping. Nat. Methods *7*, 576–577. https://doi.org/10.1038/nmeth0810-576.

80. Harshil Patel, J.M., Ewels, P., Garcia, M.U., Peltzer, A., Hammarén, R., Botvinnik, O., Talbot, A., Sturm, G., Zepper, M., Moreno, D., et al. (2025). nf-core/rnaseq: nf-core/rnaseq v3.19.0 -. Tungsten Turtle (3.19.0). https://doi.org/10.5281/zenodo.1400710.

81. Love, M.I., Huber, W., and Anders, S. (2014). Moderated estimation of fold change and dispersion for RNA-seq data with DESeq2. Genome Biol. *15*, 550. https://doi.org/10.1186/s13059-014-0550-8.

82. Chen, Y., Lun, A.T.L., and Smyth, G.K. (2016). From reads to genes to pathways: differential expression analysis of RNA-Seq experiments using Rsubread and the edgeR quasi-likelihood pipeline. F1000Res. *5*, 1438. https://doi.org/10.12688/f1000research.8987.2.

83. Yu, G., Wang, L.G., Han, Y., and He, Q.Y. (2012). clusterProfiler: an R package for comparing biological themes among gene clusters. OMICS *16*, 284–287. https://doi.org/10.1089/omi.2011.0118.

84. Altemose, N., Miga, K.H., Maggioni, M., and Willard, H.F. (2014). Genomic Characterization of Large Heterochromatic Gaps in the Human Genome Assembly. PLoS Comput. Biol. *10*, e1003628. https://doi.org/10.1371/journal.pcbi.1003628.

85. Ewels, P.A., Peltzer, A., Fillinger, S., Patel, H., Alneberg, J., Wilm, A., Garcia, M.U., Di Tommaso, P., and Nahnsen, S. (2020). The nf-core framework for community-curated bioinformatics pipelines. Nat. Biotechnol. *38*, 276–278. https://doi.org/10.1038/s41587-020-0439-x.

STAR★METHODS

KEY RESOURCES TABLE

REAGENT or RESOURCE	SOURCE	IDENTIFIER
**Chemicals, peptides, and recombinant proteins**		
mTeSR™ Plus	Stemcell	Cat#100-0275
TrypLE	Gibco	Cat#12604-021
ROCK inhibitor Y27632	Stemcell	Cat#72304
puromycin	Stemcell	Cat#73342
**Critical commercial assays**		
P3 Primary Cell Nucleofection Kit	Lonza	Cat#V4XP-3024
TIANamp genomic DNA Kit	TIANGEN	DP304-02
Sanger sequencing	Tsingke; Shanghai	https://www.tsingke.com.cn/sanger
STEMdiff neural progenitor medium	Stemcell	Cat#05833
Hieff NGS Ultima Dual-mode mRNA Library Prep Kit	Yeasen	https://seas.ysbuy.com/prod/doc/mainDoc/12308ES-Hieff%20NGS%E2%84%A2%20Ultima%20Dual-mode%20RNA%20Library%20Prep%20Kit-Ver.EN20230327.pdf
**Deposited data**		
Human reference genome T2T-CHM13v1.0	T2T Consortium	GCA_009914755.4
Human population genome from HPRC	Human Pangenome Reference Consortium, Liao et al.[44]	https://data.humanpangenome.org/assemblies
Nonhuman primates T2T genome	Yoo et al.,[38] Zhang et al.[39]	GCA_009914755.4, GCA_028858775.2, GCA_028885625.2, GCA_028885655.2, GCA_029281585.2, GCA_029289425.2, GCA_037993035.1
Neanderthal and Denisovan genomes	Max Planck Institute for Evolutionary Anthropology	https://www.eva.mpg.de/genetics/genome-projects
**Experimental models: Cell lines**		
Han Chinese CN1 cell line	Yang et al.[56]	N/A
**Oligonucleotides**		
L-sgRNA: TGGATCGTAATCACTCGCGG	This paper	N/A
R-sgRNA: GGGACCTCCTCACTTGATTG	This paper	N/A
PCR primer F1: CCCGTGTGAGATGGTCCTTA	This paper	N/A
PCR primer R1: CCACTGCAGTCCGCAGTCTG	This paper	N/A
PCR primer F2: GCAGTATAGTGGTGGCATGC	This paper	N/A
PCR primer R2: TGCAGGTATGAAAATCGCCG	This paper	N/A
**Recombinant DNA**		
BbsI-digested PX459	Addgene	plasmid #48139
**Software and algorithms**		
minimap2	Li et al.[66]	https://github.com/lh3/minimap2
SafFire	N/A	https://mrvollger.github.io/SafFire
RepeatMasker	Dfam Consortium	https://www.repeatmasker.org/
VCFtools	Danecek et al.[67]	https://vcftools.github.io/index.html
MAFFT	Katoh et al.[68]	https://mafft.cbrc.jp/alignment/server/index.html
trimAL	Capella-Gutiérrez et al.[69]	https://github.com/inab/trimal
iqtree	Minh et al.[70]	http://www.iqtree.org/
BEAST	Bouckaert et al.[71]	https://beast.community/
transanno	OKAMURA, Yasunobu	https://github.com/informationsea/transanno
ModDotPlot	Sweeten et al.[72]	https://github.com/marbl/ModDotPlot

(*Continued on next page*)

Continued

REAGENT or RESOURCE	SOURCE	IDENTIFIER
SEDEF	Numanagić et al.[73]	https://github.com/vpc-ccg/sedef
bedtools	Quinlan et al.[74]	https://bedtools.readthedocs.io/en/latest/
circlize	Gu et al.[75]	https://jokergoo.github.io/circlize/
dendextend	Galili et al.[76]	https://github.com/talgalili/dendextend
TreeDist	Smith et al.[77]	https://github.com/ms609/TreeDist
Hum-AS-HMMER for AnVIL	Fedor Ryabov	https://github.com/fedorrik/HumAS-HMMER_for_AnVIL
OWM-SF	Zhang et al.[39]	https://www.nature.com/articles/s41586-025-08596-w#Sec21
Assembly_HSat2and3_v2.pl	Altemose et al.[78]	https://github.com/altemose/chm13_hsat
mrsFAST	Hach et al.[79]	https://github.com/sfu-compbio/mrsfast
RNA-seq pipeline	Harshil Patel et al.[80]	https://nf-co.re/rnaseq/3.19.0/
DESeq2	Love et al.[81]	https://github.com/thelovelab/DESeq2
edgeR	Chen et al.[82]	https://bioconductor.org/packages/devel/bioc/html/edgeR.html
clusterProfiler	Yu et al.[83]	https://github.com/YuLab-SMU/clusterProfiler

METHOD DETAILS

Data resources and comparative analysis

The great ape and macaque T2T genome assemblies are publicly available (Data and code availability). Syntenic relationships among primate chr2/chr2a/chr2b were assessed using minimap2[66] (v2.24) with the following parameters: '-c -x asm20 –secondary = no –eqx -Y -K 8G -s 1000' and visualized using SafFire (https://mrvollger.github.io/SafFire). We identified centromeres, transposons, and subtelomeric satellites based on RepeatMasker (v4.1.4) annotation (https://www.repeatmasker.org/).

Fusion site characterization and population analysis

To precisely define the fusion site, we aligned NHP chr2a and chr2b to human chr2 using minimap2 (v2.24) with the following parameters '-x asm20 -r500,20000 -s 2000 -p 0.01 -N 1000 –cs'. The alignments were visualized using the minimiro (https://github.com/mrvollger/minimiro). We used VCFtools[67] (v0.1.16) to calculate nucleotide diversity (pi) and Tajima's D for population genetic analyses with the following parameters '–window-pi 20000 –window-pi-step 10000' and '–TajimaD 20000'.

SD annotations and phylogenetic analysis

SD annotations of the fusion site in the T2T-CHM13v2.0 were based on the UCSC SEDEF-SD track (https://genome.ucsc.edu/). In this study, we focused on the SDs with an identity of ≥98% and length ≥20 kbp. These SD sequences were aligned to NHP T2T genomes to identify homologous regions using minimap2 (v2.24) with the parameters: '-cx asm20 -p 0.5 –eqx'. Orthologous segments were identified based on flanking region synteny. We aligned all primate homologous SDs with MAFFT[68] (v7.515) and used trimAl[69] (v1.4) to remove the noise sequences with the parameter: '-automated1'. The phylogenetic trees were constructed with IQ-TREE[70] (v2.1.4), and then we used BEAST[71] (v2.6.6) with the HKY model incorporating gamma site, calibrated yule, and relaxed log-normal clock models to infer the split time. Node ages were estimated using log-normal priors (Tables S6D and S6E). We conducted three independent runs for each tree and the results were consistent. Effective sample sizes exceeded 200 for all parameters in all runs.

For ILS analysis, we first truncated the fusion site flanking regions into 500 bp windows. Then, we utilized transanno (v0.4.5) (https://github.com/informationsea/transanno) to align these 500 bp segments to NHP genomes and generated multiple alignments across primate species. We then utilized IQ-TREE[70] (v1.6.12) with HKY model and ete3 python package to analyze phylogeny trees, as described previously.[37]

Subtelomeric repetitive region characterization, methylation analysis, and TE annotation

The pairwise identity heatmaps of each subtelomeric region were generated by ModDotPlot[72] (https://github.com/marbl/ModDotPlot). We used SEDEF[73] (v1.1r35) to annotate SDs. To analyze 5mC methylation levels in pCht regions and SD spacers, we utilized BEDTools[74] (v2.30.0) to calculate mean methylation levels within 1 kbp windows, using a 500 bp step size. Synteny between subtelomeric repetitive regions was defined using minimap2 (v2.24) with the following parameters '-cx asm20 –secondary = no -A1 -B2 -O2,12 -s 1000 -Y -K 8G –eqx'. The syntenic relationship was visualized using R package circlize[75] (v0.4.16). R package dendextend (v1.18.1)[76] and TreeDist (v2.9.1)[77] were utilized to plot the co-phylogeny and to calculate the generalized Robinson–Foulds distance.

Centromere analysis

To analyze the structure of each chromosome, we first ran RepeatMasker (v4.1.4) on all chromosomes and identified the α-satellite-enriched regions as centromeres.[78] Then, we utilized HumAS-HMMER (https://github.com/fedorrik/HumAS-HMMER_for_AnVIL) to classify the suprachromosomal families (SFs) of α-satellites in human, *Pan*, gorilla, and orangutan. For macaque, the OWM-SF annotation tool was used to annotate the SFs.[39] The HOR arrays were characterized using the tool StV (https://github.com/fedorrik/stv). However, for orangutan chr2a and chr2b, the tool encountered difficulties in correctly identifying the HORs, likely due to the complex structure of these acrocentric chromosomes. Thus, we selected the largest continuous regions containing the same SFs as their HORs. We then estimated the frequency of 5mC and CpG methylation within the HOR regions. BEDTools (v2.30.0) was used to count the frequency of methylation within 5 kbp windows and we determined the regions with the minimum frequency and below the lower quartile among the whole HOR as CDRs. Additionally, we used the previous published tool[84] (https://github.com/altemose/chm13_hsat) to identity the HSatII/HSatIII arrays in human and *Pan* peri/centromeric regions (α-satellite-enriched regions and 5 Mbp on the p-arm and q-arm).

We further characterized the human centromere degenerate site through synteny comparisons between *Pan* and human, incorporating RepeatMasker annotations of ‘ALR_Alpha.’ Breakpoints between chimpanzees and humans were identified through alignment and analysis of specific SF organizations. ModDotPlot (https://github.com/marbl/ModDotPlot) was used to generate heatmaps for chimpanzee chr2b with a window size of 5,000 bp and for the human centromere degenerate site with a window size of 200 bp.

To assess the diversity of centromere degeneration across human populations, we first confirmed the presence of this site in all human genomes using minimap2 (v2.24) with the parameters ‘-cx asm20 –secondary = no -s 2500’. We then extracted the targeted regions from each assembly from HPRC data and ran RepeatMasker (v4.1.4) on these regions. We defined three satellite arrays—cenD_1, cenD_2, and cenD_3—with subtypes determined by length variations.

To confirm the fusion occurred in archaic humans, we randomly selected two individuals from the five structural haplotypes (total 10 individuals) and five NHP genomes to run mrsFAST[79] (v3.4.2) for identifying singly unique nucleotide *k*-mers (SUNKs) in modern humans. Subsequently, we checked the counts of the SUNKs in archaic human genomes.

To compare the methylation frequencies among human chr2, α-satellite arrays at the degenerate centromeric site, and NHP centromeres, we chunk the HORs (excluding CDRs; for macaques, we used the whole α-satellite region, excluding CDRs) into 17.1 kbp windows by BEDTools (v2.30.0) with parameters: ‘-w 171000 -s 8550’ and calculate the frequencies within windows. Visualizations were generated using ggplot2.

Fusion site KO experiments

The design of optimal sgRNA pairs to target sites was performed using the online CRISPR design tool (http://crispor.gi.ucsc.edu/). The complementary oligonucleotide pairs of L-sgRNA and R-sgRNA were annealed at 95°C for 5 min, with ramp-down to 25°C to generate the double-stranded DNA (dsDNA) fragment, before ligation into BbsI-digested PX459 (plasmid #48139, Addgene).

Wild-type induced pluripotent stem cells (CN1) or fusion site depletion induced pluripotent stem cells were maintained in mTeSR Plus (Stemcell). Cells were dissociated into single-cell suspensions using TrypLE (Gibco) and electroporated using the Lonza Nucleofector 4D X Unit (program CA137) and the P3 Primary Cell Nucleofection Kit (V4XP-3024). The following conditions were used: 5–6 $\times$ 10^5 cells/mL, 10 μg sgRNA (two sgRNAs mixed at a 1:1 ratio). The control groups were electroporated with scramble sgRNAs. After electroporation, iPSCs were resuspended in mTeSR Plus supplemented with 10 μM ROCK inhibitor Y27632 and plated onto 12-well culture plates within 24 h. At 48 h post-electroporation, the culture medium was replaced with fresh mTeSR Plus containing puromycin (1μg/mL) for 48h of selection. Cells were subsequently maintained in standard mTeSR Plus medium until colony formation.

We selected three independent monoclonal iPSC-CN1 cell lines for the knockout (KO, n = 3) and control (CTRL, n = 3) condition. For isolation of KO or control monoclonal clones, selected colonies were dissociated into single-cell suspensions. Individual cell was manually transferred using a mouth pipette under a stereomicroscope and seeded into 96-well plates. When a single cell grows into a clonal colony, we extract the genomic DNA from the cells with the TIANamp genomic DNA Kit (DP304-02, Tiangen) according to the manufacturer’s instructions. Genome deletion was detected by PCR-amplification of gDNA using a primer pair flanking the deletion. The primers used for PCR screening are listed in Table S5A. Genomic PCR products were detected by agarose gel electrophoresis and Sanger sequencing (Shanghai, Tsingke).

iPSCs differentiation into neural progenitor cells

Neural Progenitor Cells (NPCs) were generated from control iPSCs or KO iPSCs using the STEMdiff Neural System (Stemcell). Briefly, Cells were harvested using TrypLE and resuspension in STEMdiff Neural Induction Medium + SMADi +10 μM Y-27632 as single cells. Add cell suspension (2 $\times$ 10^6 cells/well) to a single well of the matrix-coated 6-well plate and then perform a daily full-medium change with warm (37°C) medium until cultures are ready to be passaged. At passage 3, NPCs were expanded in STEMdiff Neural Progenitor Medium.

RNA preparation and RNA-seq data analysis

Total RNA was isolated from each replicate of the KO cell lines and the control cell lines using Hieff NGS Ultima Dual-mode mRNA Library Prep Kit, following the manufacturer's instructions. We utilized RNA-seq pipeline[80] from the nf-core[85] community with default parameters to align and quantify the RNA-seq data. Differential expression analysis was conducted using the R package DESeq2[81] and edgeR.[82] We identified differentially expressed genes (DEGs) with following criteria: false discovery rate (FDR) < 0.05 and a $\log_2$ fold change >2. Because edgeR and DESeq2 produced similar results, we used the results by edgeR to do the further analysis. We performed Gene Ontology (GO) enrichment analysis using clusterProfiler.[83]

Cell Genomics, Volume *6*

Supplemental information

Incomplete lineage sorting of segmental duplications defines the human chromosome 2 fusion site early during African great ape speciation

Zikun Yang, Lu Zhang, Xinrui Jiang, Xiangyu Yang, Kaiyue Ma, DongAhn Yoo, Yong Lu, Shilong Zhang, Jieyi Chen, Yanhong Nie, Xinyan Bian, Junmin Han, Lianting Fu, Juan Zhang, Mario Ventura, Guojie Zhang, Qiang Sun, Evan E. Eichler, and Yafei Mao

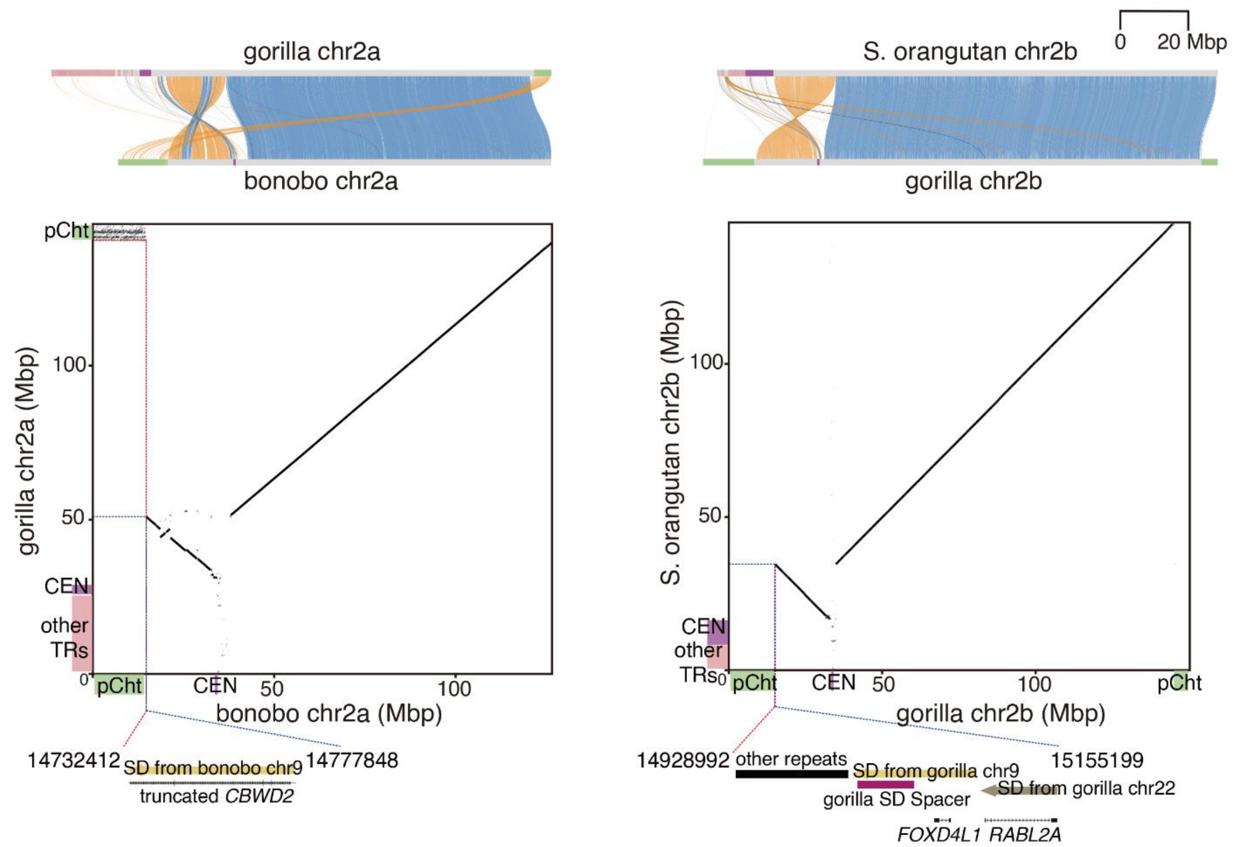

Figure S1. The syntenic comparison of primate chromosome 2 pericentric inversions, related to Figure 1. The red dotted line indicates the start of the pericentric breakpoints, while the blue dotted line marks their end. The segmental duplications (SDs) associated with breakpoints are shown on the bottom. S. orangutan represents Sumatran orangutan.

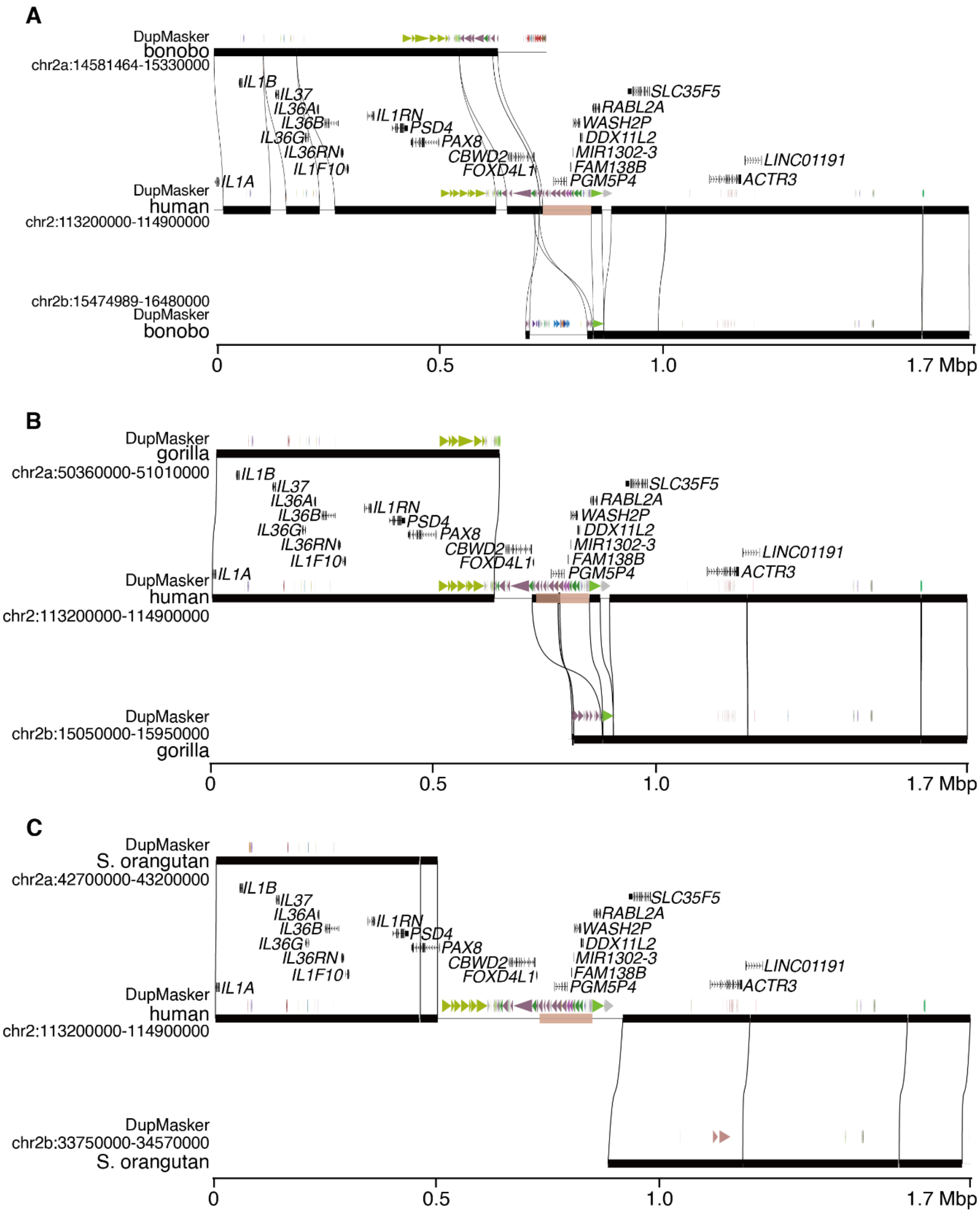

Figure S2. The comparative analysis of primate chromosome 2 and the human fusion site, related to Figure 1. Minimiro comparisons between the human fusion site and those in bonobo (A), gorilla (B), and S. orangutan (C). Gene and SD annotations are displayed on each chromosome.

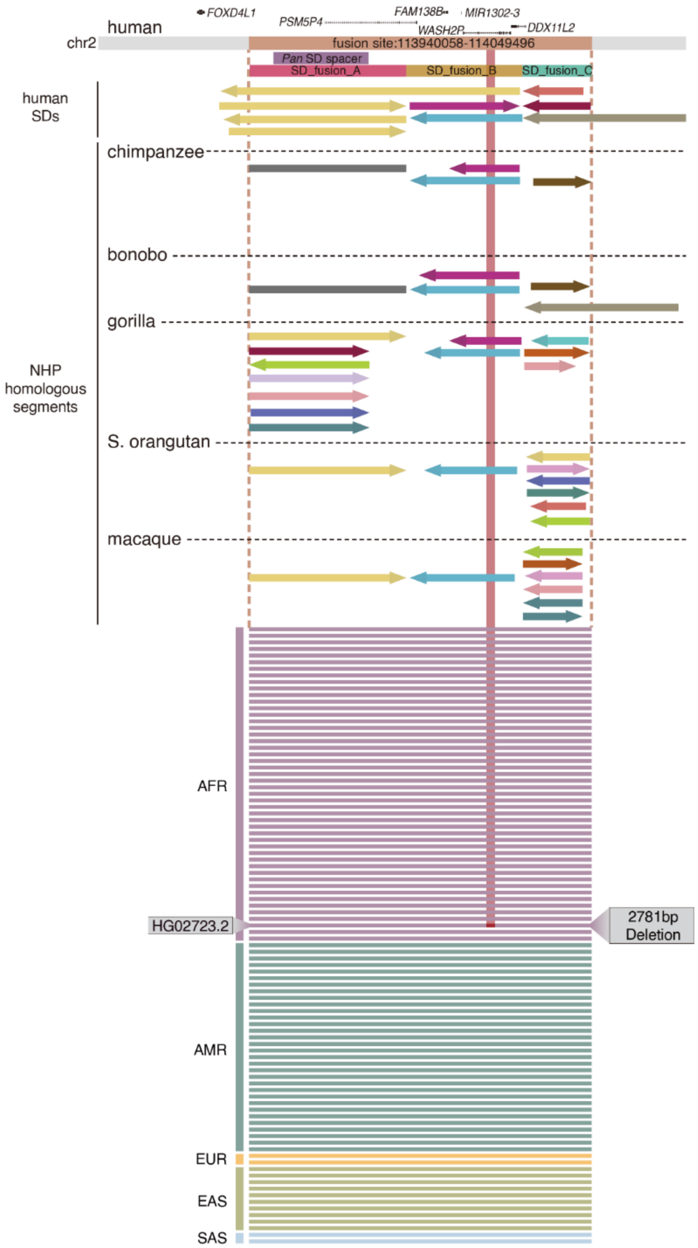

Figure S3. The structure of the fusion site in Human Pangenome Reference Consortium (HPRC) samples, related to Figure 2. The top panel shows the structure of the fusion site (related to Figure S5), while the bottom panel shows the schematic genomic structure of each HPRC sample. Structural variants (SVs) longer than 1 kbp are shown in red boxes. Only a 2,781 bp deletion in HG02723 haplotype 2 is identified, suggesting a highly conserved structure of the fusion sites in human populations.

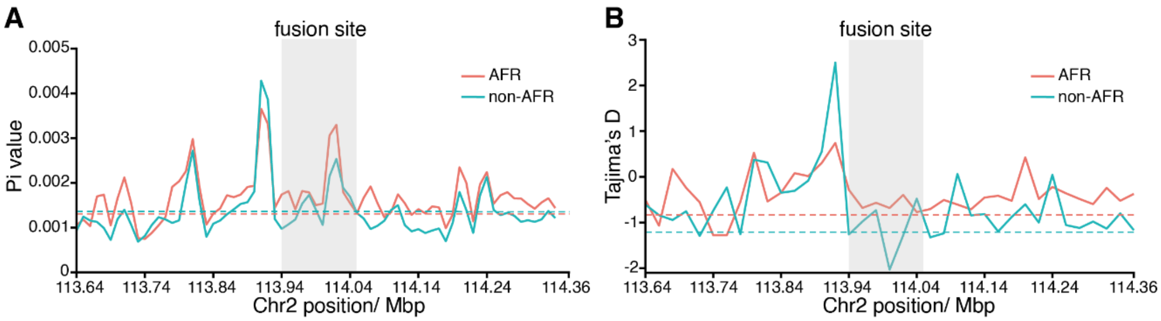

Figure S4. Population genetic statistics of the human fusion site, related to Figure 2. The π diversity (A) and Tajima's D (B) are estimated using long-read human genome assemblies of African (red, n=102) and non-African (blue, n=436) populations. The dashed lines show the average values for human chromosome 2.

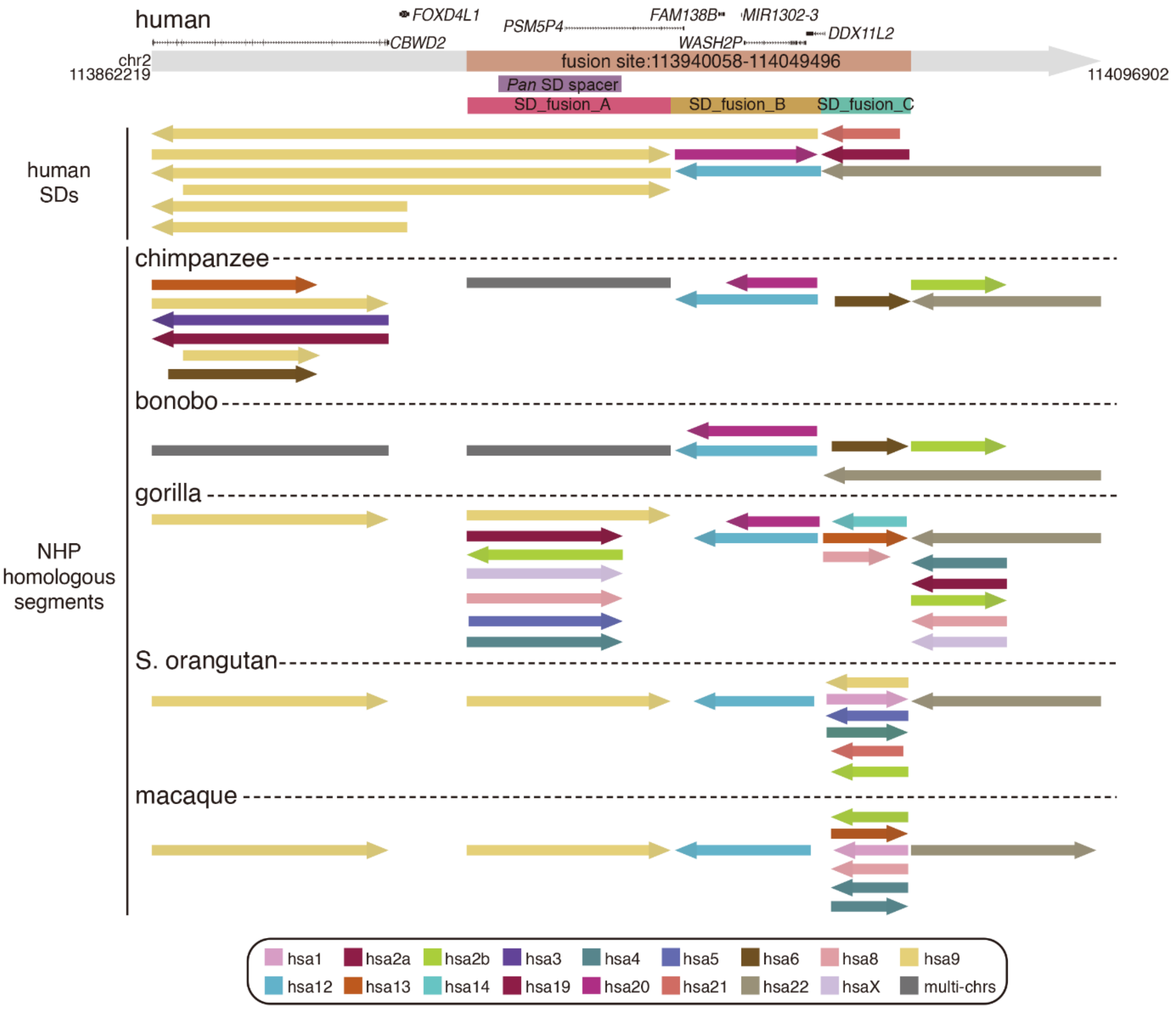

Figure S5. The human fusion site comprises distinct SDs, related to Figure 2. A human genomic segment (chr2:113,862,219-114,096,902) is shown in grey, with the human fusion site (chr2:113,940,058-114,049,496) highlighted in amber. Homologous segments in nonhuman primates (chimpanzee, bonobo, gorilla, orangutan, and macaque) are also depicted in corresponding order. Dark grey blocks represent multi-chromosomes in the same region; see Table S3.

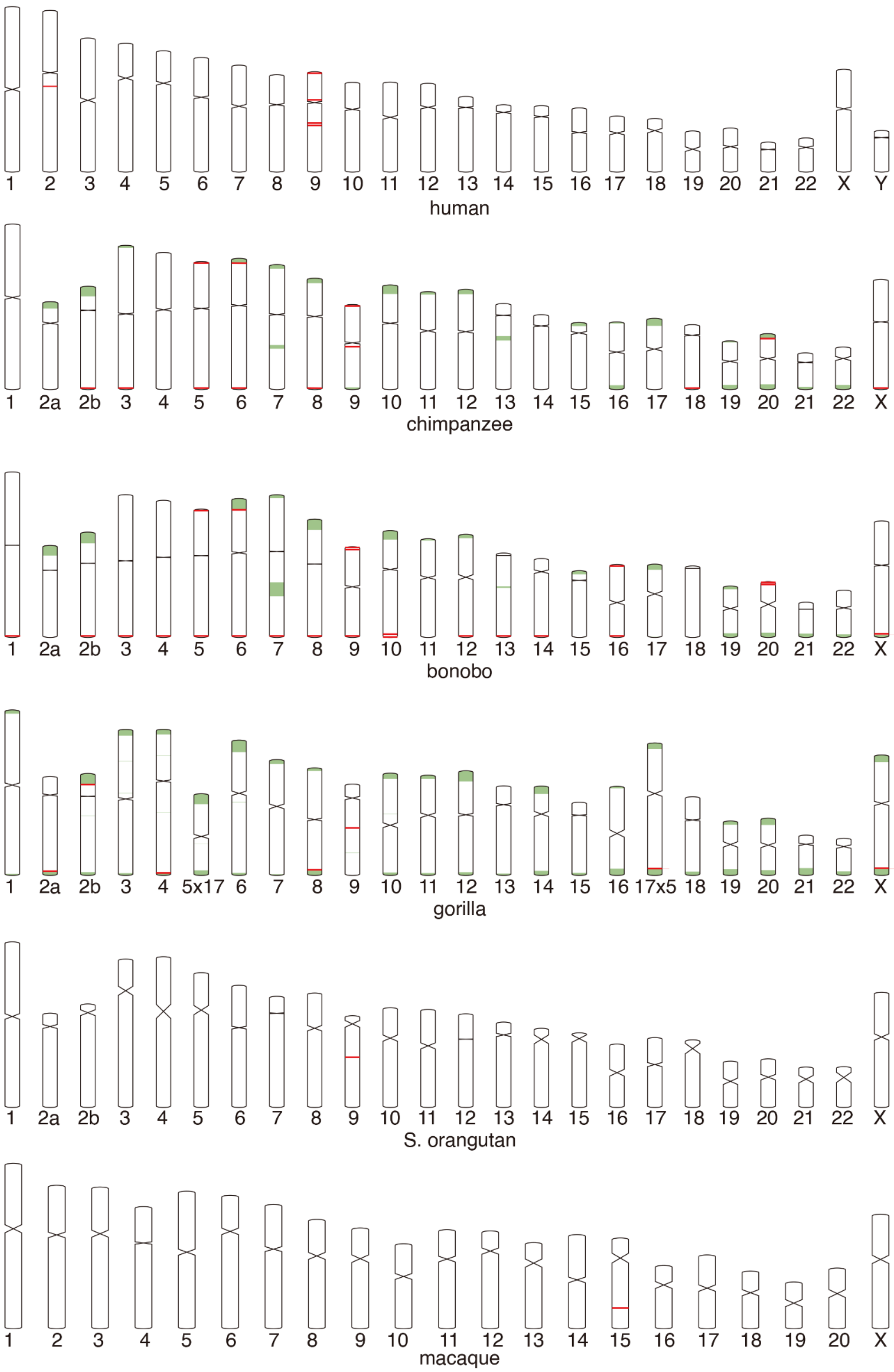

Figure S6. The ideogram of homologous segments in human and NHPs for SD_fusion_A (chr2: 113,940,058-113,990,477), related to Figure 2. Red blocks represent homologous segments and green blocks represent pCht.

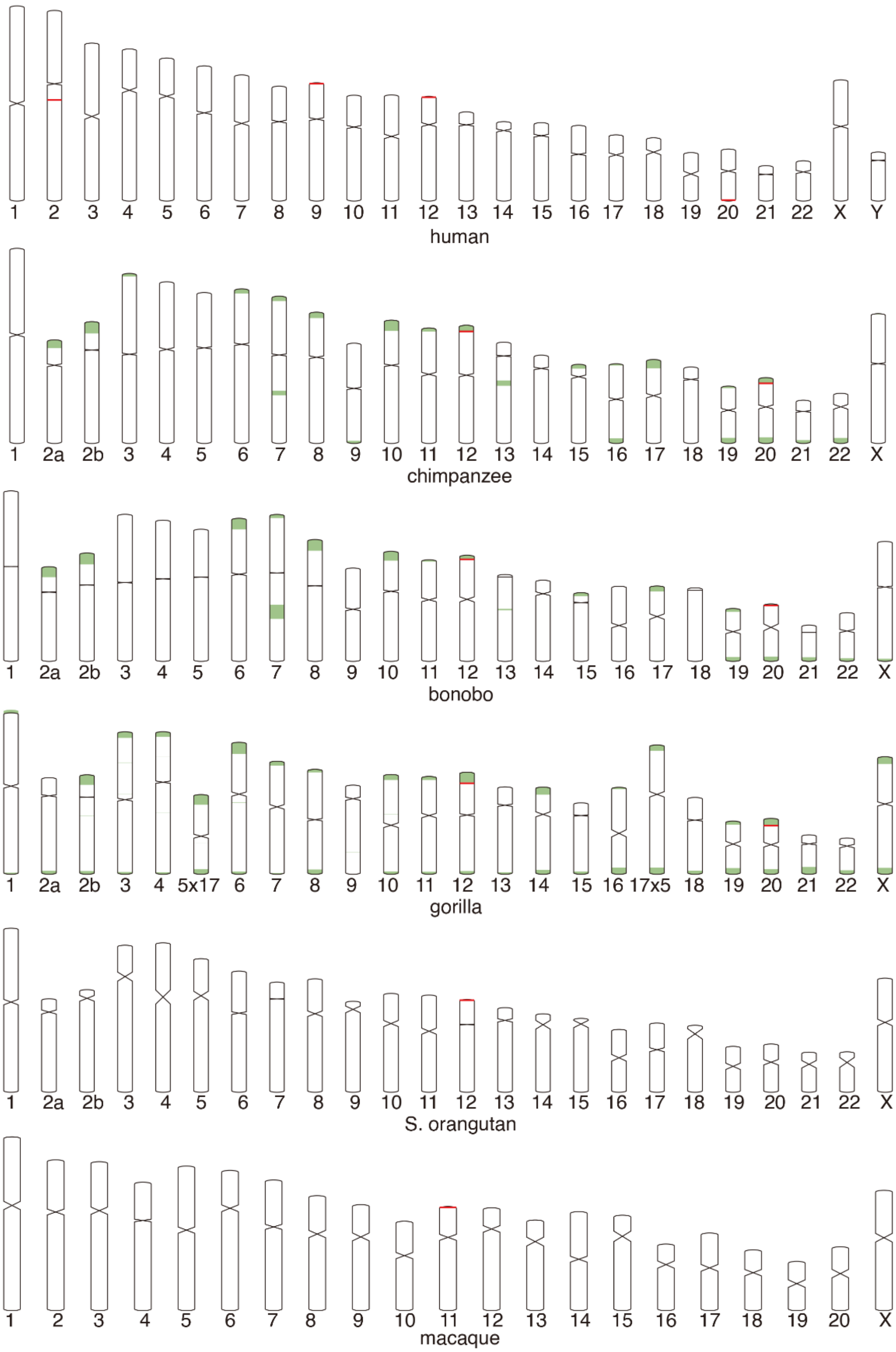

Figure S7. The ideogram of homologous segments in human and NHPs for SD_fusion_B (chr2:113,991,553-114,027,657), related to Figure 2. Red blocks represent homologous segments and green blocks represent pCht.

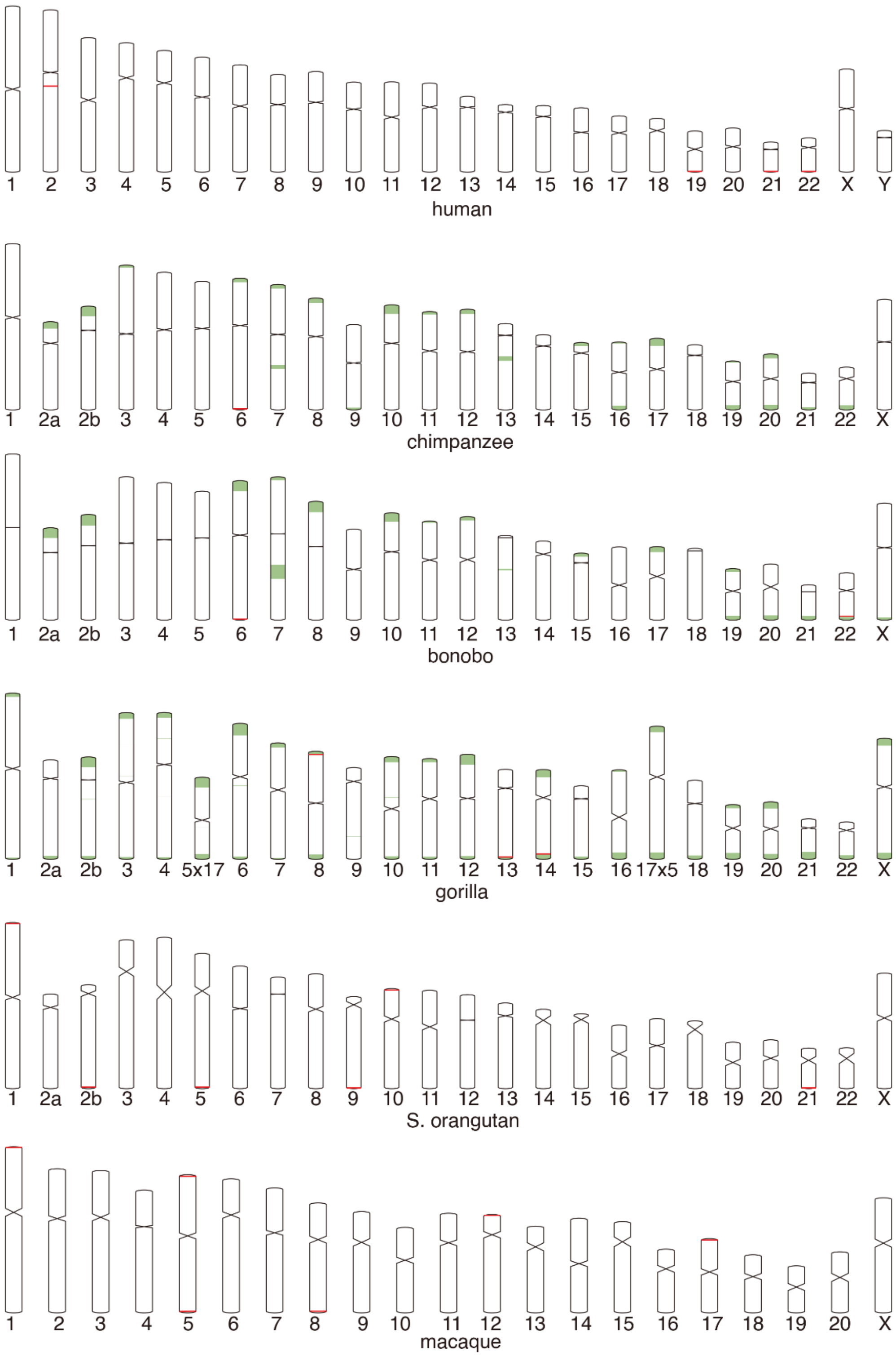

Figure S8. The ideogram of homologous segments in human and NHPs for SD_fusion_C (chr2:114,027,659-114,049,946), related to Figure 2. Red blocks represent homologous segments and green blocks represent pCht.

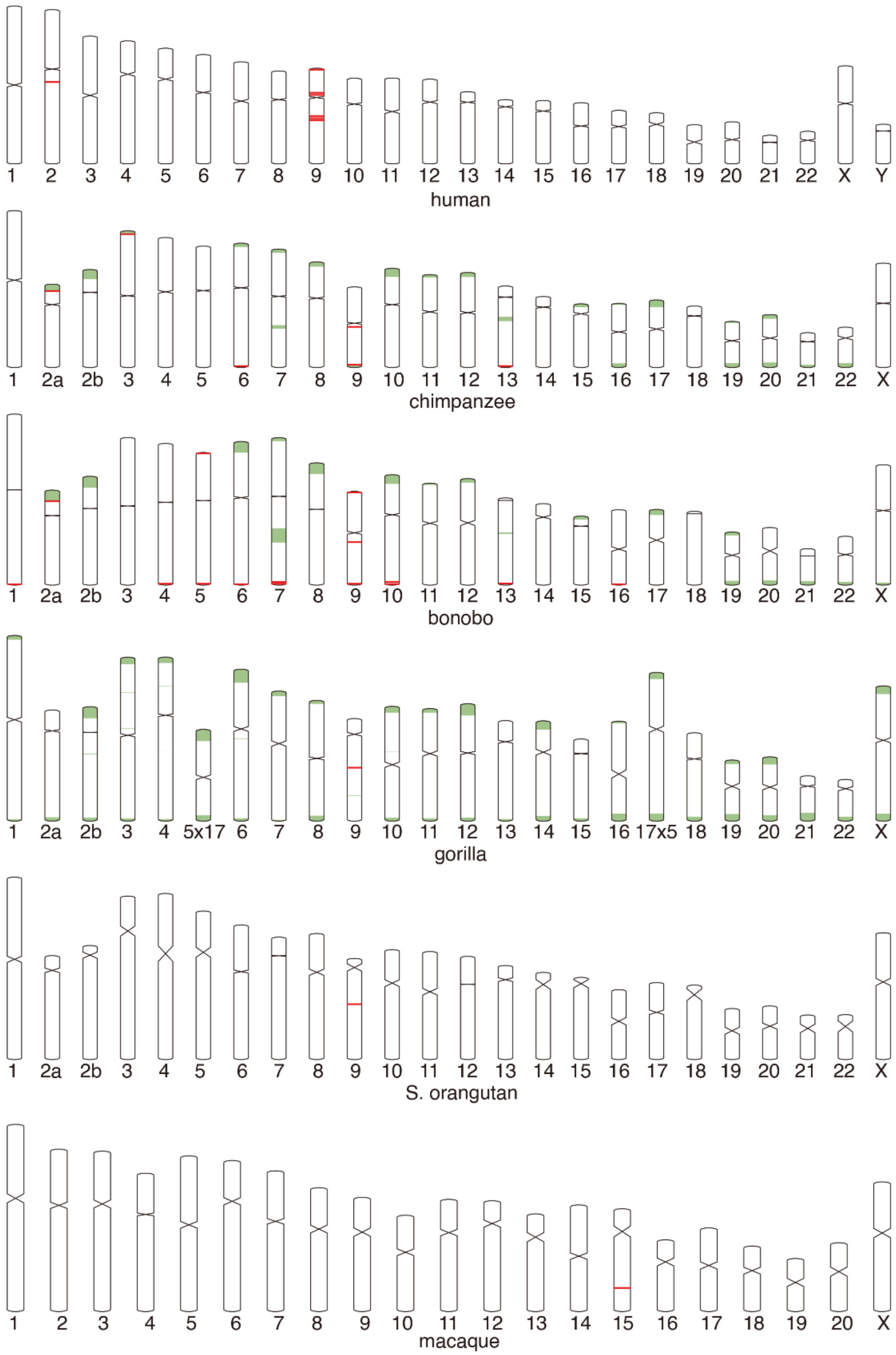

Figure S9. The ideogram of homologous segments in human and NHPs for the left flanking region 1 of the fusion site (chr2: 113,837,958-113,940,058), related to Figure 2. Red blocks represent homologous segments and green blocks represent pCht.

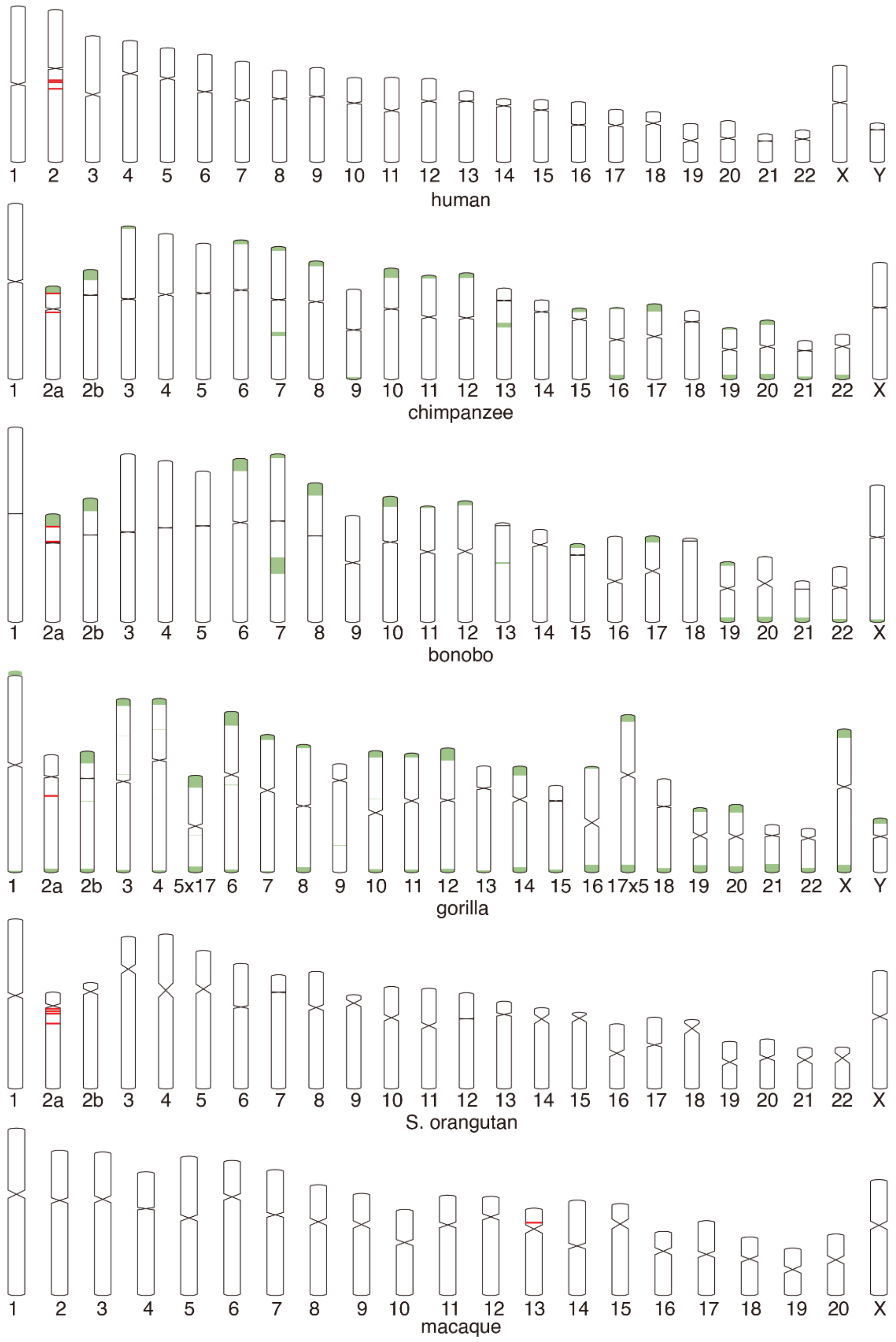

Figure S10. The ideogram of homologous segments in human and NHPs for the left flanking region 2 of the fusion site (chr2: 113,710,520-113,837,957), related to Figure 2. Red blocks represent homologous segments and green blocks represent pCht.

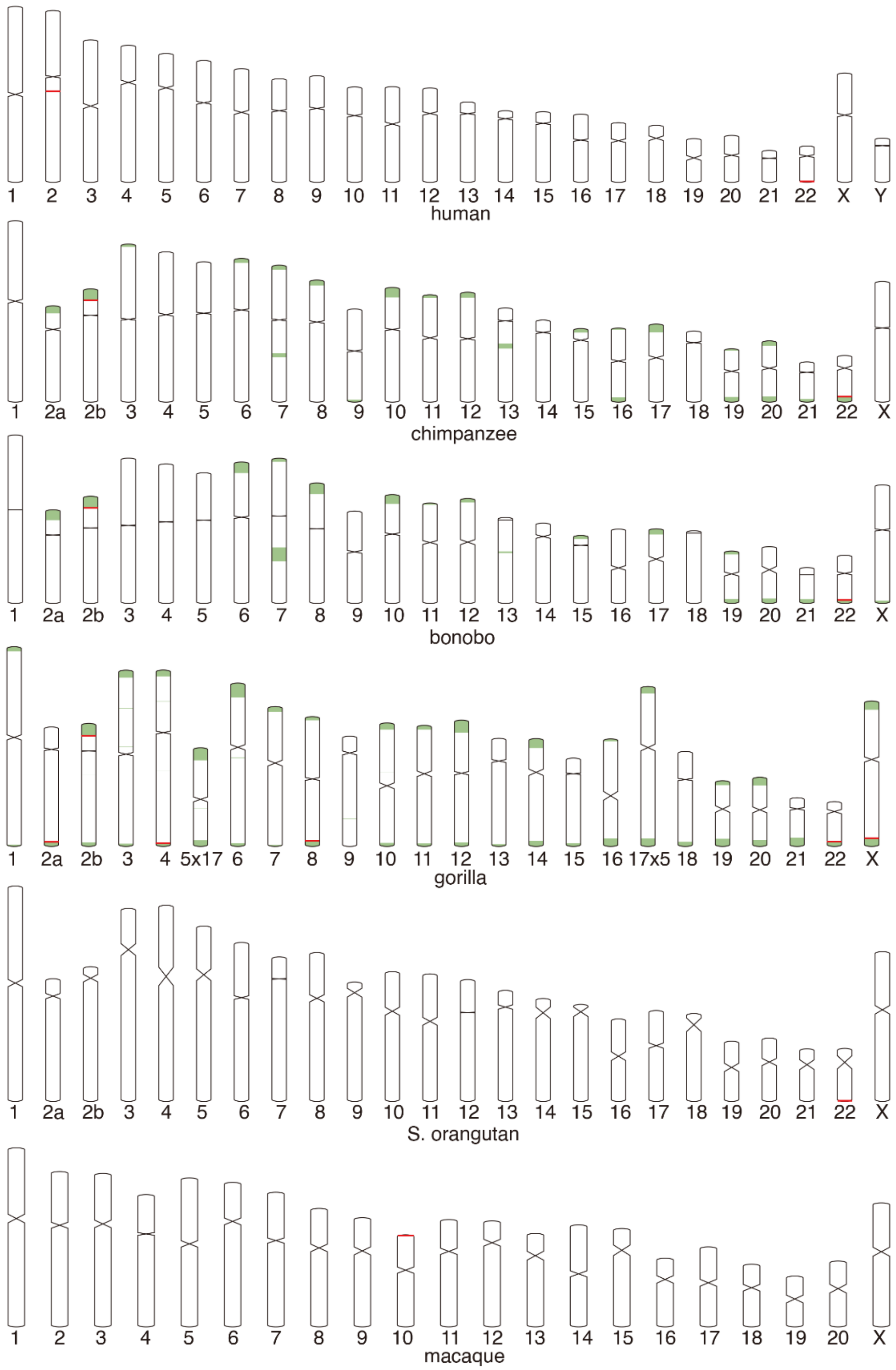

Figure S11. The ideogram of homologous segments in human and NHPs for the right flanking region 1 of the fusion site (chr2: 113,710,520-113,862,218), related to Figure 2. Red blocks represent homologous segments and green blocks represent pCht.

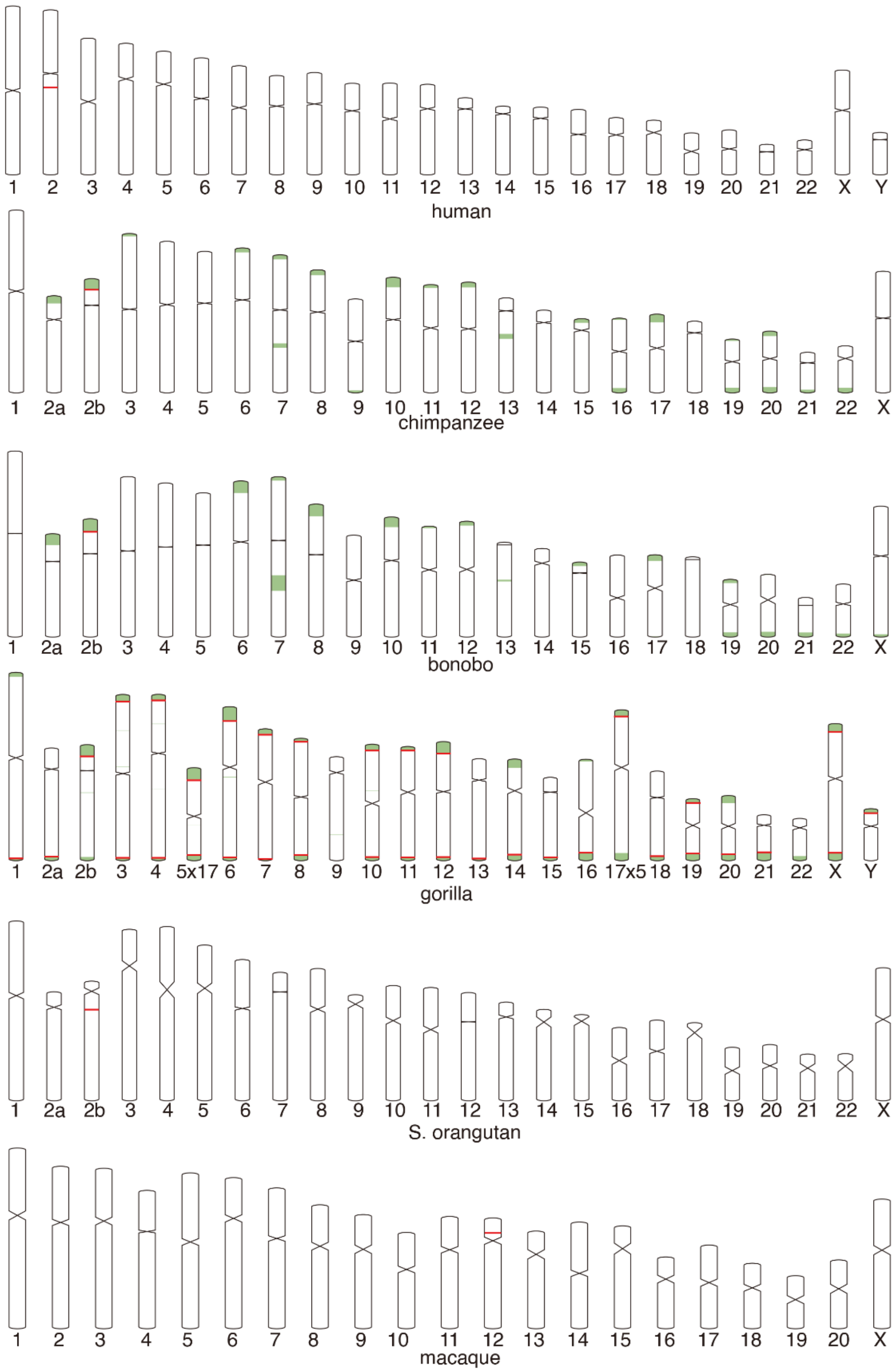

Figure S12. The ideogram of homologous segments in human and NHPs for the right flanking region 2 of the fusion site (chr2: 114,096,902-114,165,116), related to Figure 2. Red blocks represent homologous segments and green blocks represent pCht.

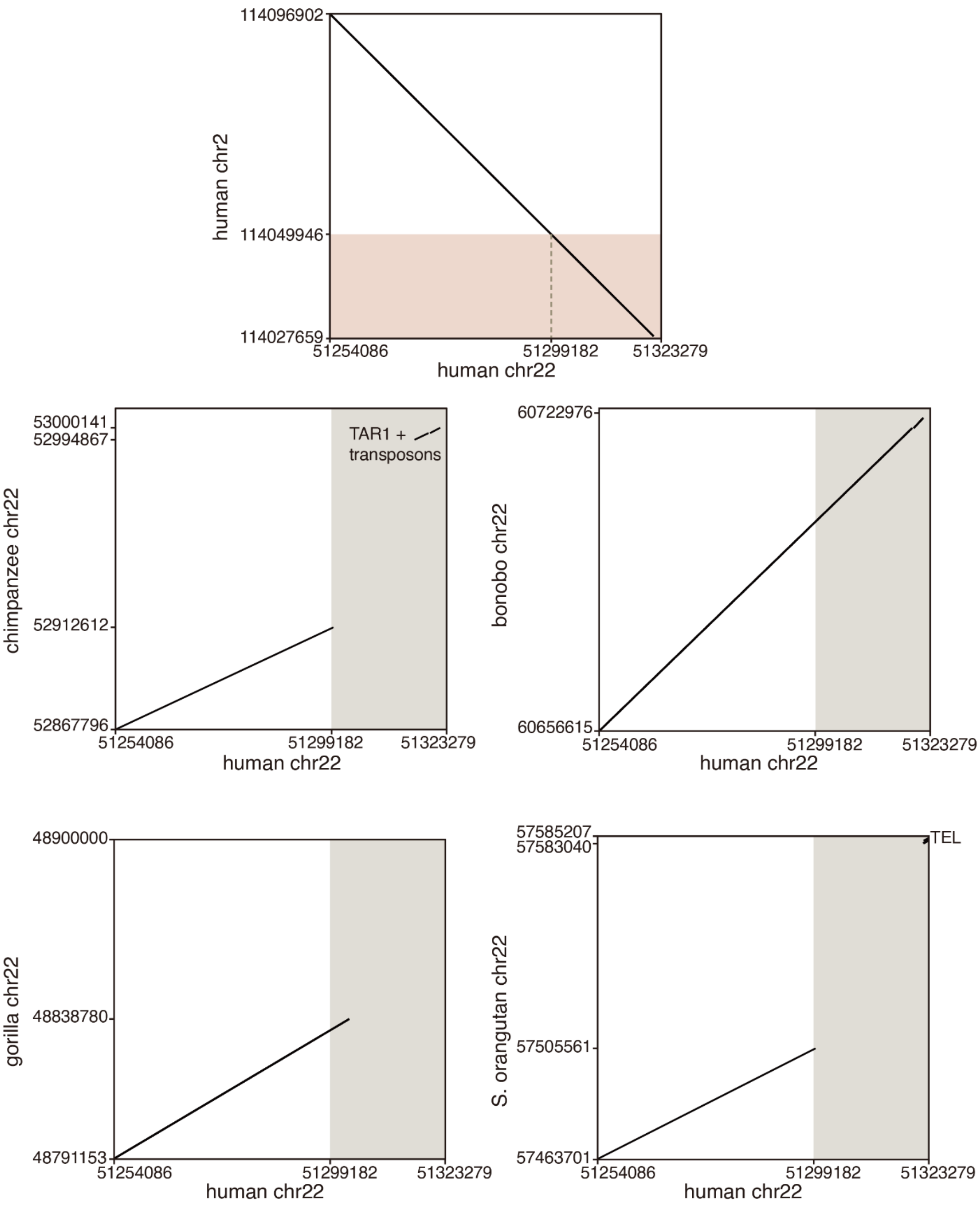

Figure S13. The identity dot plots of SDs among human SD_fusion_C and its flanking region, human chr22 segment, and NHP chr22 segment, related to Figure 2. The dot plot on the top shows the syntenic comparison between human SD_fusion_C (amber block) with its flanking region and human chr22 segment. The other dot plots show the human chr22 segment and its orthologous segments on each NHP.

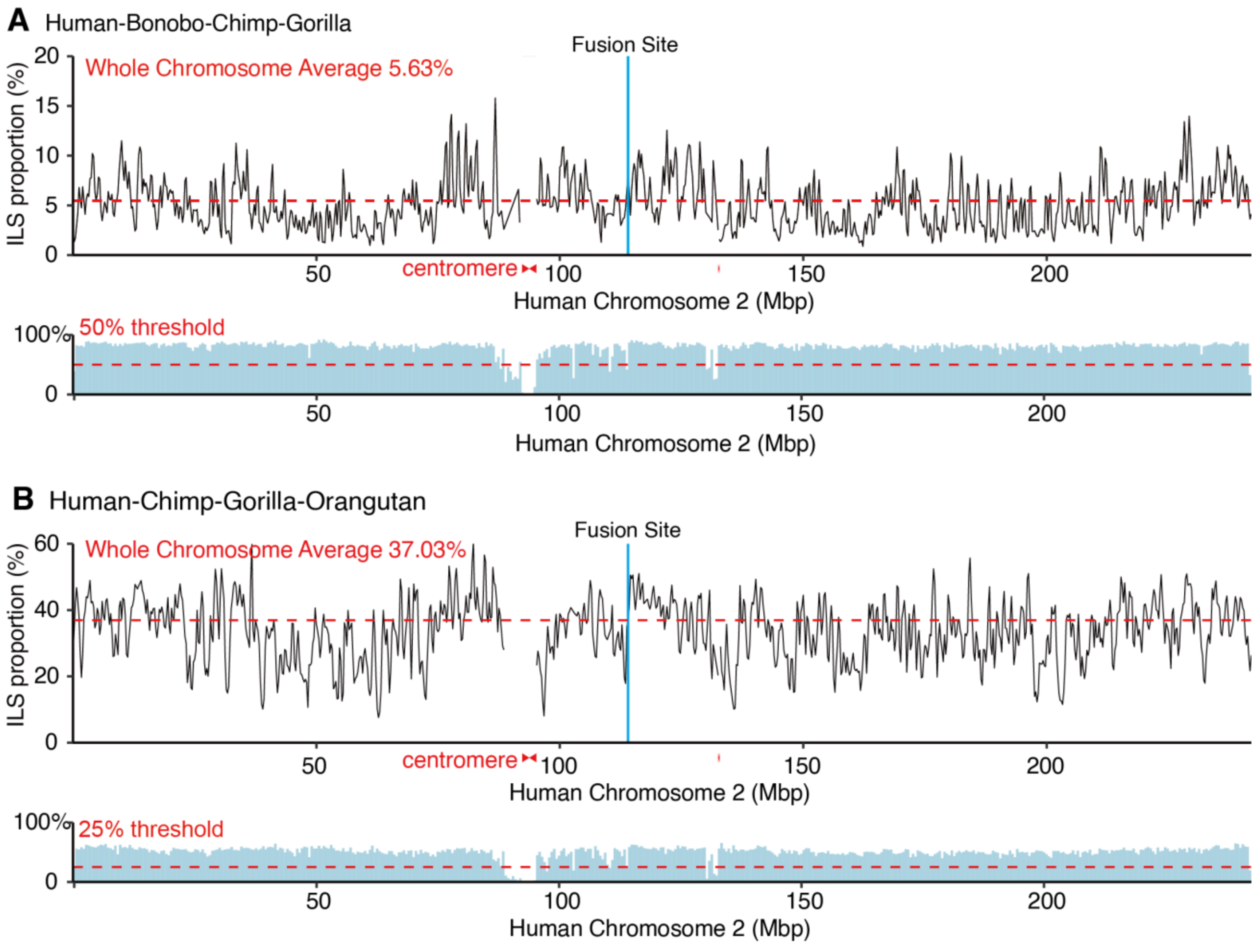

Figure S14. Incomplete lineage sorting (ILS) intensity on the whole chromosome 2, related to Figure 2. (A) ILS signal distribution for the entire chromosome 2 with human, chimpanzee, bonobo, and gorilla genomes. (B) ILS signal distribution for the entire chromosome 2 with human, chimpanzee, gorilla, and orangutan genomes. Phylogeny trees are constructed with 500 bp window sequences and the mean proportion of ILS is calculated with 500 kbp windows. For each panel, the upper plot shows the mean ILS proportion, and the bottom plot shows the effective data coverage in each window, indicating the mapping quality and reliability.

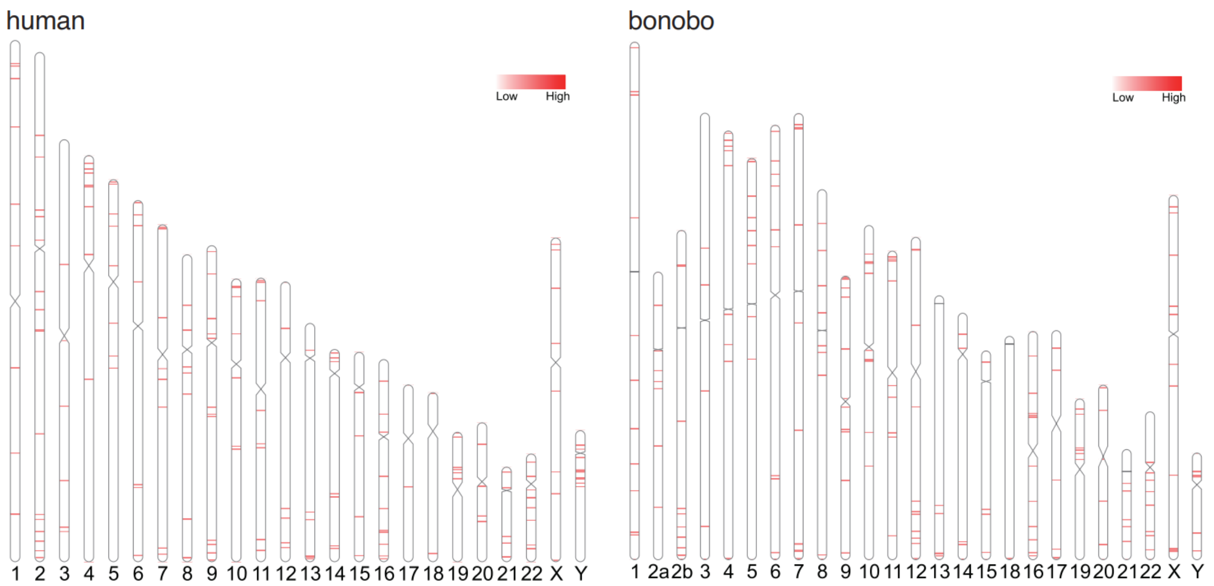

Figure S15. The telomeric sequences at the chromosomal interstitial sites in humans and bonobo, related to Figure 2. The red blocks represent the telomeric repeats.

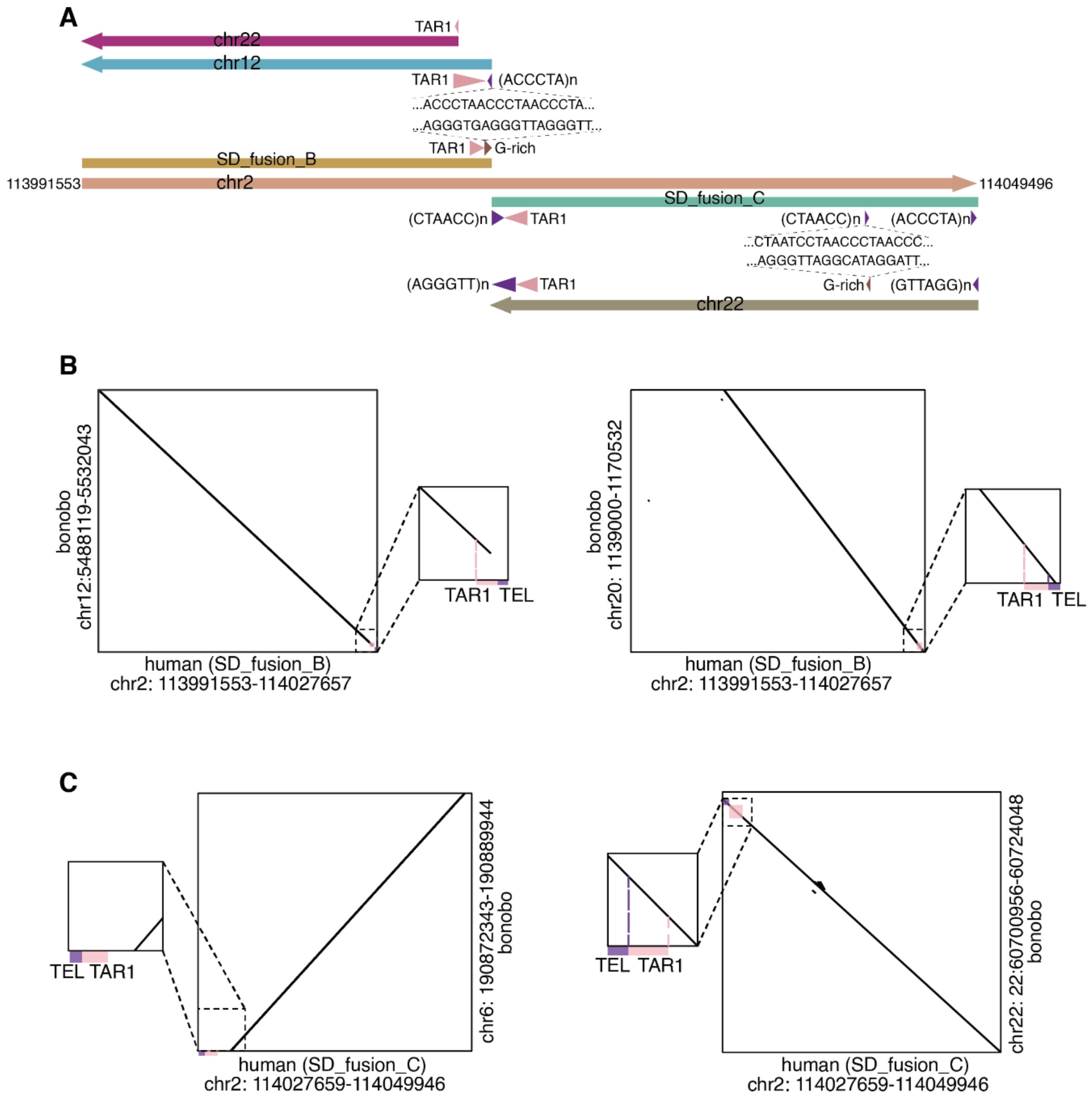

Figure S16. Telomeric sequences at the fusion site and associated SDs, related to Figure 2. (A) The human genomic segment (chr2:113,991,553-114,049,496) at the fusion site is shown in amber. SD from human chr12 is represented in blue and SD from human chr22 is depicted in tan. The tracks of repeat sequences were annotated by RepeatMasker. The G-rich regions show the similar telomeric sequences. Genomic regions in the bonobo that share homology with SD_fusion_B (B) and SD_fusion_C (C) contain similar telomeric sequences.

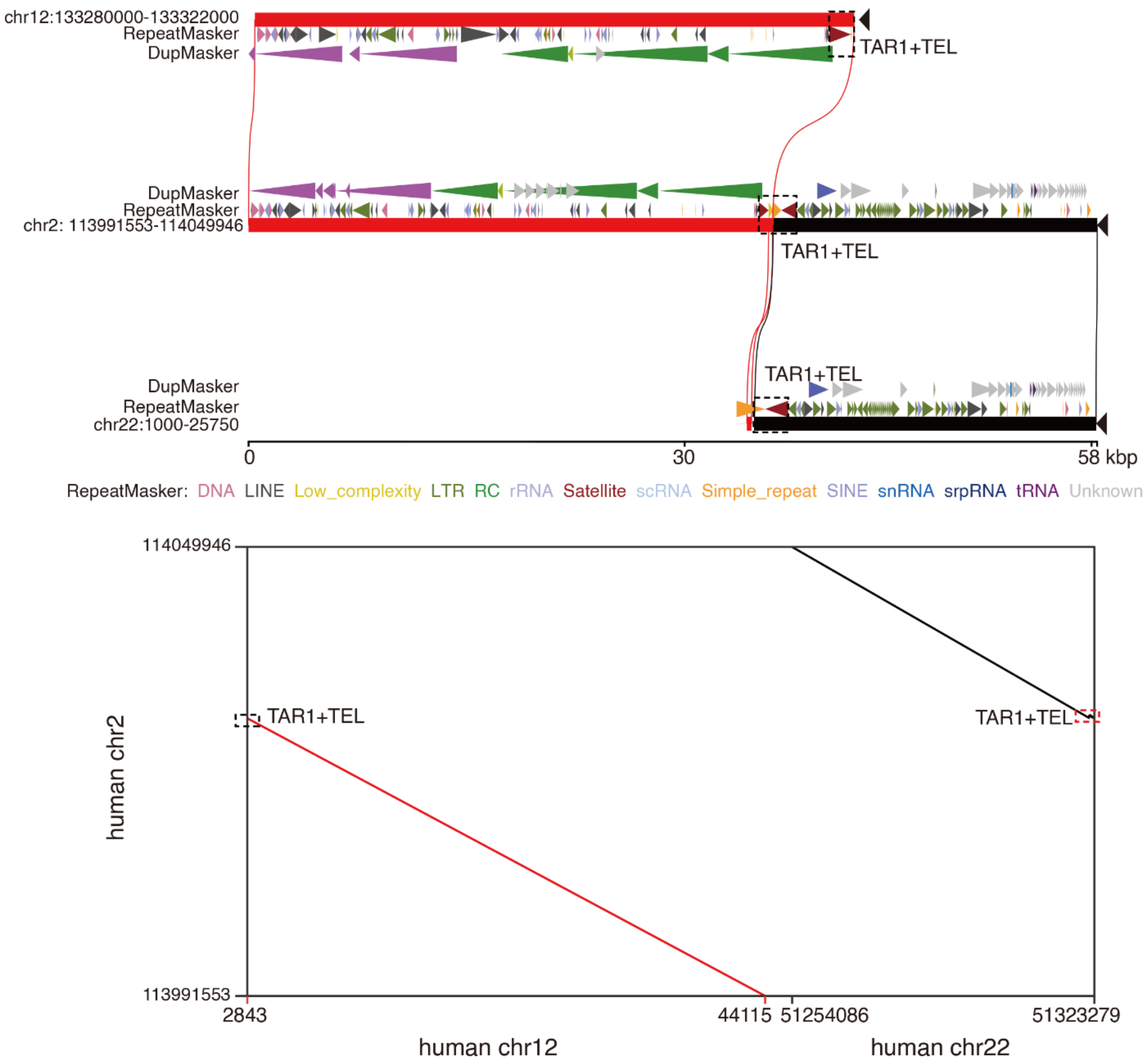

Figure S17. The orientation of telomeric-associated repeat (TAR1) and telomeric sequences at the fusion site in chr2 and SDs from chr12 and chr22, related to Figure 2. The synteny plot shows regions from chromosome 12 (red) and chromosome 22 (black) as reverse complement orientation, while the dot plot reveals that the TAR1 retains the same orientation as other SDs.

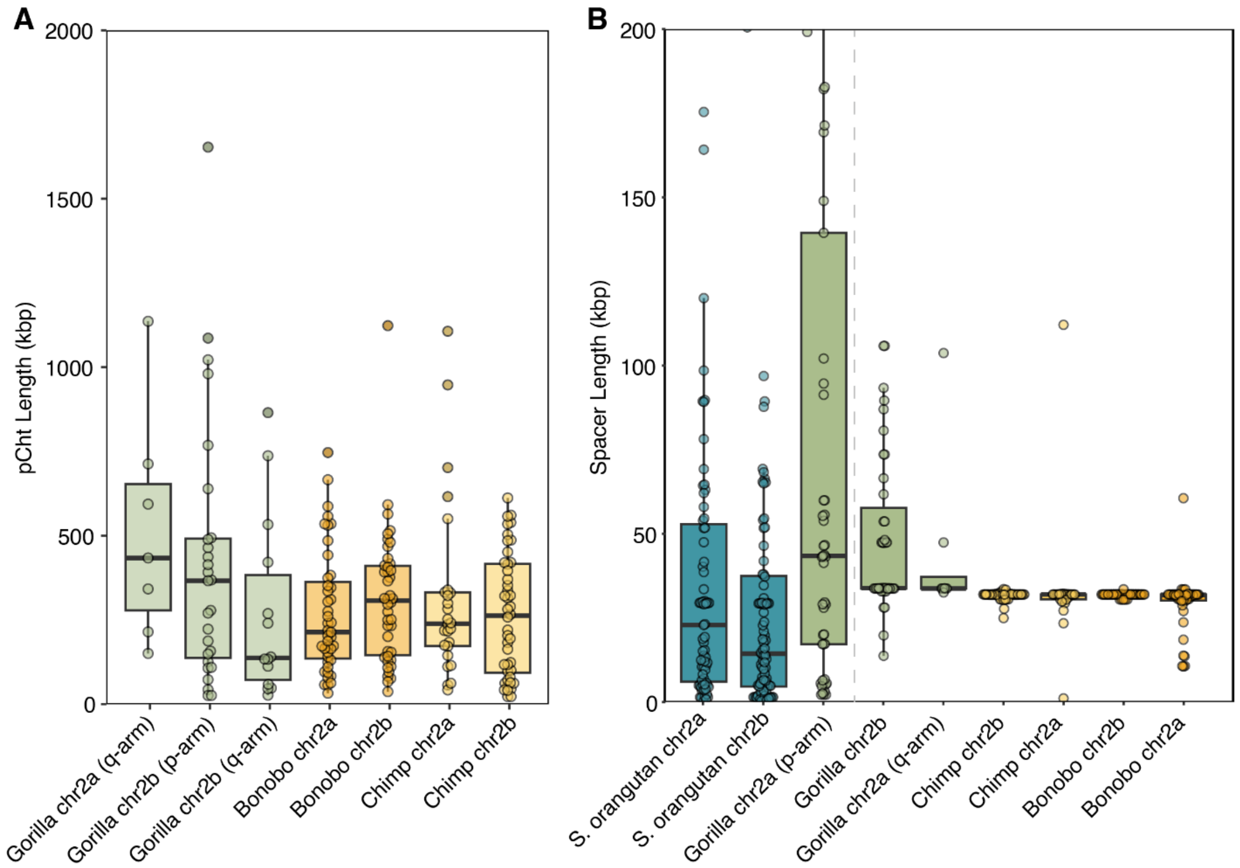

Figure S18. Distribution of pCht length and spacer length, related to Figure 3. (A) The pCht sequence lengths are determined by the distances between adjacent SD spacers. (B) The boxplots of spacer length highlight a normal distribution of lengths for *Pan* and gorilla spacers (excluding gorilla chr2a p-arm).

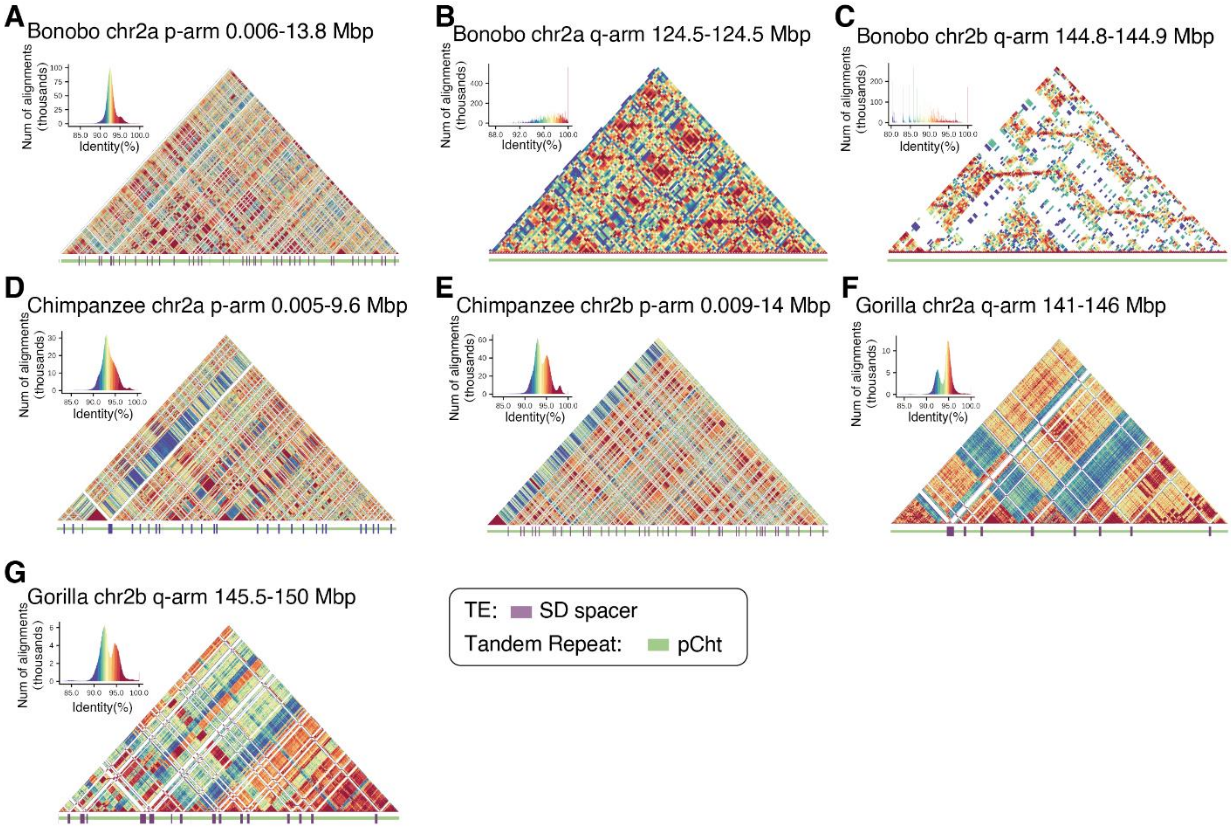

Figure S19. Identity heatmaps and spacer diagrams of subtelomeric repetitive regions in *Pan* and gorilla, related to Figure 3. The thicker and thinner rectangles below heatmaps stand for the transposable elements (TEs) track and tandem repeats (TRs) track, respectively. The species information and approximate coordinates of the other heatmaps and corresponding TE/TR tracks are at the top of the panels.

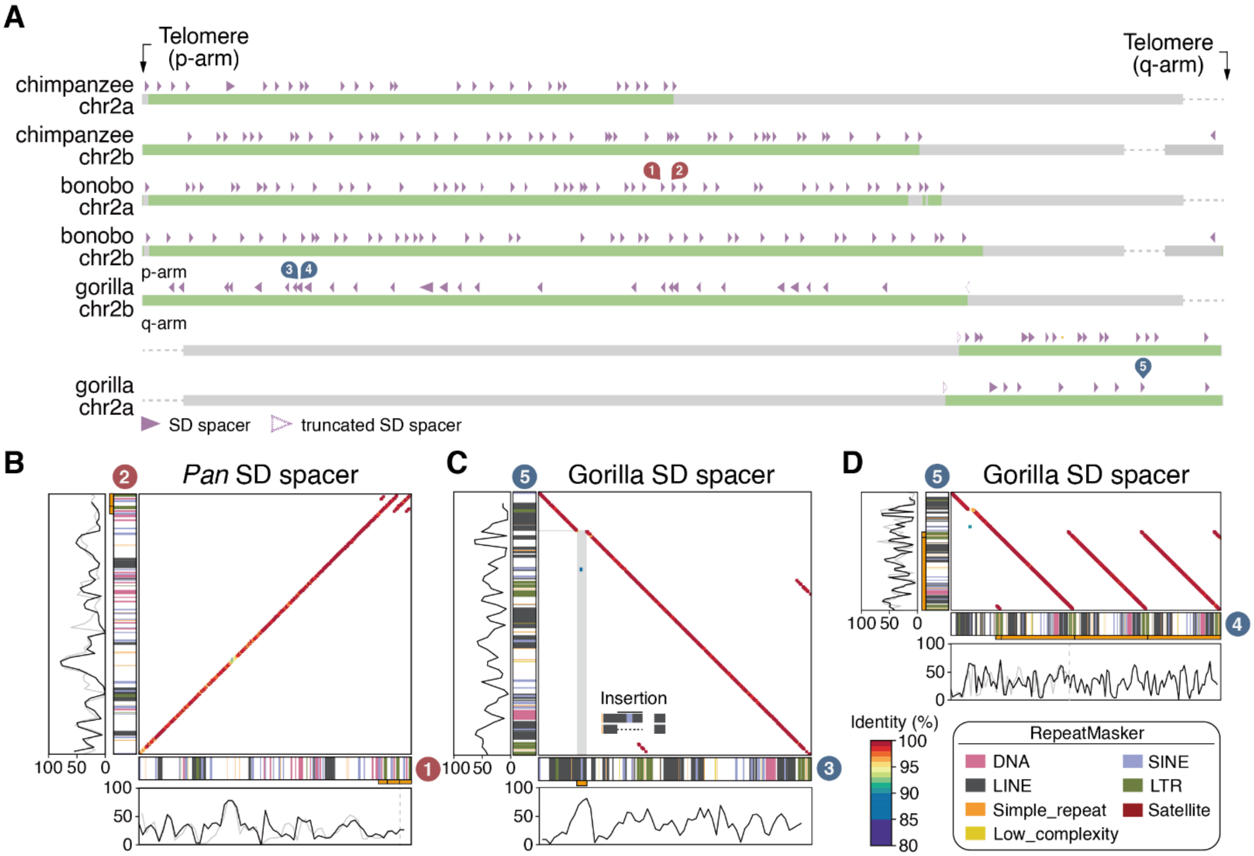

Figure S20. Diagram for spacer distribution and synteny among SD spacers, related to Figure 3. (A) The diagram illustrates the genomic structures of SD spacers (purple) within the pCht regions (light green) of gorilla and *Pan* species. Internal SVs and tandem duplications lead to the variable lengths of SD spacers in *Pan* (B) and gorilla (C-D). In panel (C), a 1.2 kbp LINE-SINE-LINE insertion is identified within an SD spacer in gorilla.

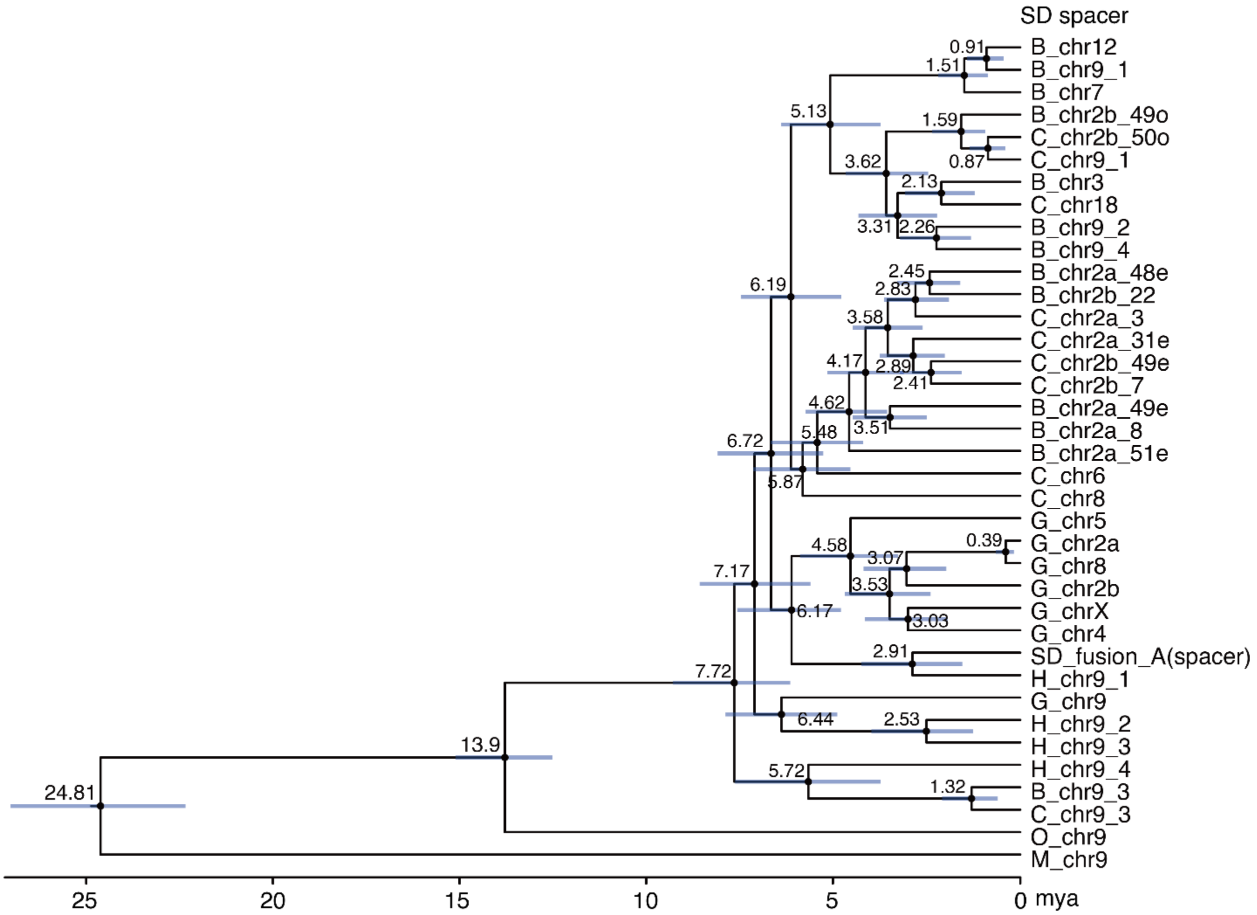

Figure S21. Phylogeny tree of *Pan* SD spacer, related to Figure 3. H, B, C, G, O, and M stand for human, bonobo, chimpanzee, gorilla, Sumatran orangutan, and macaque, respectively. Spacers on chr2 with ‘o’ and ‘e’ markers are out and end of subtelomeric repetitive regions, respectively. The other SD spacers on chr2 are in the subtelomeric repetitive regions.

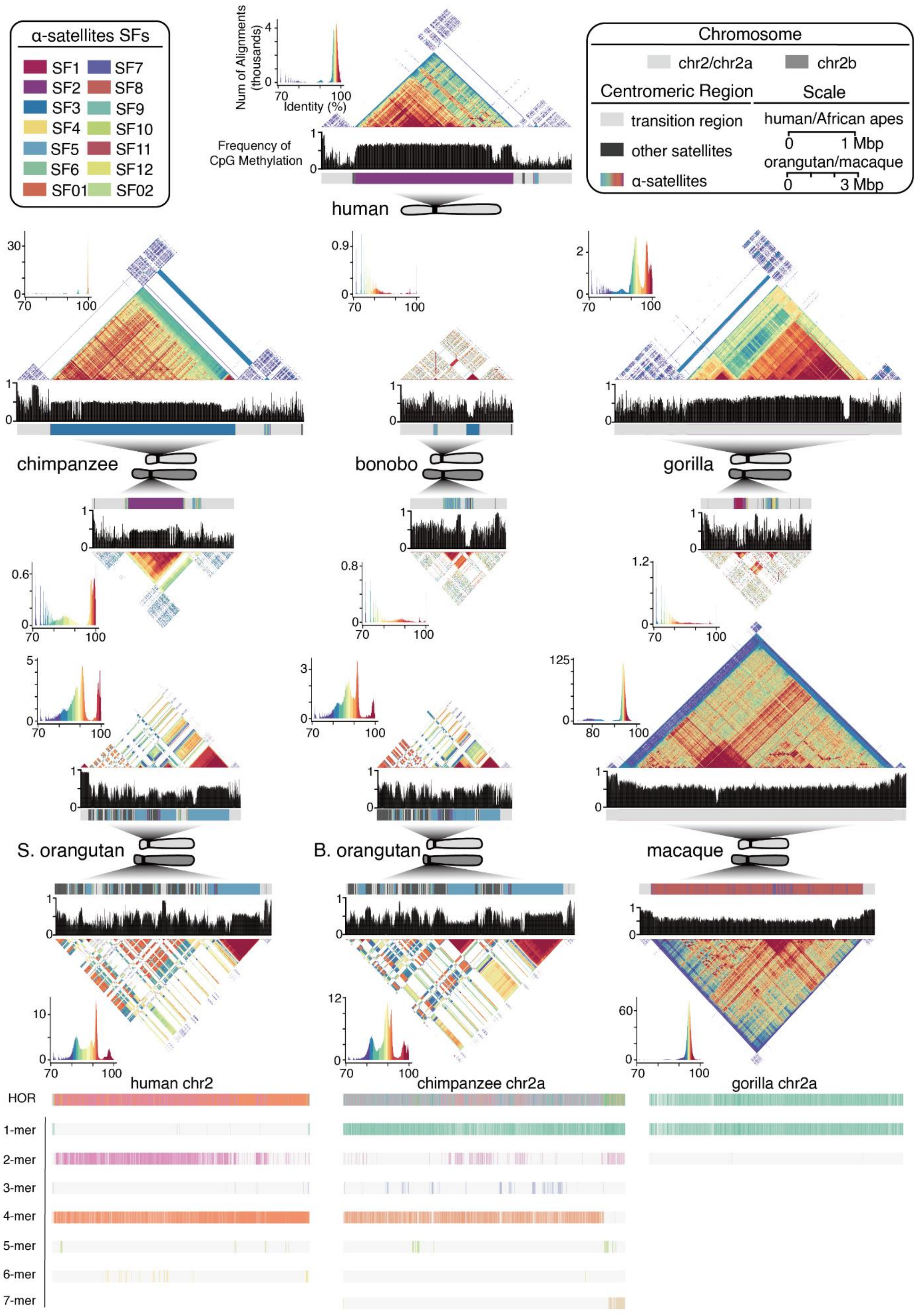
α-satellites SFs
SF1
SF2
SF3
SF4
SF5
SF6
SF01
SF7
SF8
SF9
SF10
SF11
SF12
SF02
Num of Alignments (thousands)
Identity (%)
Frequency of CpG Methylation
human
Chromosome
chr2/chr2a
chr2b
Centromeric Region
transition region
other satellites
α-satellites
Scale
human/African apes
0
1 Mbp
orangutan/macaque
3 Mbp
chimpanzee
bonobo
gorilla
S. orangutan
B. orangutan
macaque
human chr2
chimpanzee chr2a
gorilla chr2a
HOR
1-mer
2-mer
3-mer
4-mer
5-mer
6-mer
7-mer

Figure S22. Sequences and structures of centromeres in NHP chr2, related to Figure 4. Comparison of genomic structure, SF organization, and methylation landscape of α-satellite region flanking 500 kbp in human chr2, NHP chr2a, and NHP chr2b. Higher-order repeats (HOR) annotation of human chr2, chimpanzee chr2a and gorilla chr2a. Though the compositions of SFs in human and gorilla chr2a are similar (SF2, Figure 4A) but the organizations of monomers in HORs are different. Human chr2 centromeric array corresponds to a cluster of 2- and 4-monomers α-satellite HORs while gorilla chr2a centromeric array is rich of 1-monomer α-satellite HORs.

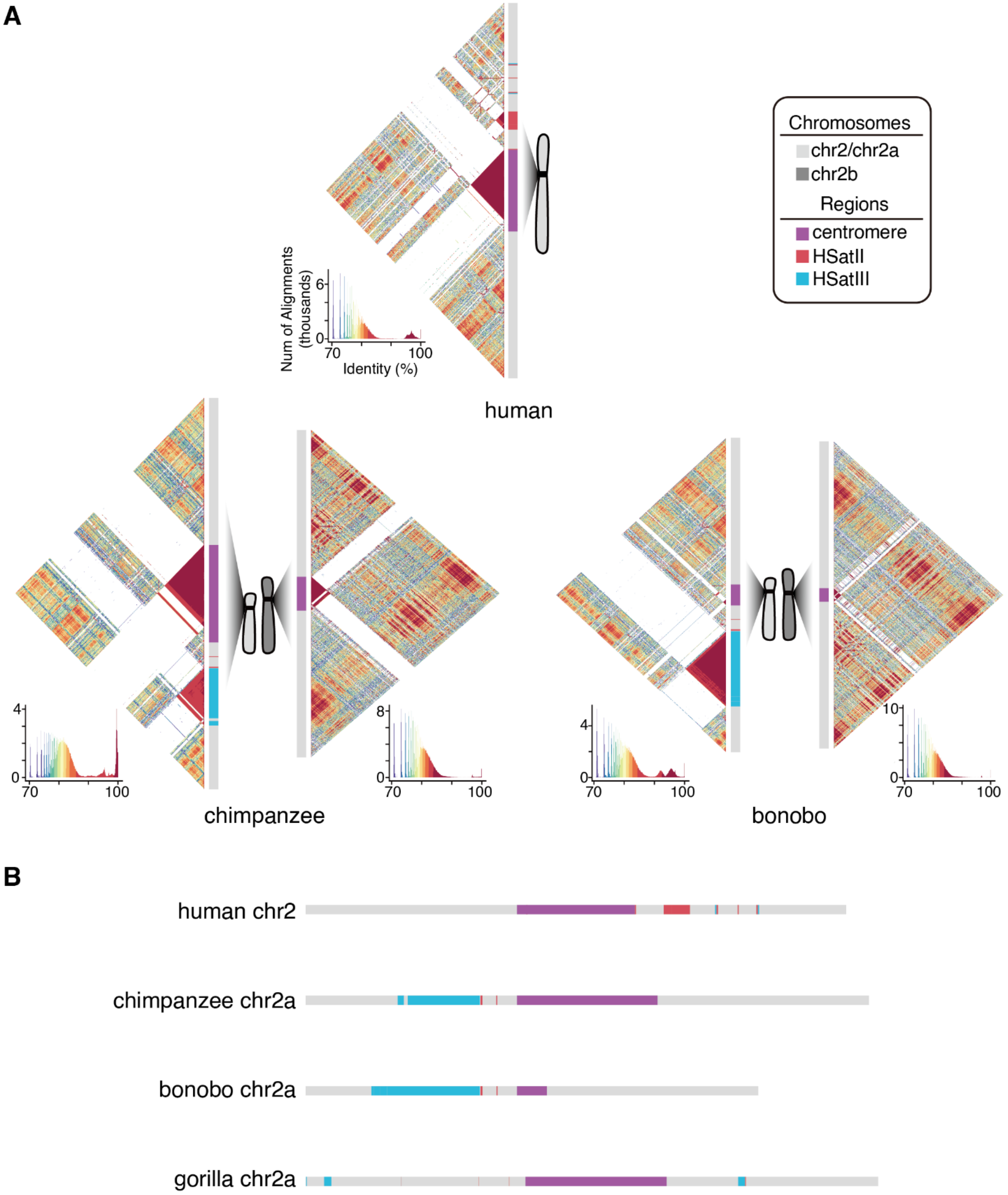

Figure S23. Structures of pericentromeric regions of human and *Pan*, related to Figure 4. (A) Genomic structure and specific satellite repeats in pericentromeric regions, including the α-satellite-rich regions and 5 Mbp on the p-arm and q-arm. Pink blocks represent the HSatII arrays and blue blocks represent the HSatIII arrays. (B) The satellite annotation of chromosome 2 in humans and chromosome 2a in chimpanzee, bonobo and gorilla.

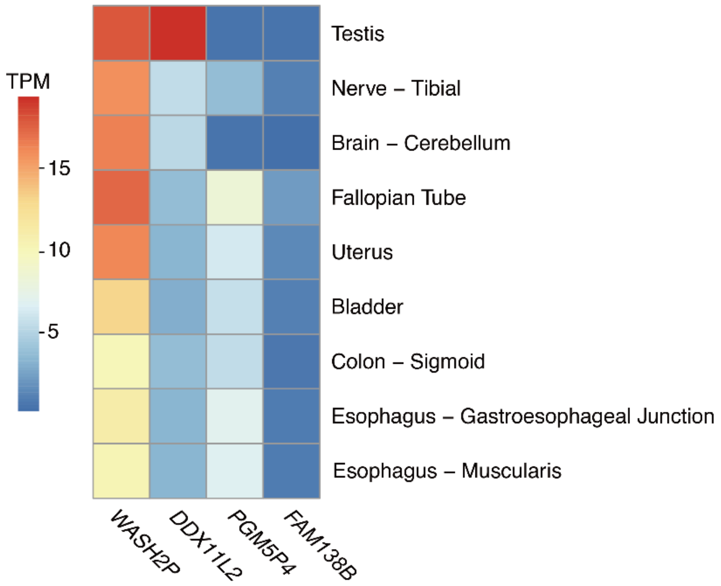

Figure S24. Expression levels of four noncoding genes in top expressed tissues, related to Figure 5. The median transcripts per million (TPM) from the GTEx database is used as the gene's expression level.

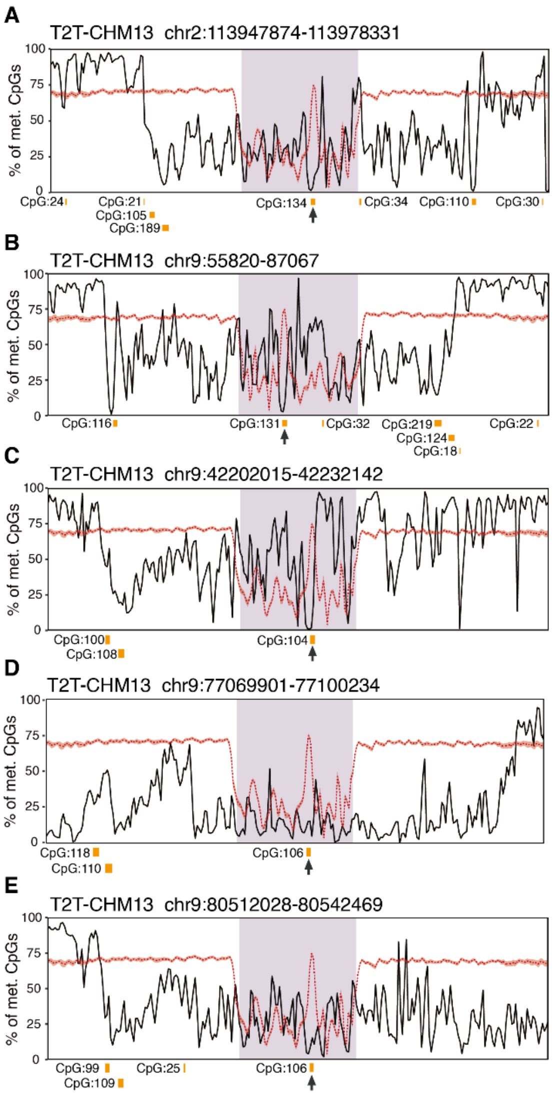

Figure S25. DNA methylation of CpG island in T2T-CHM13 *Pan* SD spacer, related to Figure 5. (A-E) The panels show the methylation status of the SD spacers in the human T2T-CHM13 genome as well as 50 kbp upstream/downstream regions. The violet shadow marks the SD spacer. The orange dashed line and black line show the average methylation level of the SD spacers in chimpanzee chr2b subtelomeric repetitive region (n=45) and methylation of this region, respectively. The lower panels under the methylation track show the CpG track from the UCSC Genome Browser and the CpG islands pointed by the arrows are significantly hypomethylated in human genome (excluding (D) chr9:77,069,901-77,100,234) but hypermethylated in *Pan* subtelomeric repetitive regions.

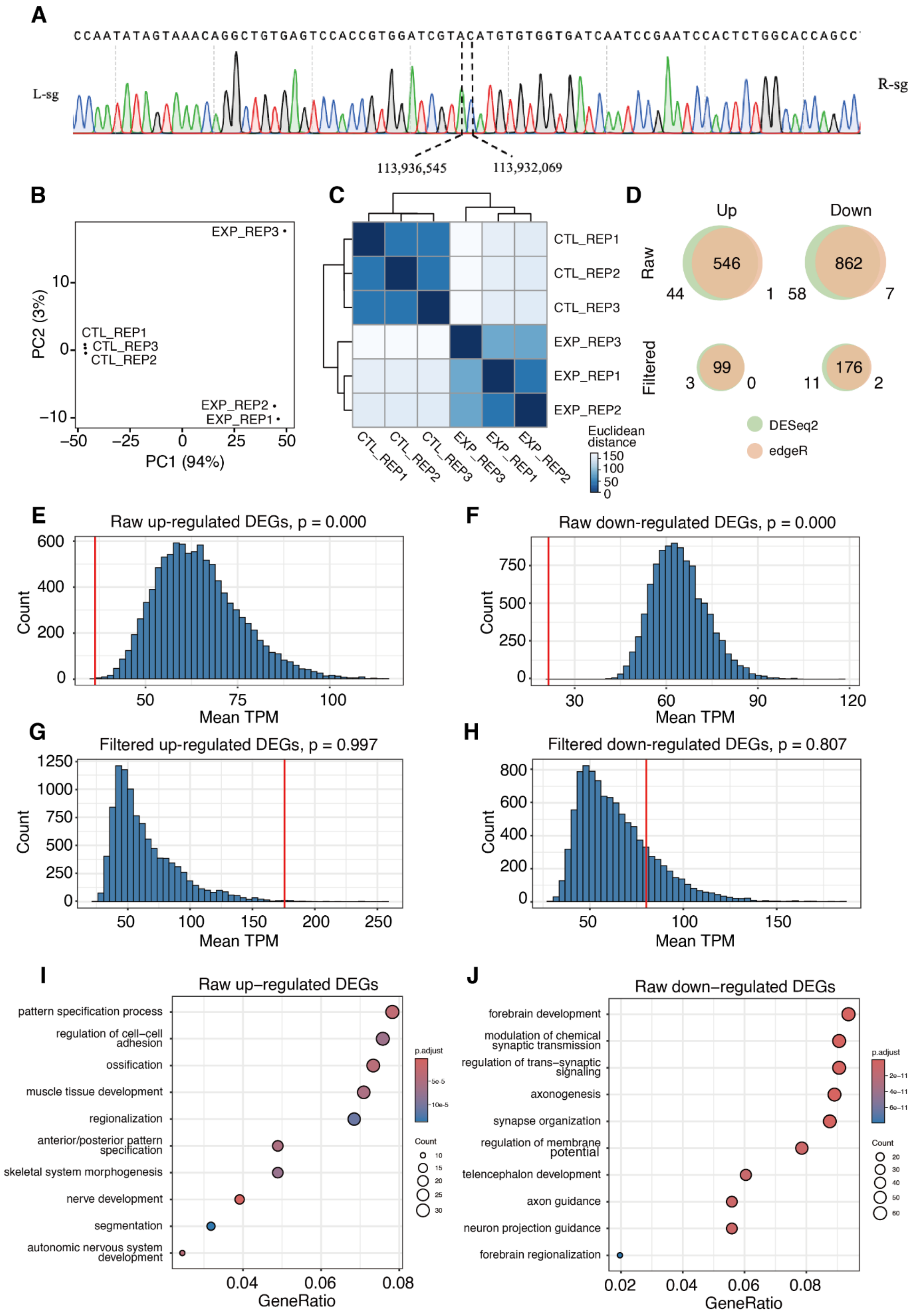

A
CCAATATAGTAAACAGGCTGTGAGTCCACCGTGGATCGTACATGTGTGGTGATCAATCCGAATCCACTCTGGCACCAGCC
L-sg
R-sg
113,936,545
113,932,069
B
EXP_REP3
CTL_REP1
CTL_REP3
CTL_REP2
EXP_REP2
EXP_REP1
PC2 (3%)
PC1 (94%)
C
CTL_REP1
CTL_REP2
CTL_REP3
EXP_REP3
EXP_REP1
EXP_REP2
Euclidean distance
D
Up
Down
Raw
Filtered
546
44
1
862
58
7
99
3
0
176
11
2
DESeq2
edgeR
E
Raw up-regulated DEGs, p = 0.000
F
Raw down-regulated DEGs, p = 0.000
G
Filtered up-regulated DEGs, p = 0.997
H
Filtered down-regulated DEGs, p = 0.807
Count
Mean TPM
I
Raw up−regulated DEGs
pattern specification process
regulation of cell−cell adhesion
ossification
muscle tissue development
regionalization
anterior/posterior pattern specification
skeletal system morphogenesis
nerve development
segmentation
autonomic nervous system development
p.adjust
Count
GeneRatio
J
Raw down−regulated DEGs
forebrain development
modulation of chemical synaptic transmission
regulation of trans−synaptic signaling
axonogenesis
synapse organization
regulation of membrane potential
telencephalon development
axon guidance
neuron projection guidance
forebrain regionalization
p.adjust
Count
GeneRatio

Figure S26. RNA-seq analysis of CN1-derived neural progenitor cells, related to Figure 5. (A) Sanger sequencing result of the depleted fusion site. (B) Principal component analysis (PCA) of RNA-seq replicates using the top 500 most expressed genes shows strong replication within each condition. (C) The Euclidean distance matrix illustrates the consistency within groups and large differences between groups. (D) Comparison of raw and filtered differentially expressed genes (DEGs) identified by DESeq2 and edgeR. Venn diagrams show that while DESeq2 identified more DEGs than edgeR in both upregulated and downregulated categories, most DEGs were identified by both tools, demonstrating high consistency between two results. Permutation test on the mean transcript per million (TPM) of the raw upregulated (E) and downregulated DEGs (F) shows the low expression level of identified DEGs. Permutation test on the mean TPM of the filtered upregulated (G) and downregulated DEGs (H) indicates that there is no clear evidence of low expression for the filtered DEGs. GO enrichment analysis of raw upregulated genes (I) and downregulated genes (J) reveals that upregulated genes are associated with the pattern specification process, while downregulated genes are linked to neuronal development and organization process. The results before filtering and after filtering are highly consistent.

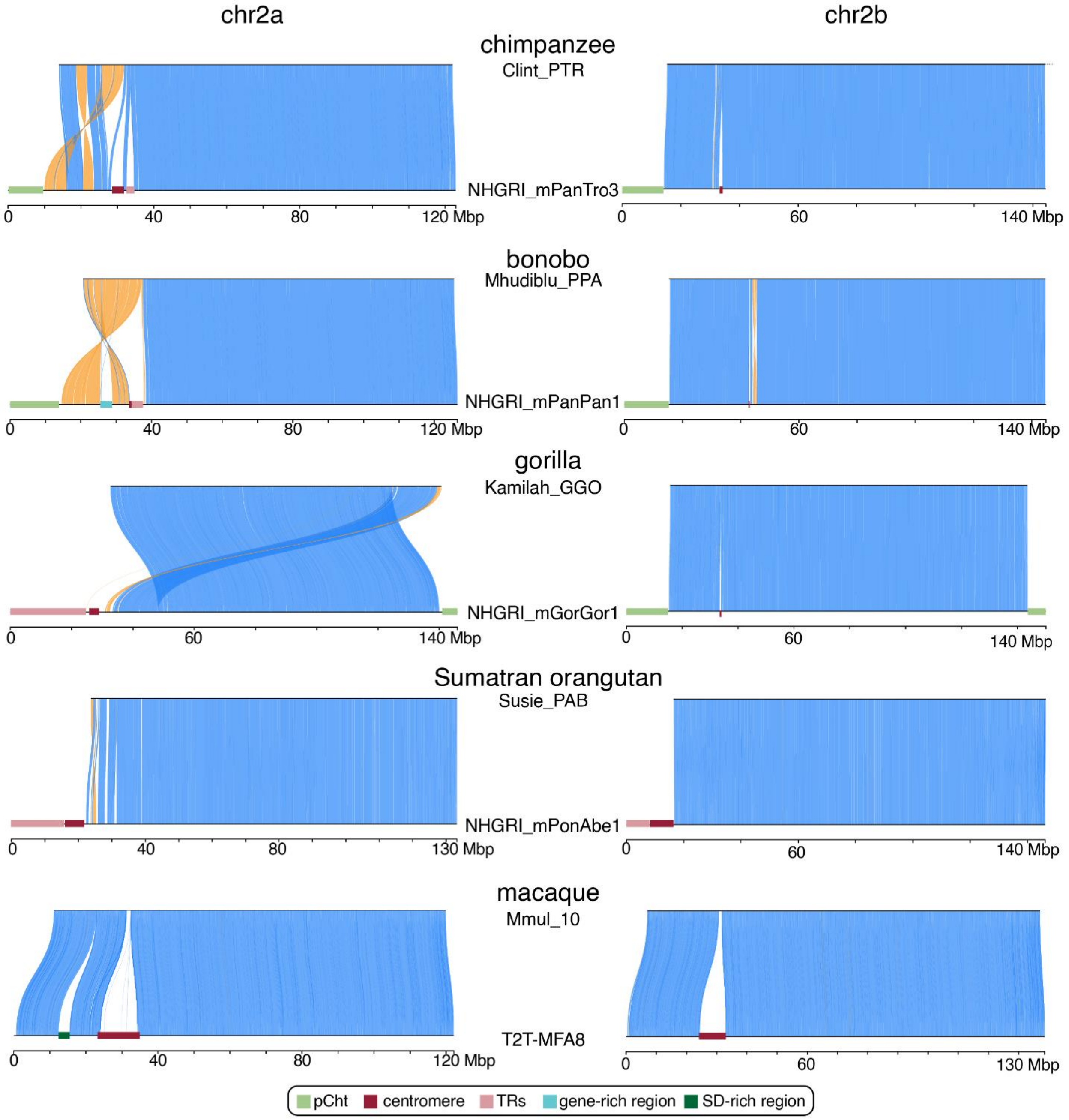

Figure S27. The chr2a/chr2b syntenic comparisons between previous genomes and T2T genomes in NHPs, related to Figure 6. The blue blocks show the syntenic alignments while the yellow blocks represent the inverted alignments. The red and pink blocks represent the previous unresolved regions. In addition, we provided more detailed analysis of the subtelomeric regions, including the SD_fusion_A/B/C on NHP genomes. We found 63 out of 74 SDs homologous to the human fusion site in T2T NHP genomes were not resolved in previous NHP genome assemblies (Table S14).

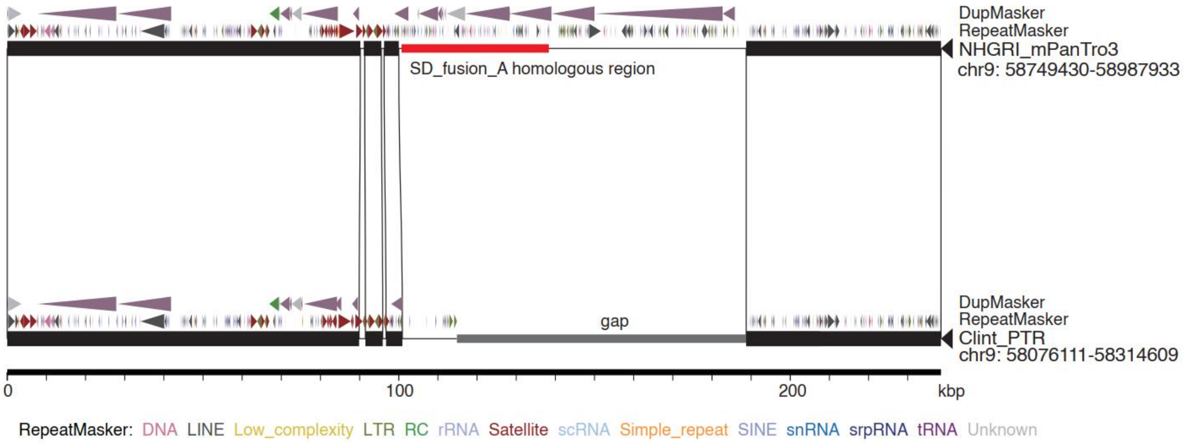

Figure S28. The comparison of a region containing SD_fusion_A in chromosome 9 between chimpanzee T2T genome and previous genome, related to Figure 6. The red block represents the SD_fusion_A homologous region in the chimpanzee T2T genome, while it wasn't assembled in previous genome. The gray block represents the gap in this region (scaffolded by N).

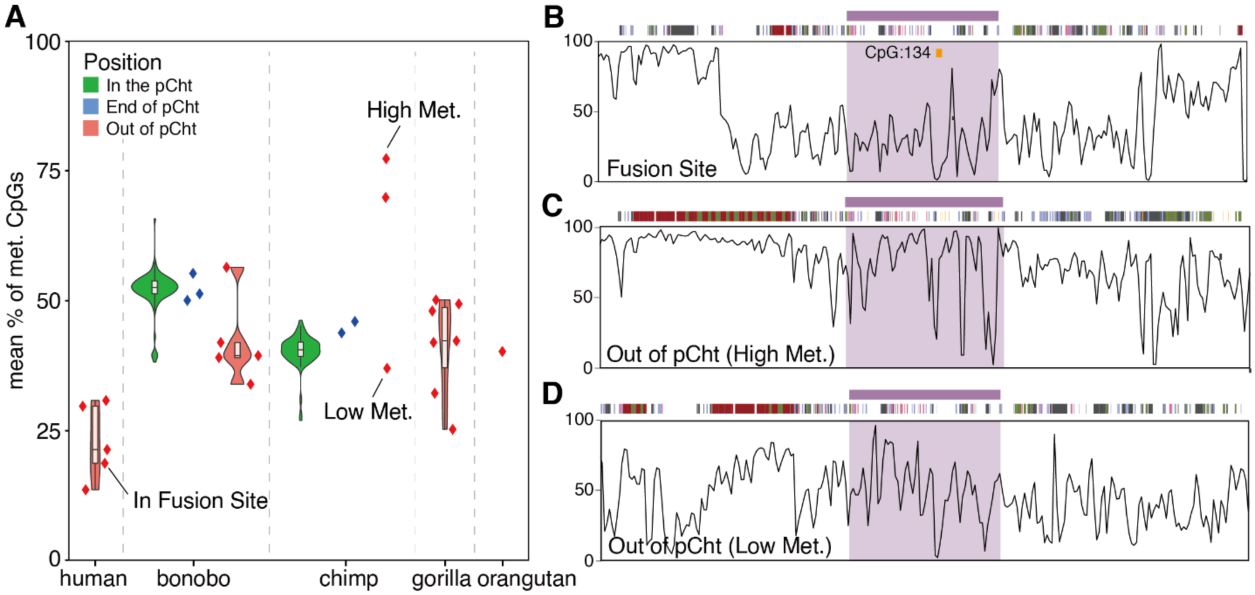

Figure S29. DNA methylation levels of SD spacers in different positions in human and nonhuman primates, related to Figure 6. (A) DNA methylation (Met.) levels of SD spacers in different positions in human and great apes. The methylation profiles of SD spacers in the fusion site (B), hypermethylated SD spacer out of subtelomeric repetitive region (C), and hypomethylated SD spacer out of subtelomeric repetitive region (D) are depicted.

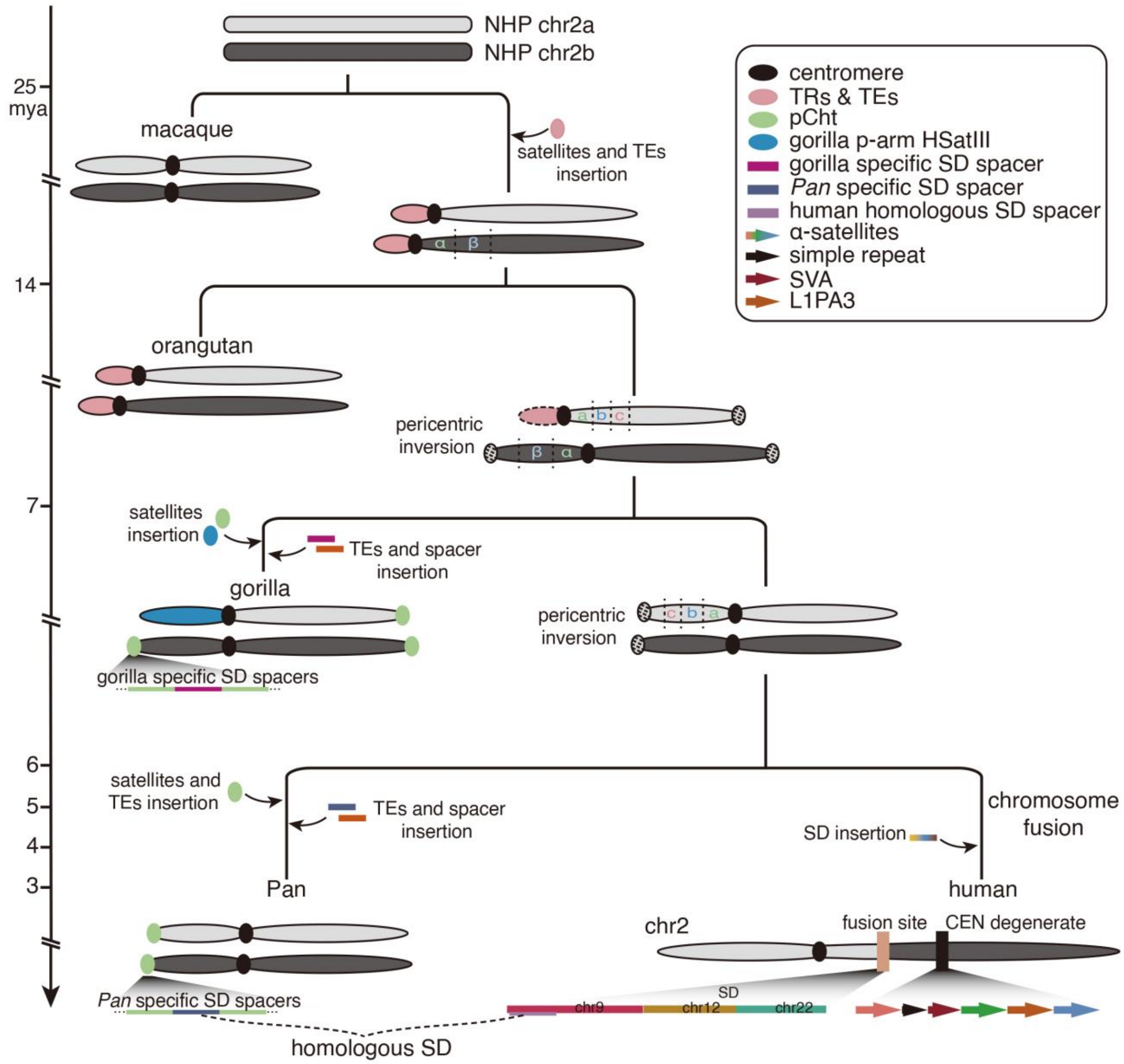

Figure S30. Model for chromosome 2 evolution, related to Figure 6. The NHP chr2a (light grey) and chr2b (dark grey) represent the karyotypes of each primate lineage and their reconstructed common ancestors. In orangutans, lineage-specific tandem repeats (TRs) and transposable elements (TEs) (pink) formed acrocentric chr2a and chr2b. A pericentric inversion on chr2b of the human–*Pan*–gorilla common ancestor (α, β vs. β, α) and specific TRs (blue for HSatIII, green for pCht) and gorilla-specific SD spacers turnover lead to the gorilla-specific karyotype. A subsequent inversion (a,b,c vs. c,b,a) in the human–*Pan* ancestor created an "unstable" chromosome structure. In the *Pan* lineage, specific TRs (green for pCht) and *Pan*-specific SD spacers result in *Pan*-specific subtelomeric repetitive structures of chr2a and chr2b. While human-specific SD insertions (pink for chr9 SD pairs, yellow for chr12 SD pairs, and green for chr22 SD pairs) were involved in the human chr2 fusion event. The SD spacers in *Pan* and the SD_fusion_A at the human fusion site have highly similar sequences, originating from a genomic region in the human–*Pan*–gorilla common ancestor. The three retained α-satellites are also shown with colorful arrows. The uncertain subtelomeric repetitive caps are shown with grey ovals with dashed lines.
